# Electroacupuncture reduces inflammatory damage following cerebral ischemia–reperfusion by enhancing ABCA1-mediated efferocytosis in M2 microglia

**DOI:** 10.1186/s13041-024-01135-0

**Published:** 2024-09-02

**Authors:** Yu-sha Liao, Tie-chun Zhang, Yu-qi Tang, Pei Yu, Ya-ning Liu, Jing Yuan, Ling Zhao

**Affiliations:** 1 Acupuncture and Tuina School, Chengdu University of Traditional Chinese Medicine, No. 1166 Liutai Avenue, Chengdu, 611137 Sichuan China; 2 Key Laboratory of Acupuncture for Senile Disease (Chengdu University of TCM), Ministry of Education, Chengdu, 611137 Sichuan China; 3 Clinical Research Center for Acupuncture and Moxibustion in Sichuan Province, Chengdu, 610075 China

**Keywords:** Electroacupuncture, Cerebral ischemia/reperfusion injury, Efferocytosis, Microglia, Abca1

## Abstract

Ischemic stroke (IS) is a severe cerebrovascular disease with high disability and mortality rates, where the inflammatory response is crucial to its progression and prognosis. Efferocytosis, the prompt removal of dead cells, can reduce excessive inflammation after IS injury. While electroacupuncture (EA) has been shown to decrease inflammation post-ischemia/reperfusion (I/R), its link to efferocytosis is unclear. Our research identified ATP-binding cassette transporter A1 (Abca1) as a key regulator of the engulfment process of efferocytosis after IS by analyzing public datasets and validating findings in a mouse model, revealing its close ties to IS progression. We demonstrated that EA can reduce neuronal cell death and excessive inflammation caused by I/R. Furthermore, EA treatment increased Abca1 expression, prevented microglia activation, promoted M2 microglia polarization, and enhanced their ability to phagocytose injured neurons in I/R mice. This suggests that EA's modulation of efferocytosis could be a potential mechanism for reducing cerebral I/R injury, making regulators of efferocytosis steps a promising therapeutic target for EA benefits.

**Supplementary Information:**

The online version contains supplementary material available at 10.1186/s13041-024-01135-0.

## Introduction

IS is a disease caused by decreased cerebral blood flow, resulting in damage to brain tissue in the area supplied with blood, and accounts for approximately 62.4% of all stroke cases and 87% of all stroke deaths [1, 2]. The primary clinical interventions for IS presently involve intravenous thrombolysis with tissue plasminogen activator (tPA) and endovascular thrombectomy [3]. However, both therapies are limited by a strict time window, and revascularization may still lead to neurological damage known as cerebral I/R injury [4, 5]. Inflammatory injury is the primary pathological alteration in I/R injury, exacerbating secondary brain injury mainly by causing blood–brain barrier disruption, brain edema, and oxidative stress [6, 7]. This injury severely affects the prognosis of stroke patients and could exacerbate both disability and mortality rates [8, 9].

Microglia are the primary immune cells in the brain, serving as the first line of defense in protecting the nervous system against damage [10]. Two distinct polarization phenotypes are identifiable in microglia activated after ischemic stroke: the pro-inflammatory M1, which exerts neurotoxic effects, and the anti-inflammatory M2, which is neuroprotective [11]. Recent studies demonstrate that the process of phagocytosis and removal of dead or dying cells and debris, also known as efferocytosis, is crucial for microglia in exerting neuroprotective effects and promoting post-ischemic brain tissue repair [12, 13]. Efferocytosis helps prevent the release of damage-associated molecular patterns (DAMPs) and reduces the exaggerated inflammatory response, which is essential for restructuring neural circuits and the central microenvironment following I/R injury [14]. Additionally, impaired microglial efferocytosis results in an exacerbated inflammatory response that hinders the neurological recovery following I/R injury [15]. Therefore, a deep study of the function of microglial efferocytosis represents a novel approach to I/R therapy.

As a traditional Chinese therapy with thousands of years of history, acupuncture has been widely used to treat ischemic stroke, due to its safety and effectiveness [16, 17]. The existing research evidence suggests that EA, which combines traditional acupuncture with electrical stimulation, can enhance treatment outcomes and mitigate cerebral I/R injury and associated neurological deficits by suppressing the inflammatory response [18–20]. However, it remains unclear whether efferocytosis, a crucial process in regulating inflammatory responses during cerebral ischemic injury, serves as the primary mechanism by which EA modulates the anti-inflammatory effects of microglia. In the present study, we used bioinformatics methods to screen genes associated with efferocytosis after cerebral ischemic injury. In addition, a middle cerebral artery occlusion/reperfusion (MCAO/R) model has been established to mimic I/R injury, and western blot (WB), reverse transcription-quantitative PCR (RT-qPCR) and Immunofluorescence (IF) analyses were performed to explore the relationship between EA and efferocytosis in promoting functional recovery after cerebral ischemia/reperfusion.

## Methods

### Bioinformatic analysis

The gene list in the article for the three processes of efferocytosis, "find me", "eat me", and "engulf me", was created by combining references to gene lists associated with efferocytosis [21–23]. The data for the bioinformatic analyses were obtained from the public repository NCBI GEO (http://www.ncbi.nlm.nih.gov/geo), and datasets GSE30655 and GSE174574 were used in the study. The protein–protein interaction network (PPI) of differentially expressed genes (DEGs) associated with efferocytosis was generated using the STRING database and visualized using Cytoscape. The single-cell RNA-sequencing (scRNA-seq) dataset was visualized using UMAP plots.

### Animals and groups

C57BL/6J mice were used in this study (male, aged 6–8 weeks, weighing 20–25 g), and were supplied by GemPharmatech Co., Ltd., Chengdu, China (license No. SCXK (Chuan) 2020-034). Mice were housed in well-ventilated cages under 12 h light/dark cycle at a temperature of 25 ± 1 °C and humidity of 50 ± 5% with adequate food and water. The mice were randomly and equally assigned to the sham, I/R, and EA groups, with the experimenters blinded to the groups. During the experiment, any mice that perished or did not meet the inclusion criteria were removed, and the number of mice in each group was replenished using the same modeling procedure. All animal experimentation procedures were approved by the Animal Ethics Committee of the Hospital of Chengdu University of Traditional Chinese Medicine (No.2023DL-022).

### Mouse model of I/R

The I/R model was established using a modified Zea-Longa method [24]. We anesthetized the mice in an anesthesia induction box containing 5% isoflurane in oxygen/air mixture, then transferred them to an anesthesia mask to maintain anesthesia with 2% isoflurane (RWD Life Science, Shenzhen, China) from the animal anesthesia machine (R500, RWD Life Science, Shenzhen, China). The median neck muscle was divided, exposing the left common carotid artery (CCA), external carotid artery (ECA), and internal carotid artery (ICA). Litigation of the CCA proximal end and the ECA was performed permanently and ICA distal ends clamped with arterial clips to temporarily block blood flow to the brain. Then a monofilament (MSMC21B120PK50, RWD Life Science, Shenzhen, China) was placed inside the internal carotid artery by making an incision in the left common carotid artery to occlude the origin of the middle cerebral artery. MCAO for 60 min was followed by suturing of the wound to restore blood flow. During surgery, a heating pad was adopted to maintain the mice's body temperature at 37 °C. The sham group exposed only the left side of the vessel without monofilament insertion.

### EA treatment

EA was given to the mice in the EA group 24 h after I/R. The mice were anesthetized using a mask with 2% isoflurane and then inserted with stainless acupuncture needles (0.18 mm × 13 mm, Huatuo, Suzhou, China) into the right ST36 (Zusanli, located longitudinally at 3 cun below the knee joint and intersecting the middle of the tibialis anterior muscle) and GV20 (Baihui, located at the intersection of the sagittal midline with the line between the ears). The parameters of the EA consisted of dispersive waves with a frequency of 2/15 Hz, an intensity of 1 mA, and a duration of five consecutive days with 20 min per day. The sham group and the I/R group received anesthesia only, without electroacupuncture.

### Neurological deficit assessment

After 24 h of I/R in mice, neurological deficits were measured by an investigator who was blinded to the experimental groups. The specific scoring criteria are based on the Zea Longa five-point scale, as illustrated in Table 1 [24]. Mice that scored 1 to 3 points were considered successful in modeling and each mouse was scored daily for five consecutive days.

**Table 1:** Zea-Longa score

Score	Neurologic symptom
0	No neurological deficits
1	Inability to fully extend the right front paw while the tail of the mouse was being held
2	Inability to crawl in a straight line, instead turning in circles towards the right side
3	Dumping to the right side during walking
4	Inability to walk spontaneously or loss of consciousness

### Infarct area measurement

At the end of the fifth day of neurological scoring, brain tissues were collected for 2,3,5-triphenyl tetrazolium chloride (TTC, Sangon Biotech, Shanghai, China) staining. The brain tissues were frozen at − 20 °C for 20 min. Each brain was cut into seven coronal sections at 1 mm intervals, then immersed in 2% TTC solution and incubated at 37 °C for 15 min protected from light, followed by 4% paraformaldehyde solution (Servicebio, Wuhan, China) immersion. The non-ischemic tissue showed a red color, while the infarcted areas were white. The infarct area and total area of the six sections were determined using ImageJ software, and the following formula was used to calculate the percentage of infarct area:( total infarct area/total area of slice) × 100%.

### Histopathological observation

After 4% paraformaldehyde fixation, the brain containing hippocampal tissue was dehydrated, embedded in paraffin, and coronally sectioned. Sections were stained with Nissl staining solution (Servicebio, Wuhan, China) and Hematoxylin–Eosin staining kit (Servicebio, Wuhan, China), respectively. A light microscope (Nikon Eclipse E100, Nikon, Tokyo, Japan) was used to examine the pathological changes of cortex and hippocampus tissues.

### Multiplex immunofluorescence staining

Paraffin-embedded sections were washed in distilled water after they had been deparaffinized. The antigen was repaired using a microwave with ethylene diamine tetraacetic acid (EDTA, PH8.0, C1033, Solarbio, Beijing, China) antigen repair buffer. Sections were pretreated with 3% H_2_O_2_ for 10 min to block endogenous peroxidase activity and incubated in 3% (w/v) bovine serum albumin-V (BSA-V, A8020, Solarbio, Beijing, China) in Phosphate Buffered Saline (PBS, Servicebio, Wuhan, China) for 30 min at room temperature (RT). The following antibodies were used as primary antibodies at 1:200 dilution in PBS overnight at 4 °C: anti-Iba1 (ab178846, Abcam, Cambridge, UK), anti-CD206 (GB113497-100, Servicebio, Wuhan, China), anti-NeuN (A19086, ABclonal, Wuhan, China), and anti-Abca1 (A21976, ABclonal, Wuhan, China). Wash with PBS and incubate with anti-rabbit IgG antibody 1: 400 (FCMCS, FMS-Rb01) for 50 min at RT in the dark. The following fluorescent dyes were applied to the sections: IF488-Tyramide (G1236-1, Servicebio, Wuhan, China), IF555-Tyramide (G1236-2, Servicebio, Wuhan, China), and IF647-Tyramide (G1236-3, Servicebio, Wuhan, China). Incubations were shielded from light for 10 min. Multiplex staining was repeated in series for each staining step, and antigen repair was performed between each staining step. The sections were subsequently incubated with 4′,6-diamidino-2-phenylindole (DAPI, G1236-5, Servicebio, Wuhan, China) for 10 min at RT in the dark, washed, and sealed with an anti-fluorescence quenching sealer (G1401-5, Servicebio, Wuhan, China) finally. These brain sections were observed with Nikon Eclipse E100. ImageJ software was used to count the number of positive cells.

### Western blot

The hippocampus and parietal cortex were extracted from the mouse brain and weighed, and a BCA protein assay kit (BL521A, Biosharp, Beijing, China) was used to determine protein concentration. Each sample contained 15–50 μg of protein, which was electrophoresed on a sodium dodecyl sulfate–polyacrylamide gel and then transferred to PVDF membranes (IPVH00010, 0.45 µm, Millipore, Immobilon, Ireland). The membranes were blocked with 5% (w/v) skimmed milk in tris buffered saline with Tween-20 (TBST) buffer for 2 h. After washing with TBST, the membranes were incubated at 1:1000 dilution in TBST overnight at 4 °C with the following antibodies: anti-Iba1 (ab178846, Abcam, Cambridge, UK), anti-CD206 (A8301, ABclonal, Wuhan, China), anti-NeuN (A19086, ABclonal, Wuhan, China), anti-Abca1 (A21976, ABclonal, Wuhan, China), anti-GAPDH(GB12002, Servicebio, Wuhan, China). After washing with TBST, the secondary antibody diluent of the corresponding species of the primary antibody, goat anti-rabbit IgG antibody (FMS-Rb01, FCMCS, Nanjing, China) or mouse (FMS-MS01, FCMCS, Nanjing, China), was added and incubated for 1.5 h at RT. An enhanced chemistry luminescence (ECL, BL520A, Biosharp, Beijing, China) method was used to detect protein bands after the membranes were again washed. The intensity of the bands was analyzed using ImageJ software.

### RNA extraction and RT-qPCR analysis

The cortex and hippocampus of the mice were promptly flash-frozen in liquid nitrogen and stored at − 80 °C after extraction. Total RNA was extracted using the MolPure Cell/Tissue Total RNA Kit (Yeasen Biotechnology, Shanghai, China), and purity and concentration were determined using the Nanodrop 2000 UV–visible spectrophotometer. RNA is reverse transcribed to cDNA using Hifair III 1st Strand cDNA Synthesis SuperMix for qPCR (Yeasen Biotechnology, Shanghai, China). Subsequently, RT-qPCR analysis was conducted utilizing the primers listed in Table 2, based on the Hieff UNICON® Universal Blue qPCR SYBR Green Master Mix (Yeasen Biotechnology, Shanghai, China). For detailed procedures, please refer to the corresponding kit instructions. The primer sequences utilized for amplification are displayed in Table 2, provided by Sangong Biotech (Sangong Biotech, Shanghai, China).

**Table 2:** RT-qPCR primer sequences

Primer	Sequences (5′–3′)
Iba1-F	ATCAACAAGCAATTCCTCGATGA
Iba1-R	CAGCATTCGCTTCAAGGACATA
Cd206-F	CTCTGTTCAGCTATTGGACGC
Cd206-R	CGGAATTTCTGGGATTCAGCTTC
NeuN-F	ATCGTAGAGGGACGGAAAATTGA
NeuN-R	GTTCCCAGGCTTCTTATTGGTC
Abca1-F	AAAACCGCAGACATCCTTCAG
Abca1-R	CATACCGAAACTCGTTCACCC

### Statistical analyses

All data were analyzed using either GraphPad Prism v.9.5.0 or R software and expressed as mean ± standard error of the mean. Survival curves were compared using the Log-rank test. Tukey's multiple comparisons test was used to test for differences in percentage weight change and Neurological Deficit. Other data that followed a normal distribution with homogenous variance were analyzed using One-way analysis of variance for multiple comparisons. *P*-values less than 0.05 were deemed statistically significant.

## Results

### EA treatment improved survival outcomes, neurologic deficits, and reduced infarct area in I/R Mice

**Fig. 1:**
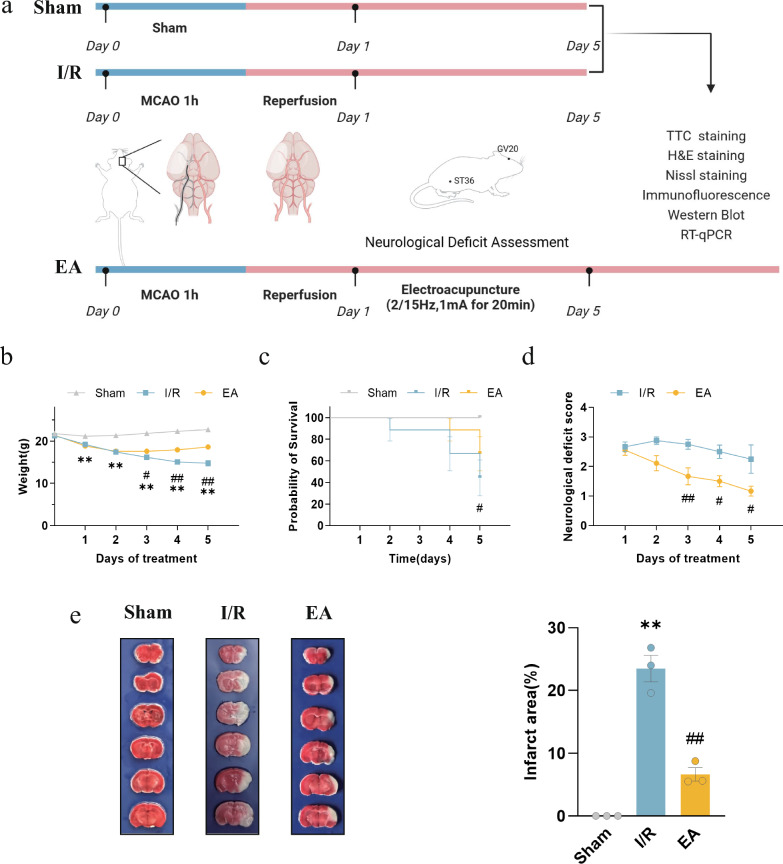
Electroacupuncture increased survival rates, improved neurologic function, and reduced cerebral infarct area in I/R Mice. **a** Flowchart of the animal experiment (n = 9/group). **b** Body weight change curve (n = 9/group). **c** Survival curve **d** Neurological deficit score (n = 9/group). **e** TTC staining images and percentage of infarct area ratio(n = 3/group). ^*^P < 0.05, ^**^P < 0.01, compared with the sham group; ^#^*P* < 0.05, ^##^*P* < 0.05, compared with the I/R group

The experimental flow chart is illustrated in Fig. 1a. To assess the protective effects of EA in mice model of I/R, we monitored weight, survival rate, neurological deficit scores, and percentage of cerebral infarct volume of the mice throughout the experimental period. The results indicated that the I/R model mice experienced a significant decline in weight and a shorter survival time compared to the sham group; compared to the I/R group, the EA group demonstrated a noteworthy deceleration in the reduction of their body weight (*P* < 0.0001) and exhibited an observable increase towards a survival rate (*P* = 0.0331) (Fig. 1b, c). Neurological impairment is one of the main adverse outcomes following I/R injury. Mice in the sham group did not show any neurobehavioral dysfunction, whereas those in the I/R group showed significant neurological deficits; EA treatment was effective in the reduction of neurological deficit scores (*P* < 0.0001) (Fig. 1d). Additionally, we assessed the infarct area through TTC staining and observed that EA intervention proved to be effective in reducing infarct injury (*P* = 0.0002) (Fig. 1e). These findings suggested that EA was effective in reducing infarct size, improving prognosis, and providing neuroprotection in I/R mice.

### EA treatment attenuated I/R-induced neuronal loss

**Fig. 2:**
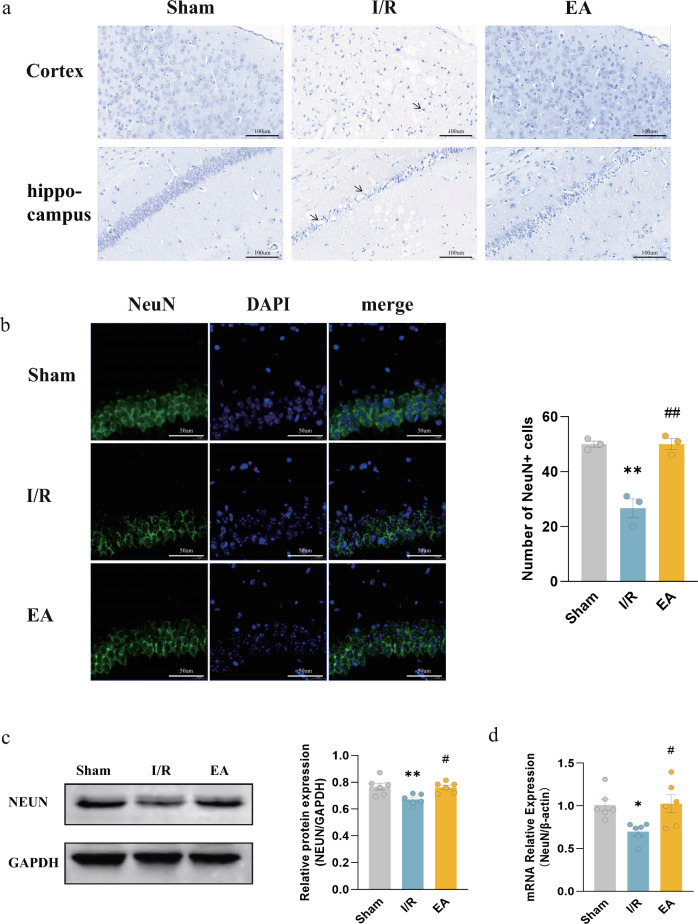
Electroacupuncture attenuated I/R-induced neuronal loss. **a** Nissl staining of the cerebral cortex and hippocampus (Arrows indicate representative damaged neurons) (bar = 100 μm). **b** IF images of NeuN (green) and quantification of the number of NeuN-positive cells in the hippocampus; nuclei were counterstained with DAPI (blue) (bar = 50 μm; n = 3/group). **c** Western blotting bands of NeuN and its relative protein level (n = 6/group). **d** RT-qPCR for the relative level of NeuN mRNA (n = 6/group). ^*^P < 0.05, ^**^P < 0.01, compared with the sham group; ^#^*P* < 0.05, ^##^*P* < 0.05, compared with the I/R group

As neuronal injury releases DAMPs that can worsen the inflammatory cascade, we assessed the impact of EA on neuronal injury induced by I/R. Pathological findings of the cerebral cortex and hippocampus were conducted using Nissl staining. The results showed that the neurons in the sham group had intact morphology, neat arrangement, and uniform staining; the number of neurons in the I/R group significantly decreased, and the histological alterations revealed abnormal morphology, disorganized arrangements, and reduced Nissl bodies; in the EA group, Nissl bodies were more abundant and neuronal damage and loss were reduced (Fig. 2a). IF staining showed that the number of NeuN-positive cells significantly decreased after I/R, whereas the EA group showed an increase in comparison with the I/R group in the hippocampal area (*P* = 0.0008) (Fig. 2b). Additionally, both WB and RT-qPCR results revealed a significant decrease in the expression of the neuronal marker NeuN at both protein and transcript levels in the brains of mice in the I/R group compared to those in the sham group; EA treatment resulted in an upregulation of NeuN expression compared to the I/R group (*P* = 0.0100 in WB, *P* = 0.0163 in PCR) (Fig. 2c, d). The results suggested that EA treatment attenuated I/R-induced neuronal loss.

### Efferocytosis-related gene Abca1 expression is upregulated after cerebral ischemia

**Fig. 3:**
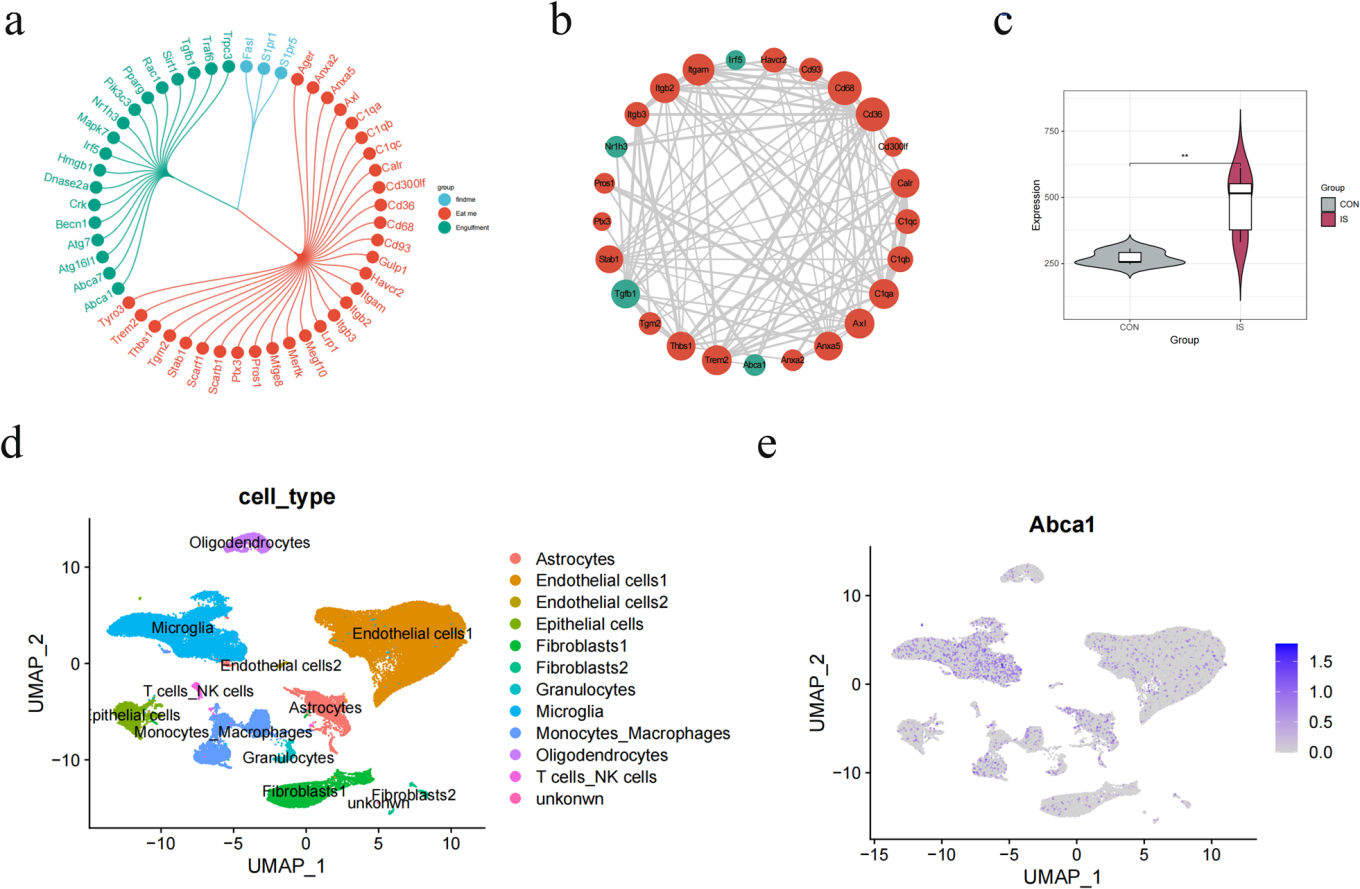
The analysis of efferocytosis-related genes from a publicly available database. **a** Summary of efferocytosis-related genes. **b** PPI network of efferocytosis-related genes. **c** The Violin plot of Abca1 expression. **d** UMAP plot, single-cell clusters colored by the cell type classification. **e** UMAP plot, showing the distribution of Abca1 expression

After cerebral ischemic injury, efferocytosis rapidly engulfs and removes dead cells, which is critical to preventing excessive inflammatory responses and reducing secondary ischemic injury [25]. Efferocytosis is a process that includes three stages: "find me", "eat me", and "engulf me", and these markers have been classified individually through an analysis of the relevant literature [21–23] (Fig. 3a). Using the STRING database, the PPI network of DEGs associated with efferocytosis was visualized in Cytoscape (Fig. 3b). Through analyzing data from a publicly available database, we discovered increased expression of Abca1, a key regulator of the engulfment process, after cerebral ischemic injury (Fig. 3c). Microglia are the primary immune cells of the central nervous system and play an important role in the regulation of central inflammation. ScRNA-seq analysis revealed a specific expression of Abca1 in the microglia (Fig. 3d, e). Based on the above results of the bioinformatics analysis, it is hypothesized that the Abca1-mediated efferocytosis process in microglia may play an important role in the reduction of inflammatory injury.

### EA treatment inhibited microglial activation and upregulated Abca1 expression

**Fig. 4:**
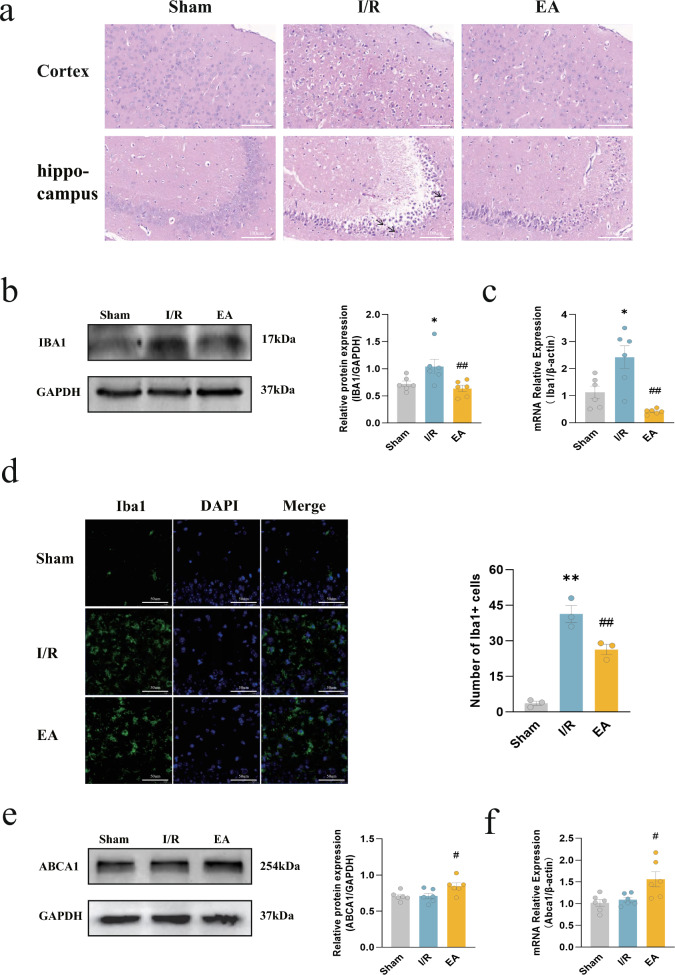
Electroacupuncture alleviated I/R inflammatory injury, suppressed microglial activation, and increased Abca1 expression. **a** HE staining of the cerebral cortex and hippocampus (Arrows indicate representative inflammatory cells) (bar = 100 μm). **b** Western blotting bands of Iba1 and its relative protein level (n = 6/group). **c** RT-qPCR for the relative level of Iba1 mRNA (n = 6/group). **d** IF images of Iba1 (green) and quantification of the number of Iba1-positive cells in the hippocampus; nuclei were counterstained with DAPI (blue) (bar = 50 μm; n = 3/group). **e** Western blotting bands of Abca1 and its relative protein level (n = 6/group); **f** RT-qPCR for the relative level of Abca1 mRNA (n = 6/group). ^*^P < 0.05, ^**^P < 0.01, compared with the sham group; ^#^*P* < 0.05, ^##^*P* < 0.01, compared with the I/R group

To investigate the aforementioned hypothesis, we employed HE staining to observe that the brain tissue of the I/R group showed significant inflammatory cell infiltration and cytoplasmic vacuolization, especially in the hippocampus region, and EA treatment effectively attenuated these pathological changes (Fig. 4a). WB and RT-qPCR were used to analyze the expression of Iba1 and Abca1 to evaluate the anti-inflammatory effects of EA in the I/R model. Iba1 expression significantly increased following I/R, while EA treatment effectively suppressed the upregulation of Iba1 (*P* = 0.0083 in WB, *P* = 0.0091 in PCR) (Fig. 4b, c). Analysis of the IF staining, it was observed that microglia in the Sham group had minimal expression while microglia in the I/R group exhibited significant activation, as indicated by an increase in the number of Iba1-positive cells and an enlargement of the cell body. This activation was successfully inhibited by EA (*P* = 0.0088) (Fig. 4d). Similarly, we analyzed Abca1 using the same methodology and found that EA significantly increased expression (*P* = 0.0379 in WB, *P* = 0.0196 in PCR), while the distinction between the I/R group and the sham group was not significant (Fig. 4e, f). The above results suggested that EA treatment could diminish inflammatory injury, and this effect was linked to microglia activation inhibition and ABCA1 expression promotion.

### EA treatment promoted neurological recovery by enhancing Abca1-mediated efferocytosis of M2 microglia

**Fig. 5:**
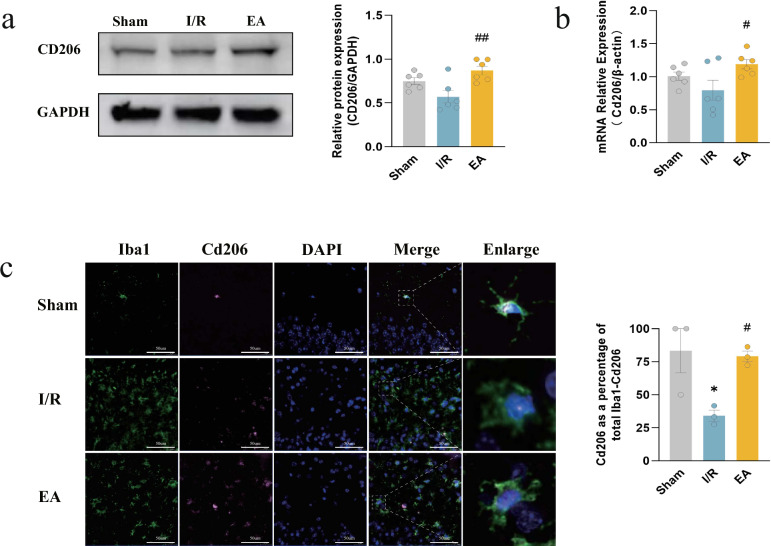
Electroacupuncture increased the expression of the CD206 marker of M2 microglia. **a** Western blotting bands of CD206 and its relative protein level (n = 6/group). **b** RT-qPCR for the relative level of CD206 mRNA (n = 6/group). **c** IF images of co-localization of Iba1 (green) and CD206 (pink) in the hippocampus and quantification of the number of Iba1-CD206 double-positive cells relative to the number of Iba1-positive cells; nuclei were counterstained with DAPI (blue) (bar = 50 μm; n = 3/group). ^*^P < 0.05, compared with the sham group; ^#^*P* < 0.05, ^##^*P* < 0.05, compared with the I/R group

Due to the important role of anti-inflammatory M2 microglia in the protection of nerves affected by IS, we examined the expression of CD206, a specific marker for M2 microglia, in all groups of mice. The results showed that EA treatment elevated both protein (*P* = 0.0030) and transcript levels of CD206 (*P* = 0.0298) (Fig. 5a, b). Immunofluorescence double-labeling of Iba1 and CD206 was performed in mouse brain sections and, consistent with previous findings, the EA group raised the proportion of Iba1-CD206 double-positive cells to the number of Iba1-positive cells in comparison to the I/R group (*P* = 0.0360) (Fig. 5c). Therefore, the increased expression of M2 microglia could be a factor in the protective effect of EA against brain injury.

**Fig. 6:**
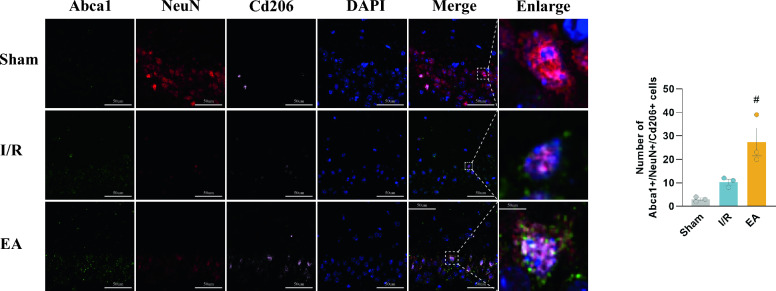
Electroacupuncture enhanced Abca1-mediated efferocytosis of M2 microglia. **a** IF images of co-localization of Abca1 (green), NeuN (red), and CD206 (pink) in the hippocampus and quantification of the number of Abca1-NeuN-CD206 triple-positive cells; nuclei were counterstained with DAPI (blue) (bar = 50 μm; n = 3/group). ^#^*P* < 0.05, compared with the I/R group

To further investigate the involvement of Abca1 expression in regulating the engulfment process of M2 microglia on injured neurons, we performed triple immunofluorescence labeling of mouse brain slices to detect the expression of Abca1, CD206, and NeuN. The IF staining results exhibited a substantial co-localization of Abca1, CD206, and NeuN in the I/R+ EA group as compared to the I/R group (*P* = 0.0243) (Fig. 6). In conclusion, EA treatment ameliorates I/R-induced neuronal damage by modulating Abca1-mediated efferocytosis in M2 microglia.

## Discussion

The inflammatory response is a significant factor in cerebral ischemic injury and is closely associated with neurological dysfunction and can lead to complications such as post-stroke infections and multiorgan dysfunction affecting the lungs, intestines, and liver in post-stroke patients [26–29]. Furthermore, a severe inflammatory response is linked with death and poor outcomes in patients with ischemic stroke [30, 31]. The biological mechanisms causing inflammation are highly complex. In cerebral I/R injury, central inflammatory responses are activated due to the ischemic cascade, and inflammatory factors of peripheral origin can directly or indirectly act on the brain as a result of the disruption of the integrity of the blood–brain barrier, leading to neuronal damage or death [32, 33]. Injured or dead neurons exacerbate secondary brain damage by releasing endogenous molecules known as DAMPs, which amplify the neuroinflammatory response [34, 35]. These outcomes can seriously impede the functional recovery of stroke patients and ultimately lead to a higher mortality rate. Therefore, it is essential to inhibit the inflammatory response following a stroke to minimize brain damage and facilitate the recovery of function. Numerous research studies have shown that acupuncture can suppress inflammation in the central nervous system (CNS) and decrease damage to the brain; it is also recognized as a means to promote the recovery of sensory and motor nerve function after stroke and improve patients' quality of life [17, 36, 37]. The ST36 and GV20 are not only frequently chosen acupoints for clinical stroke treatment but have also been reported in basic research to improve ischemic injury through multiple pathways [36, 38]. In the theory of traditional Chinese medicine, GV20 belongs to the “Du meridian”, which plays a pivotal role in regulating the functional activities of the brain. It is often the primary acupoint used in the treatment of brain disorders. The ST36 point has been shown to have the effect of unblocking the meridians and collaterals, and it is believed to improve limb dysfunction after stroke. In this study, we confirmed that EA treatment at the ST36 and GV20 could reduce infarct area and inflammatory pathological changes, decrease neuronal loss, and ameliorate neurological deficits in I/R mice.

Abca1 is a membrane protein that plays a crucial role in high-density lipoprotein production and facilitates the removal of excess intracellular cholesterol and phospholipids [39]. Previous studies have demonstrated a significant upregulation in Abca1 mRNA expression in the blood of stroke patients, and that Abca1 promotes efferocytosis and provides anti-inflammatory protection against cerebral ischemic injury [40–42]. Through analyzing previous literature and open databases, we also got the similar result. Abca1 is closely linked to the regulation of efferocytosis after cerebral ischemia and is specifically expressed in microglia. Neuroinflammation is primarily mediated by microglia, which are highly responsive to brain changes, and cerebral ischemia rapidly triggers microglial activation to migrate to the site of injury and promote damage or recovery based on the polarization phenotype [43]. M1 microglia exacerbate ischemic tissue damage by producing inflammatory cytokines and cytotoxic substances, while in contrast, M2 microglia are capable of secreting anti-inflammatory cytokines, vascular endothelial growth factor, and other substances that attenuate inflammatory damage and promote cerebral vascular reconstruction and neural recovery [44]. Previous studies have shown that EA can mitigate neuroinflammation in the I/R model by promoting the polarization of microglia to the M2 phenotype through the regulation of the STAT6/PPARγ pathway and the expression and activity of ANXA1 [45, 46]. Our study results revealed similar findings that cerebral I/R injury resulted in microglial activation and a decrease in M2 expression, but treatment with EA reversed this change. Efferocytosis serves an important function in immune regulation, and microglia are the primary cells responsible for efferocytosis in the CNS [47]. Therefore, based on bioinformatics analysis results, we further investigated the expression levels of Abca1 in the I/R model. Our results indicated that EA treatment increased Abca1 expression, suggesting that the polarization of microglia may be regulated by EA treatment through the modulation of Abca1-mediated efferocytosis processes.

Effective efferocytosis not only helps phagocytes rapidly engulf and digest dead or dying cells, thereby protecting the microenvironment from the negative consequences of decaying cells, but also mitigates the body's inflammatory response through indirect means such as reducing pro-inflammatory signals and regulating macrophage phenotypes; conversely, impaired efferocytosis may lead to secondary necrosis of apoptotic cells and subsequently trigger an immune response [48, 49]. Since M2 microglia are essential for the recovery of neurological function after I/R injury due to their pivotal role in clearing debris, controlling secondary inflammatory responses, and accelerating tissue repair [50]. To further understand the impact of Abca1 on M2 microglia efferocytosis, we performed IF analyses. The results confirmed our hypothesis, which showed an increased co-expression of M2 microglia expressing Abca1 on their membranes with neurons after electroacupuncture treatment. In summary, our research indicated that electroacupuncture might have a beneficial impact on reducing inflammation and protecting against neurological damage by facilitating the Abca1-mediated phagocytosis of injured neurons by M2 microglial cells, referred to as the efferocytosis process.

It should be noted that this study has some limitations. First, we did not use the gene silencing technique for experimental validation. Second, our investigation focused only on the impact of Abca1, a crucial regulator of the efferocytosis engulfment process, and its role in Abca1-mediated phagocytosis of injured neurons by M2 microglia after cerebral I/R injury. The direct impact of Abca1 expression on microglia activation or polarization, as well as the role of Abca1 in linking apoptotic cells to efferocytosis, require further investigation. In addition, it is uncertain whether EA can regulate the efferocytosis of other phagocytes and the interactions between various types of phagocytes by modulating Abca1 expression, as Abca1 is expressed not only on microglia but also on other phagocytes, such as astrocytes. Future studies should include a more detailed research protocol to ensure complete, reliable, and valid experimental results.

### Supplementary Information

Supplementary Material 1.

Supplementary Material 2.

Supplementary Material 3.

Supplementary Material 4.

Supplementary Material 5.

Supplementary Material 6.

Supplementary Material 7.

Supplementary Material 8.

Supplementary Material 9.

Supplementary Material 10.

Supplementary Material 11.

## Data Availability

The data sets used and/or analyzed during the current study are available from the corresponding author upon reasonable request.

## Abbreviations

IS: Ischemic stroke
I/R: Ischemia/reperfusion
EA: Electroacupuncture
tPA: Tissue plasminogen activator
DAMPs: Damage-associated molecular patterns
MCAO/R: Middle cerebral artery occlusion/reperfusion
WB: Western blot
RT-qPCR: Reverse transcription-quantitative PCR
IF: Immunofluorescence
PPI: Protein–protein interaction network
DEGs: Differentially expressed genes
scRNA-seq: Single-cell RNA-sequencing
CCA: Common carotid artery
ECA: External carotid artery
ICA: Internal carotid artery
TTC: 2,3,5-Triphenyl tetrazolium chloride
PBS: Phosphate buffered saline
RT: Room temperature
TBST: Tris buffered saline with Tween-20
CNS: Central nervous system
Abca1: ATP-binding cassette transporter A1
