# Supplementary figures and images for: Electroacupuncture reduces inflammatory damage following cerebral ischemia–reperfusion by enhancing ABCA1-mediated efferocytosis in M2 microglia
Source: Mol Brain. 2024 Sep 2;17:61. doi: 10.1186/s13041-024-01135-0 (PMC11367741; doi:10.1186/s13041-024-01135-0)

**The sham group:**

(1)
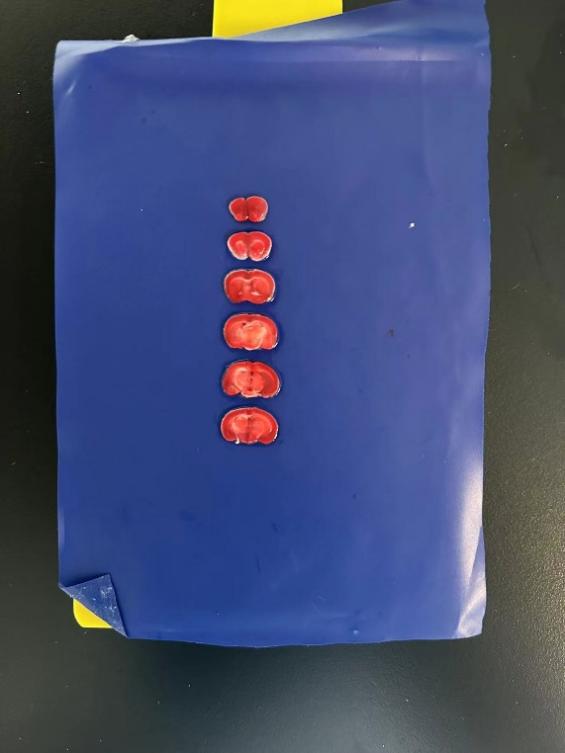
(2)
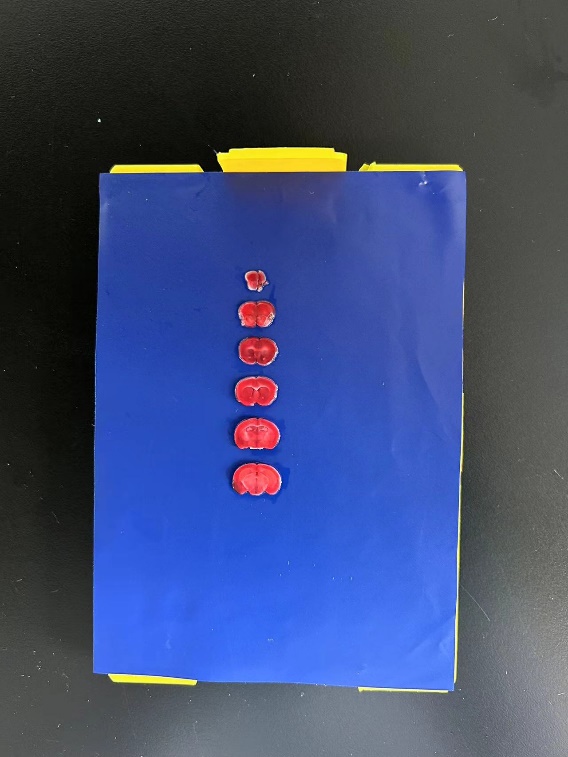


(3)
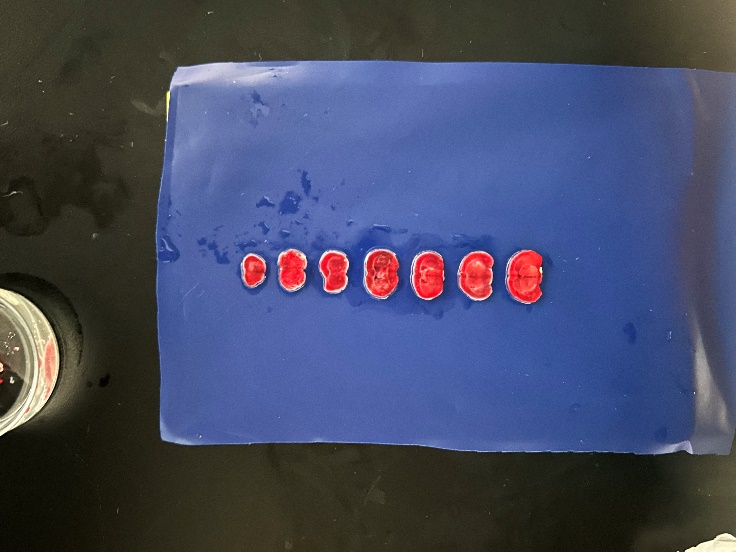


**The I/R group:**

(1)
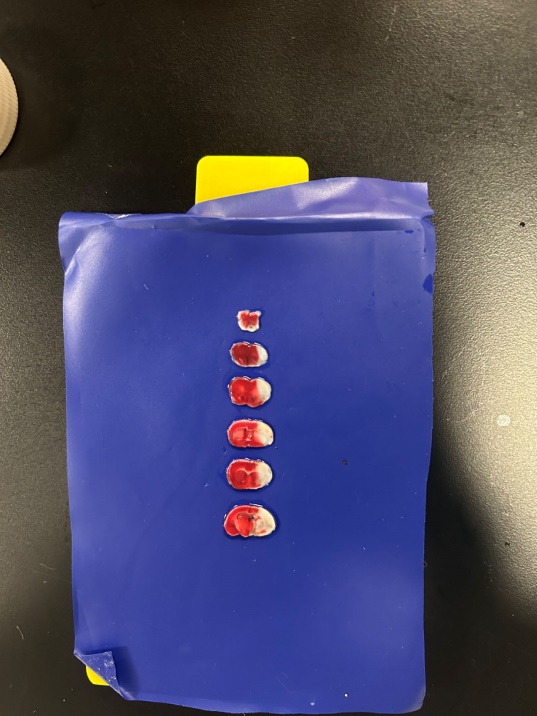
(2)
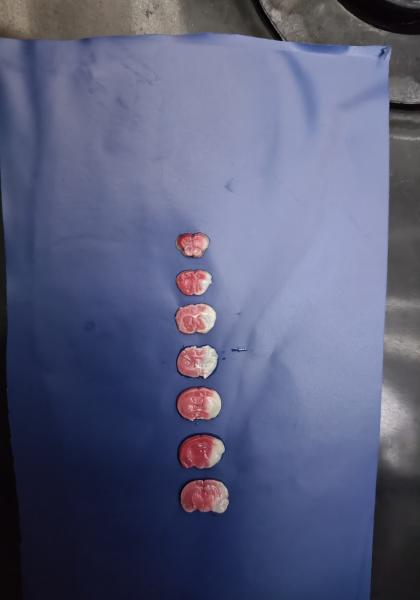


(3)
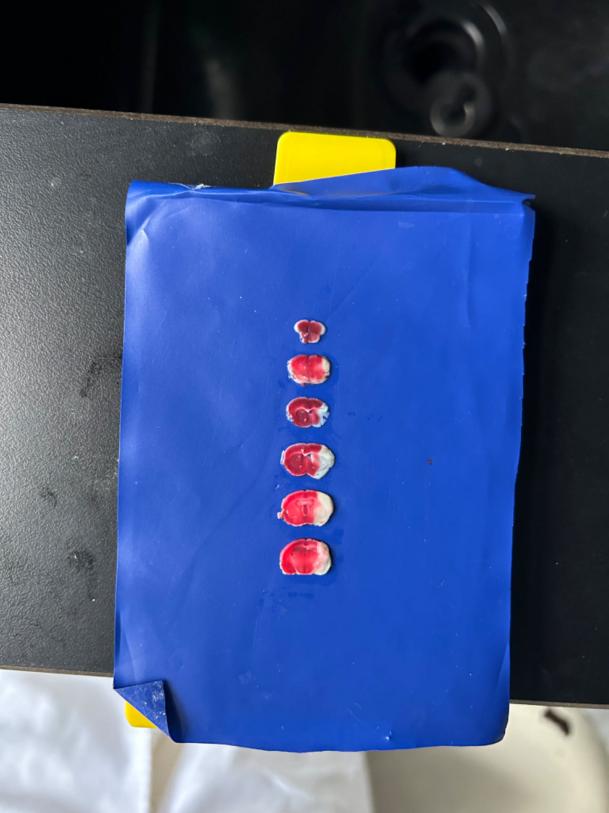


**The EA group:**

(1)
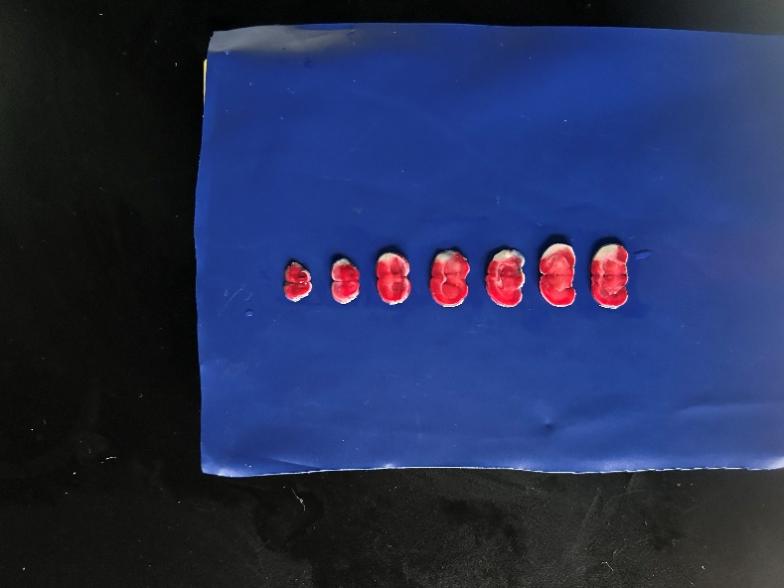
(2)
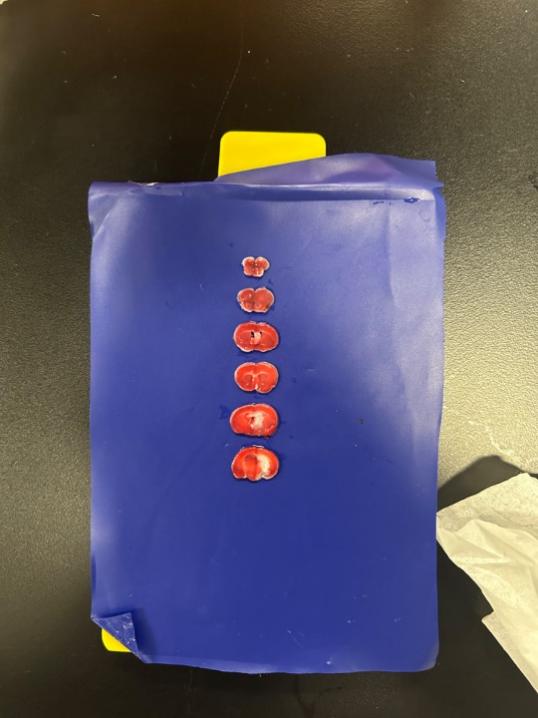


(3)
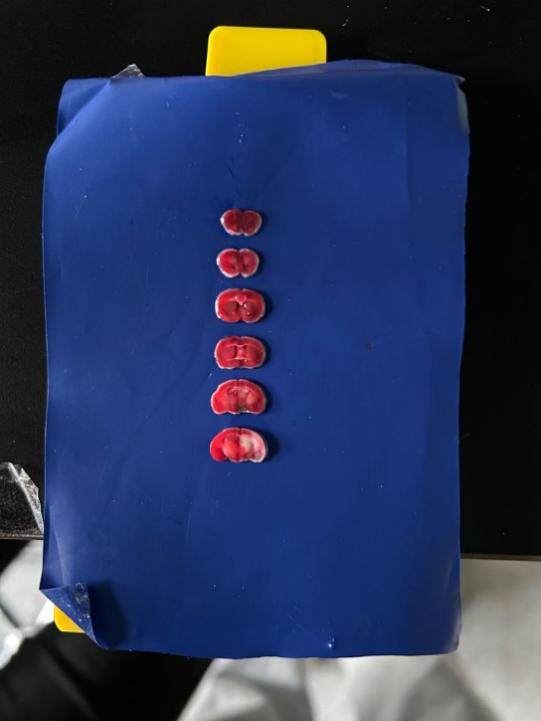

Supplement: Supplementary file 1 — Supplementary Material 1. [file 13041_2024_1135_MOESM1_ESM.docx]

**The sham group:**

(1)
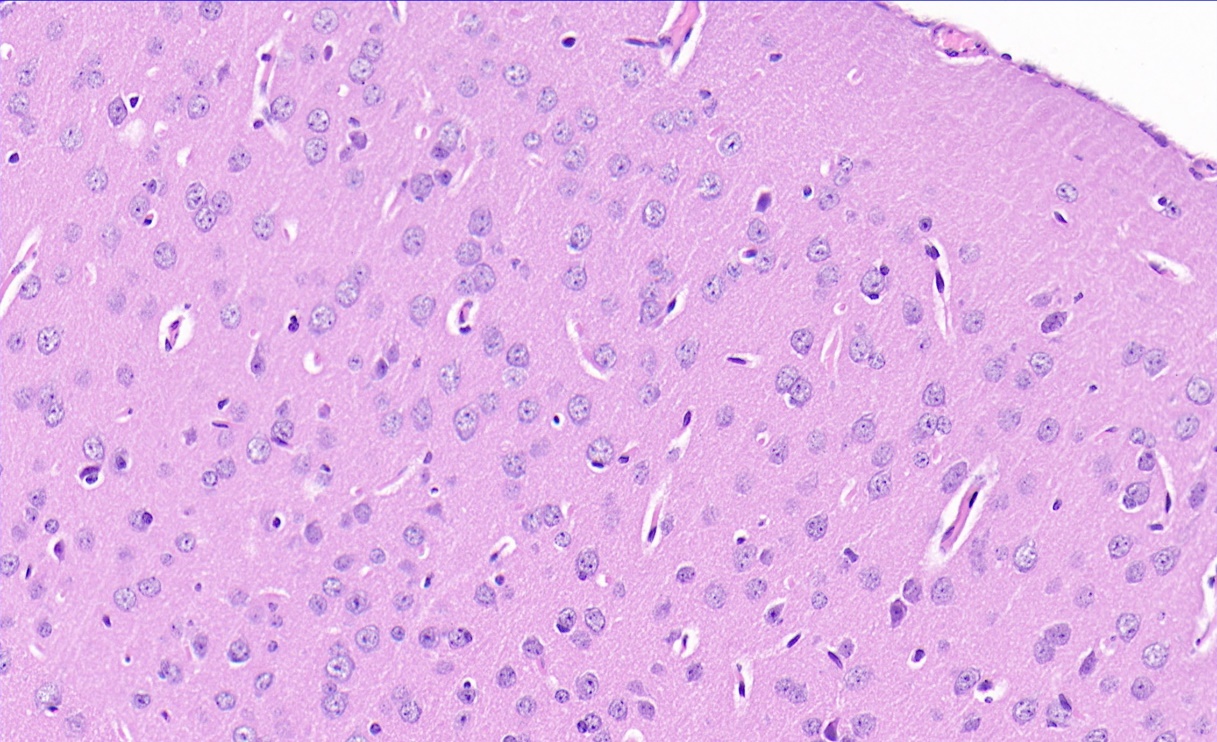


(2)
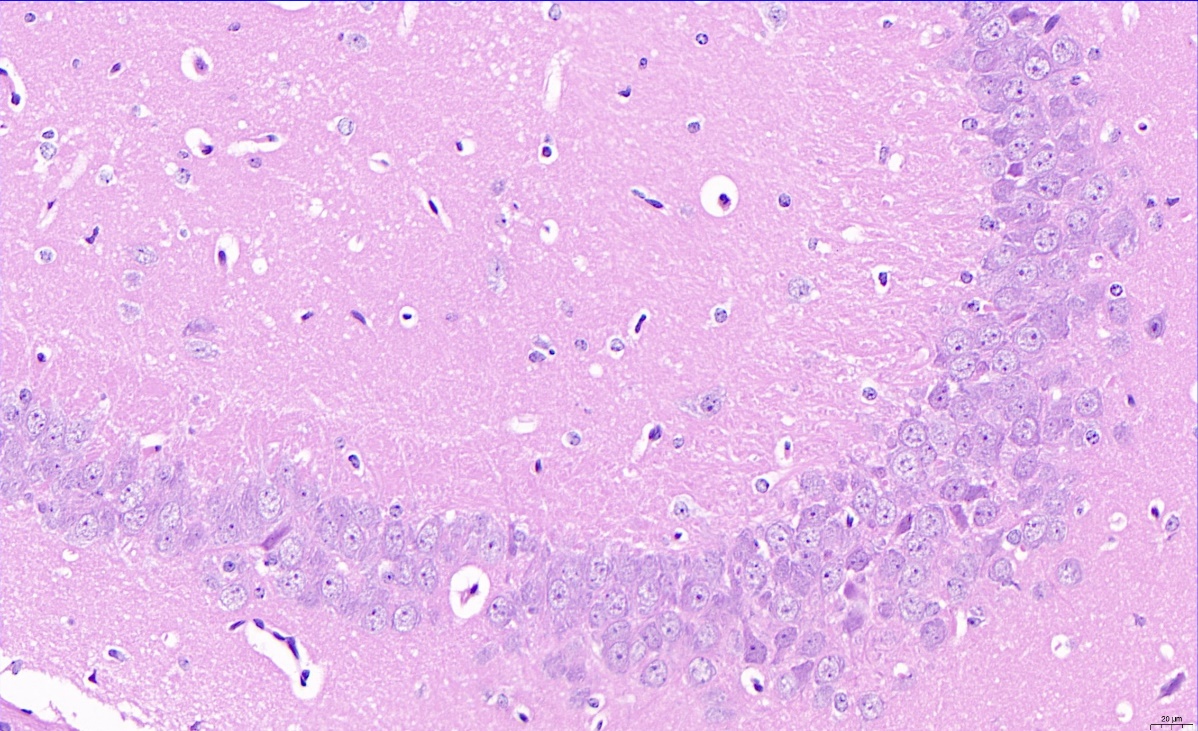


(3)
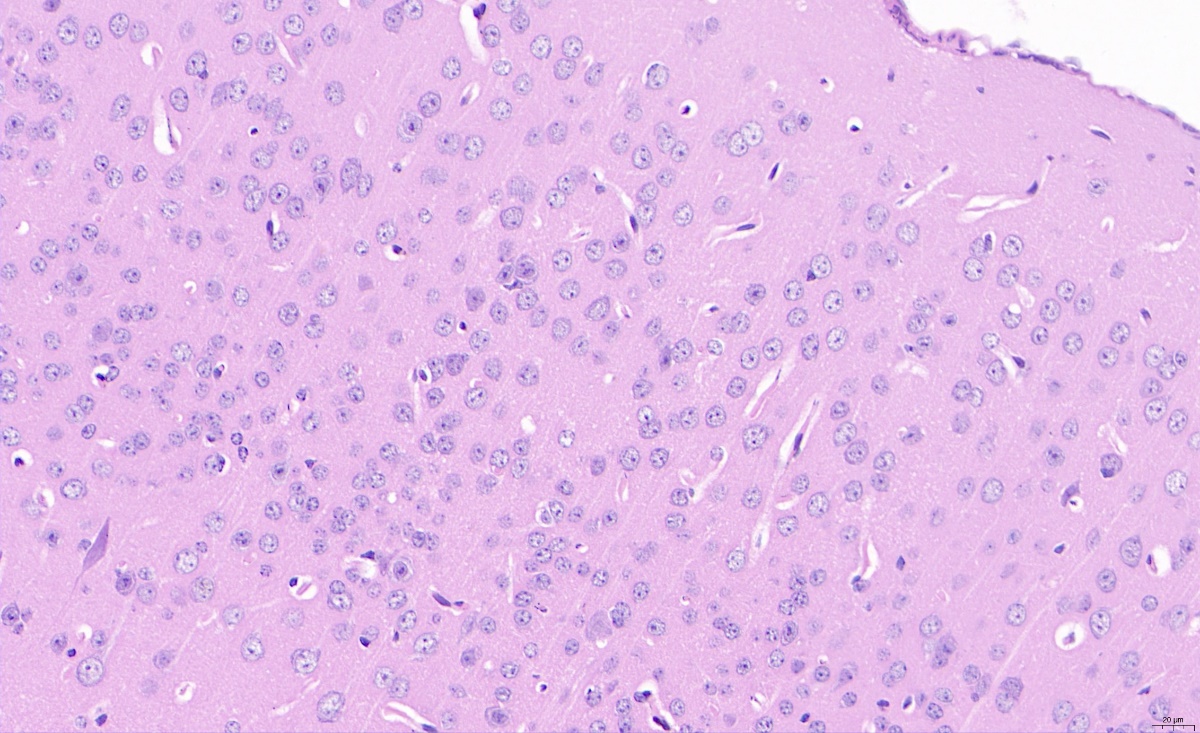


(4)
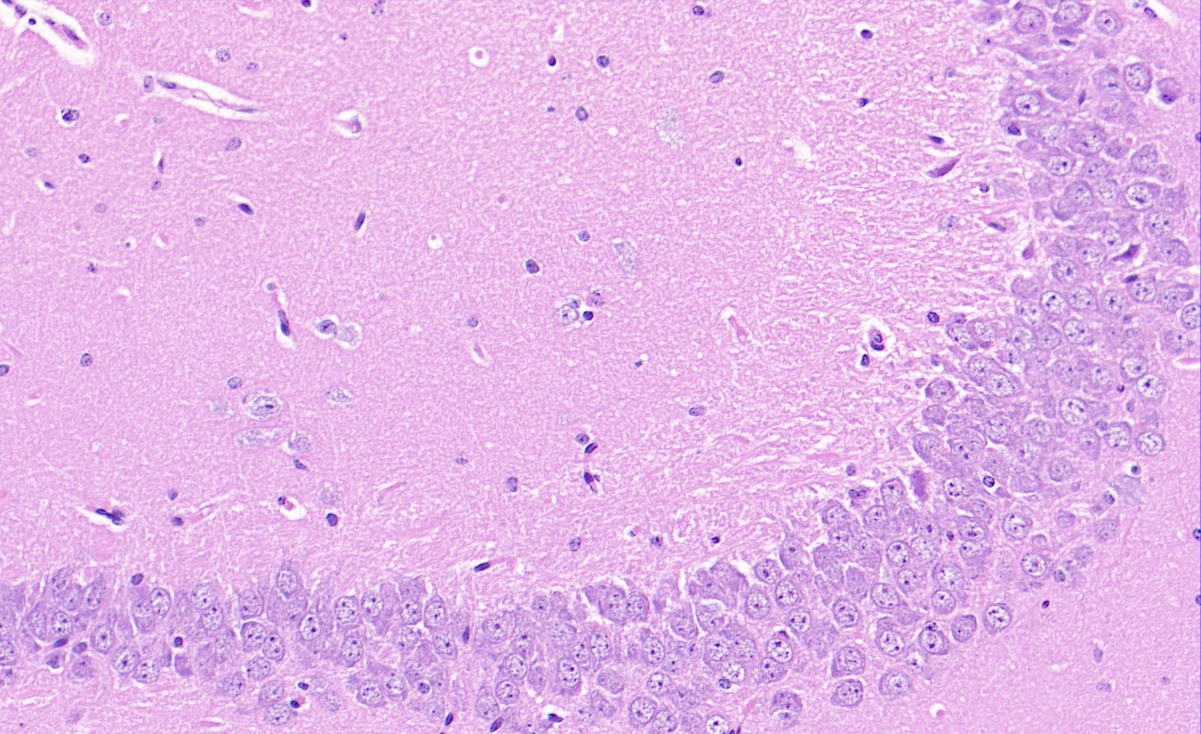


**The I/R group:**

(1)
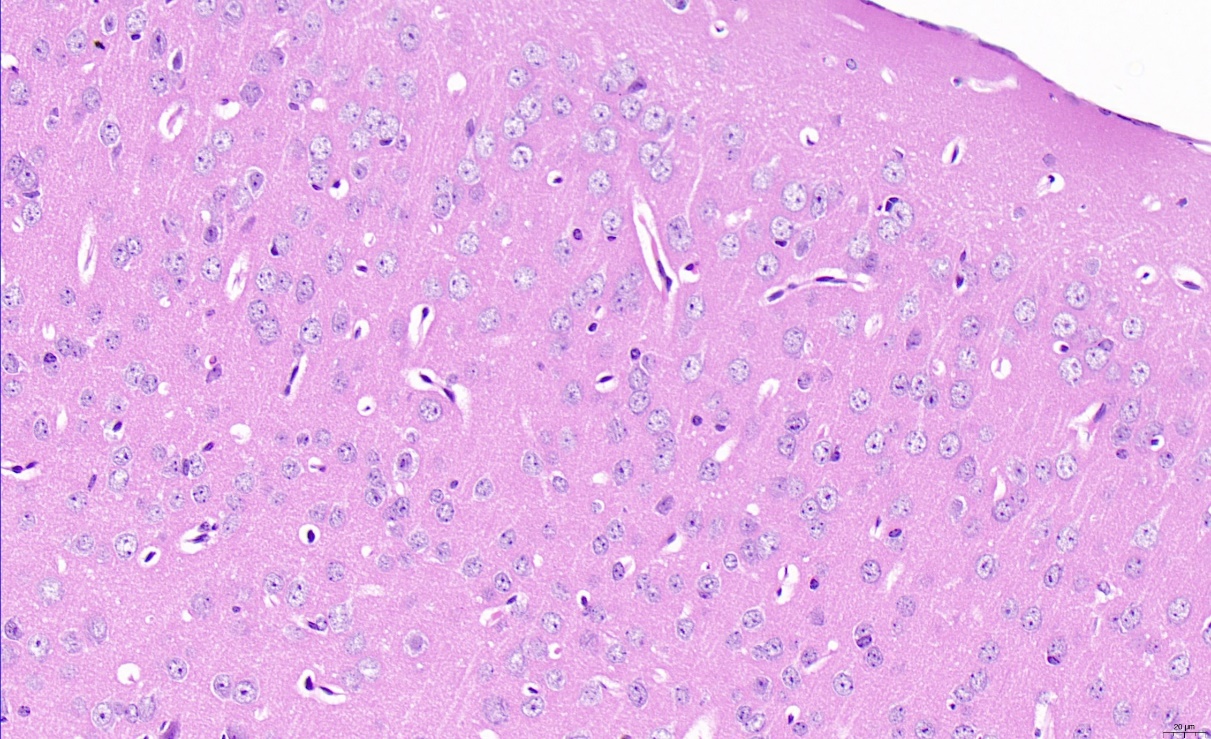
(2)
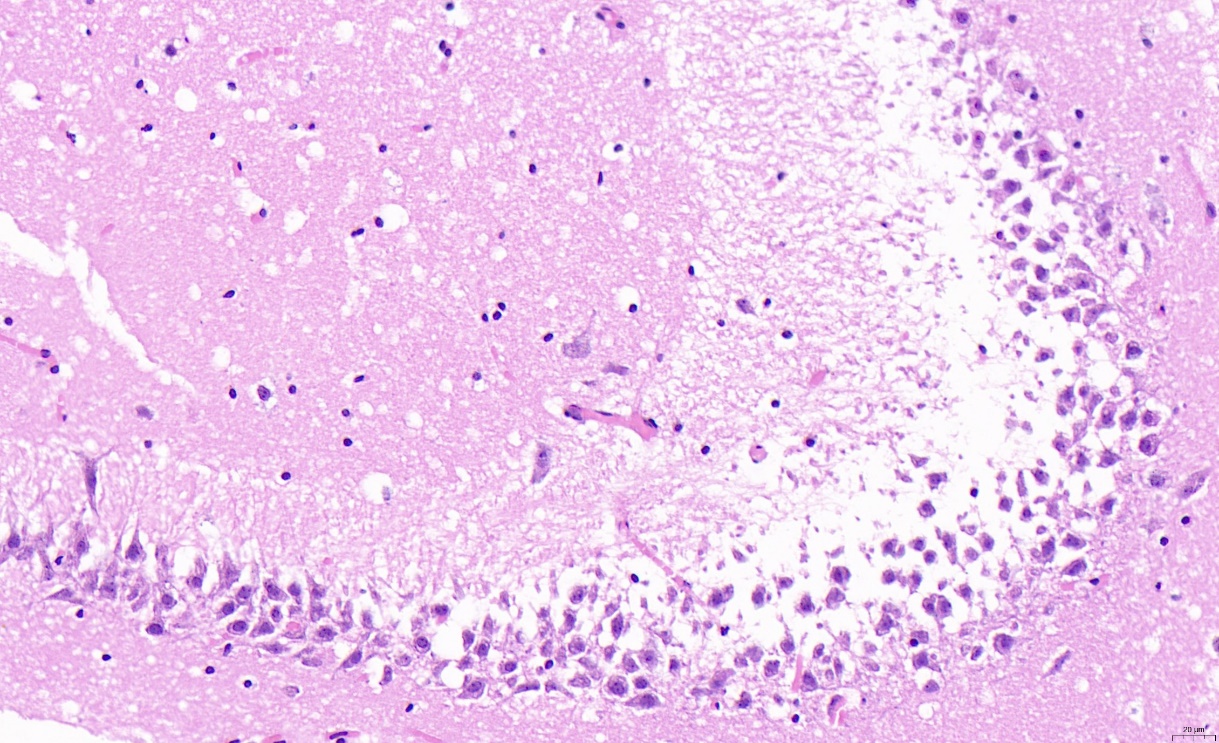


(3)
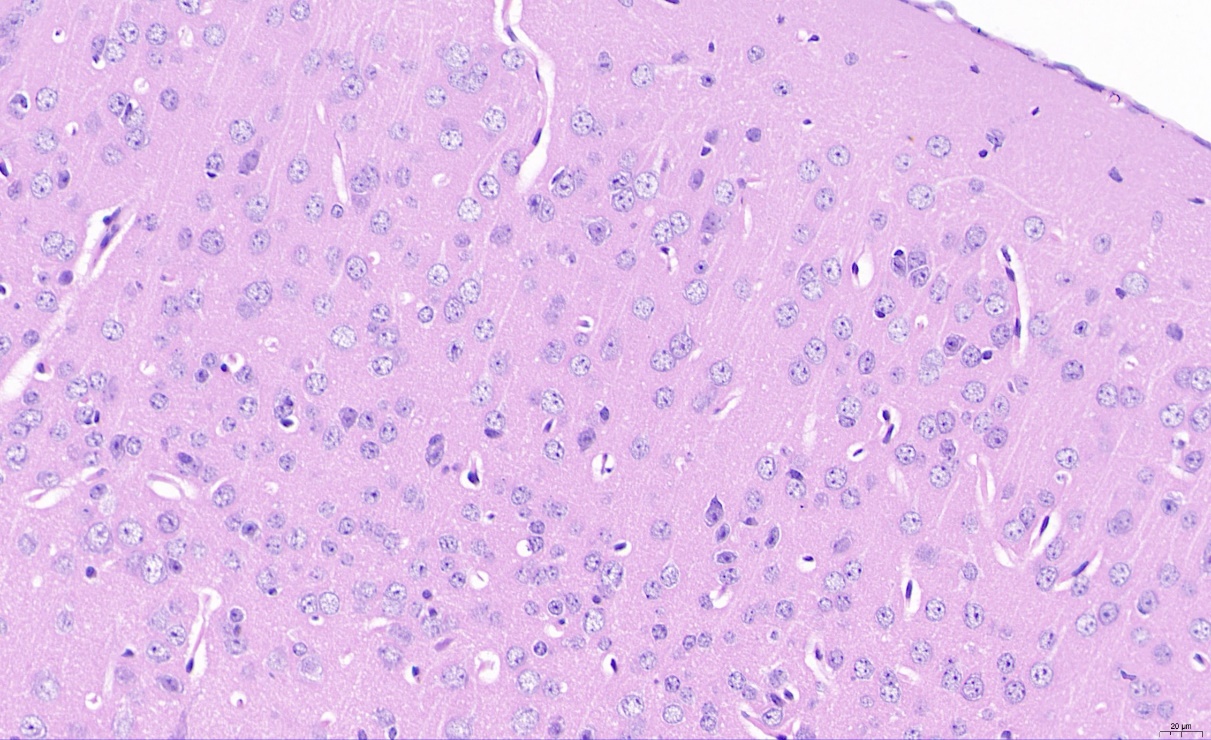


(4)
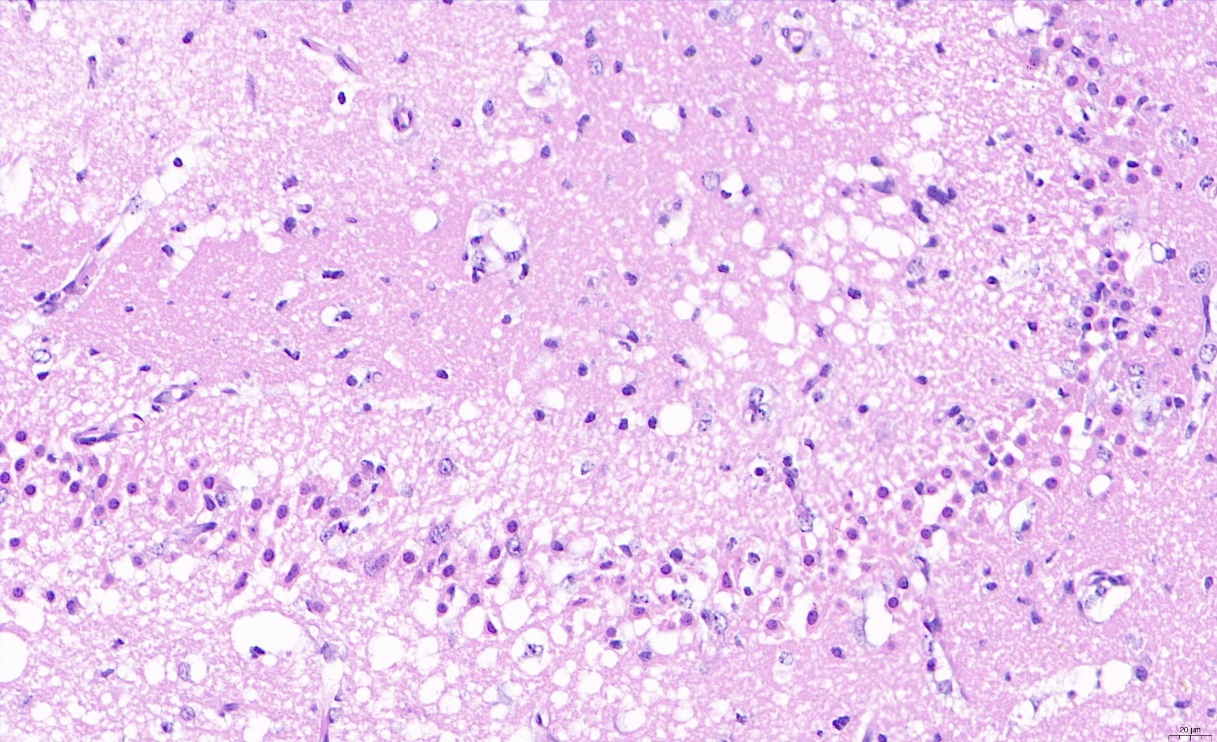


**The EA group:**

(1)
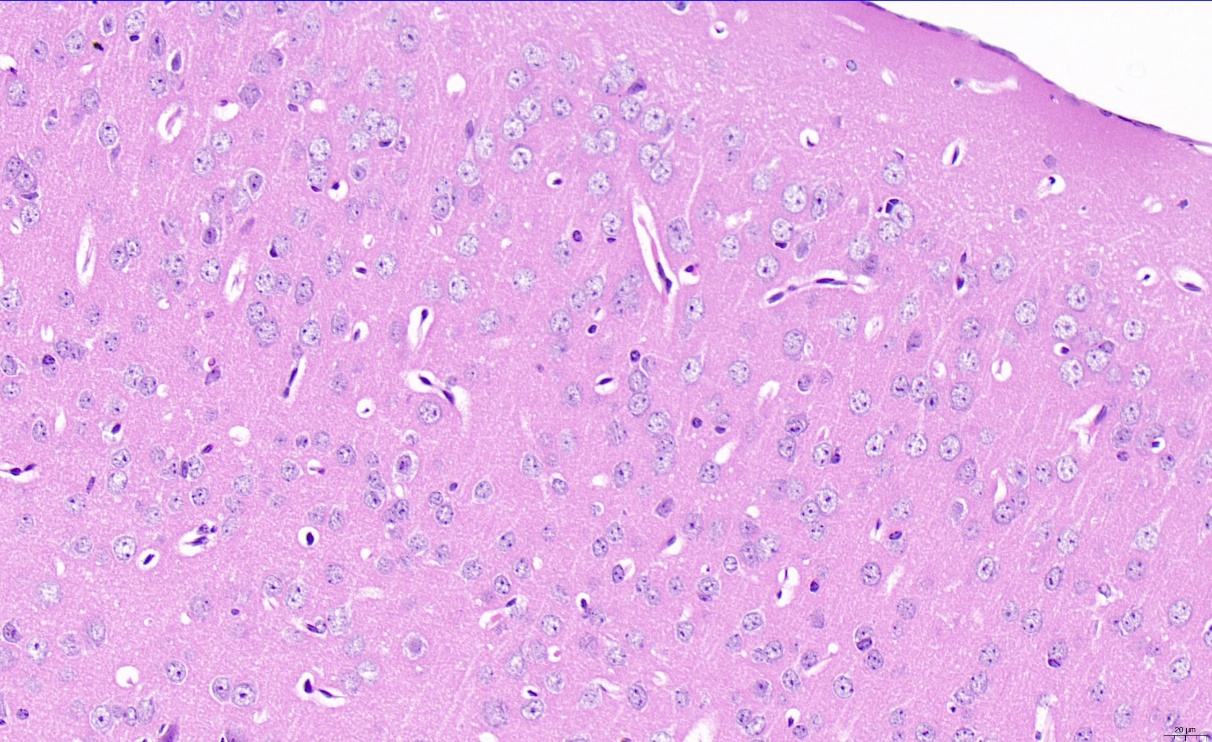


(2)
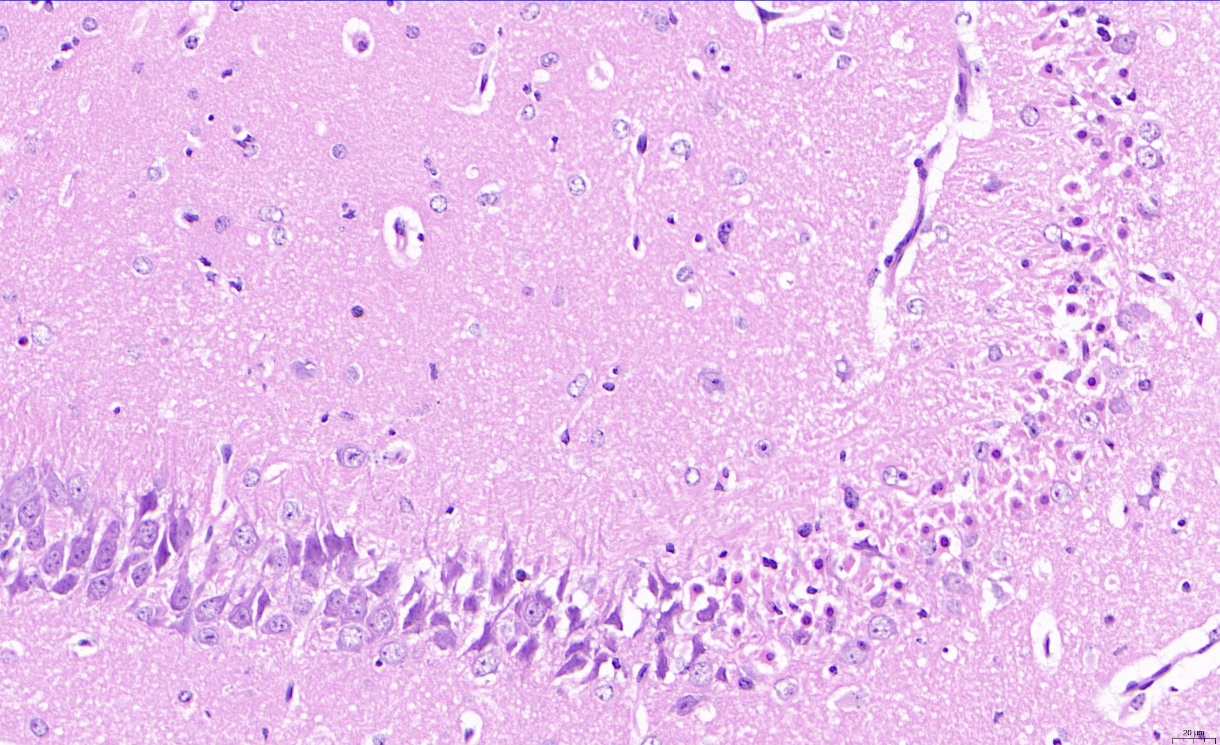


(3)
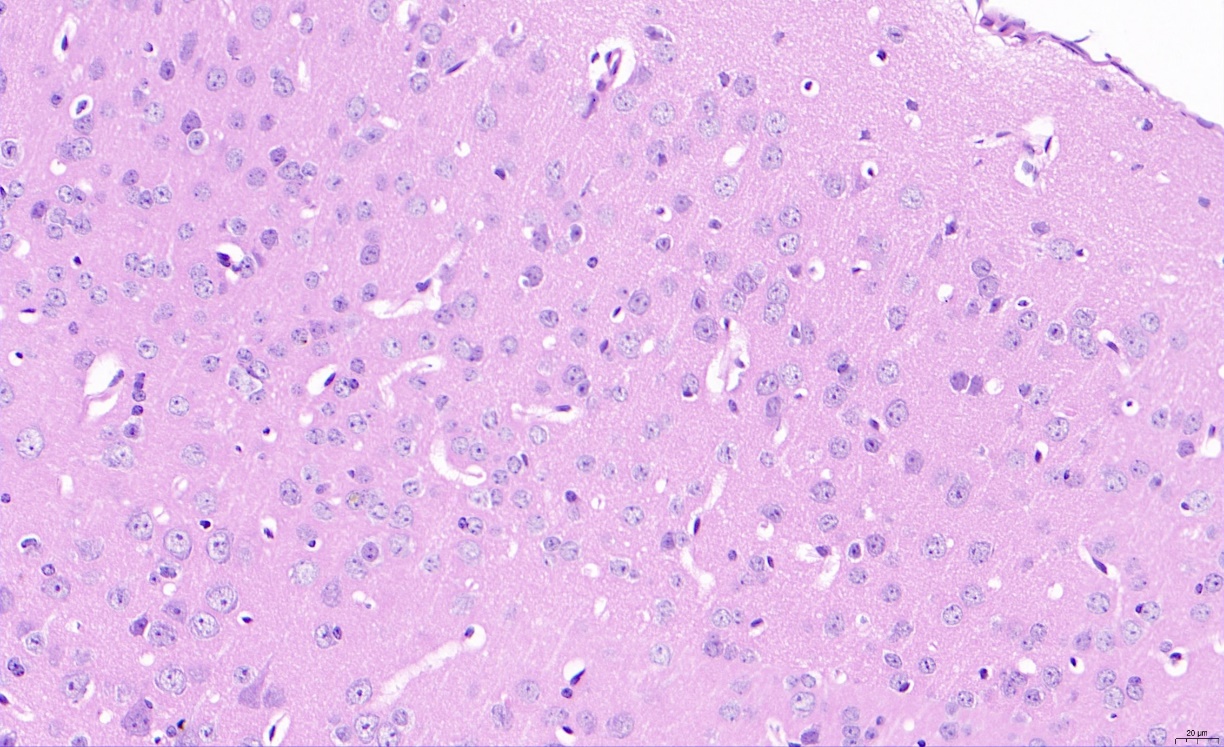


(4)
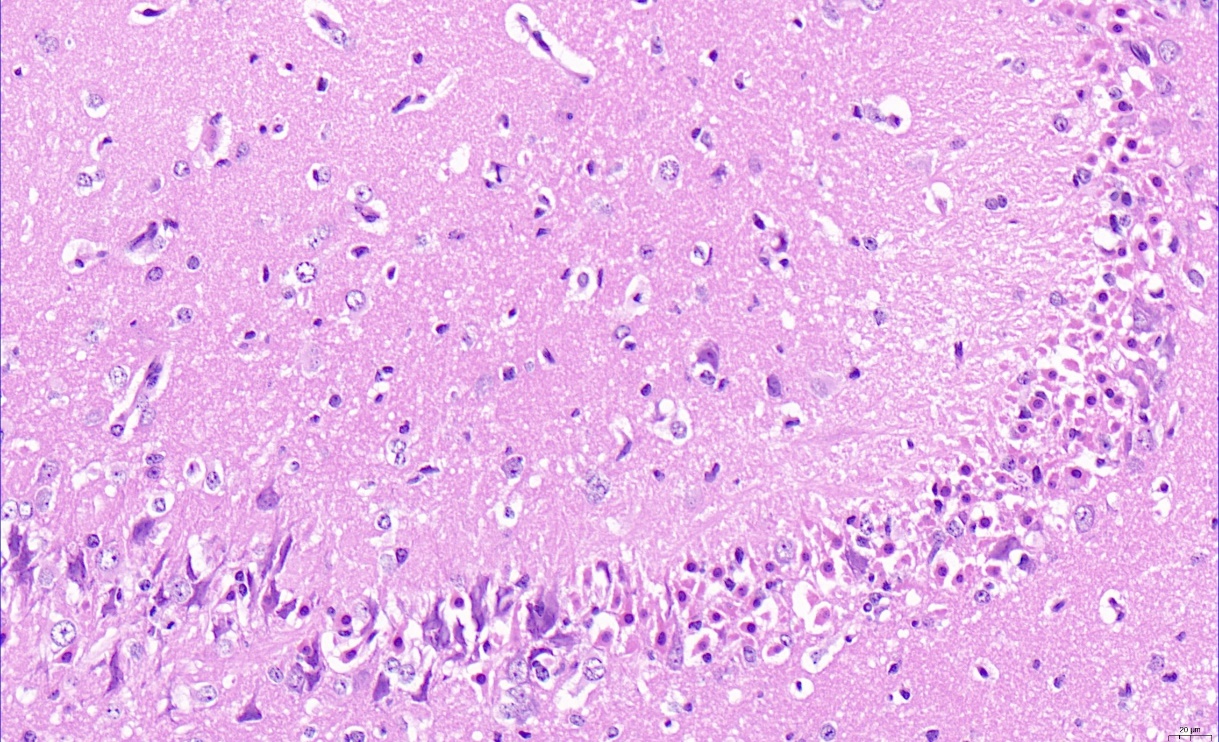

Supplement: Supplementary file 3 — Supplementary Material 3. [file 13041_2024_1135_MOESM3_ESM.docx]

**The sham group:**

(1)
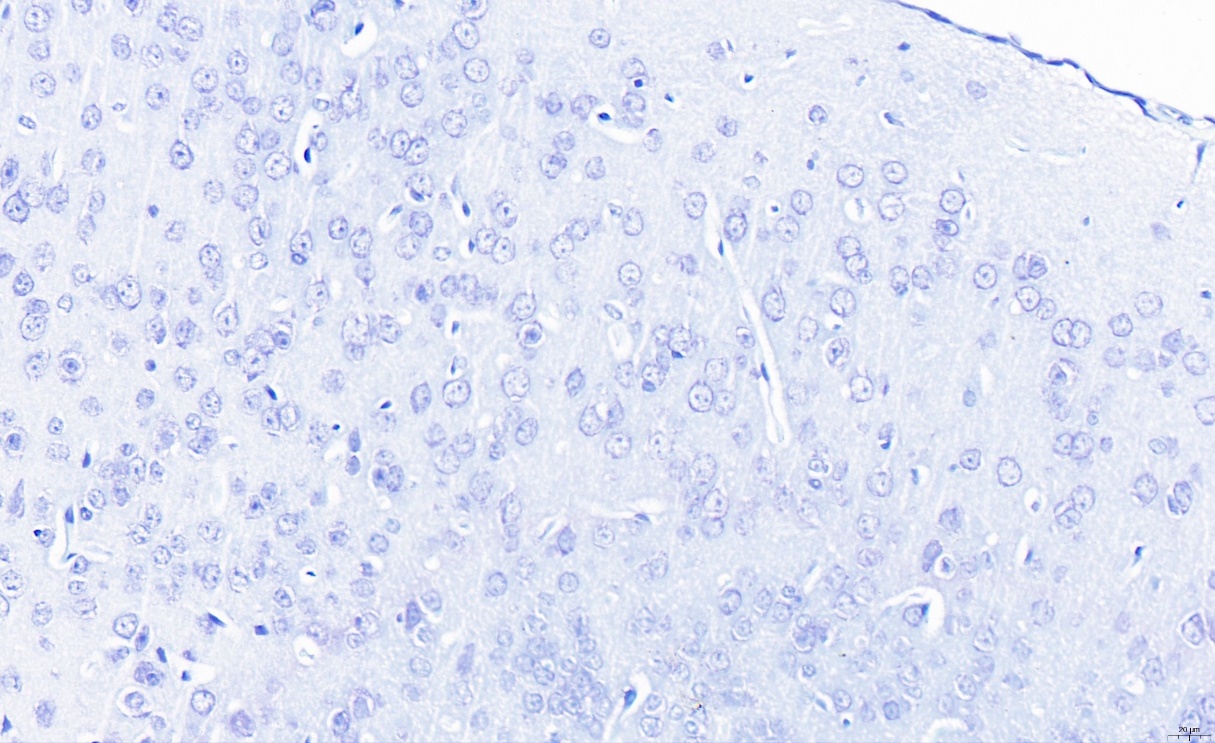


(2)
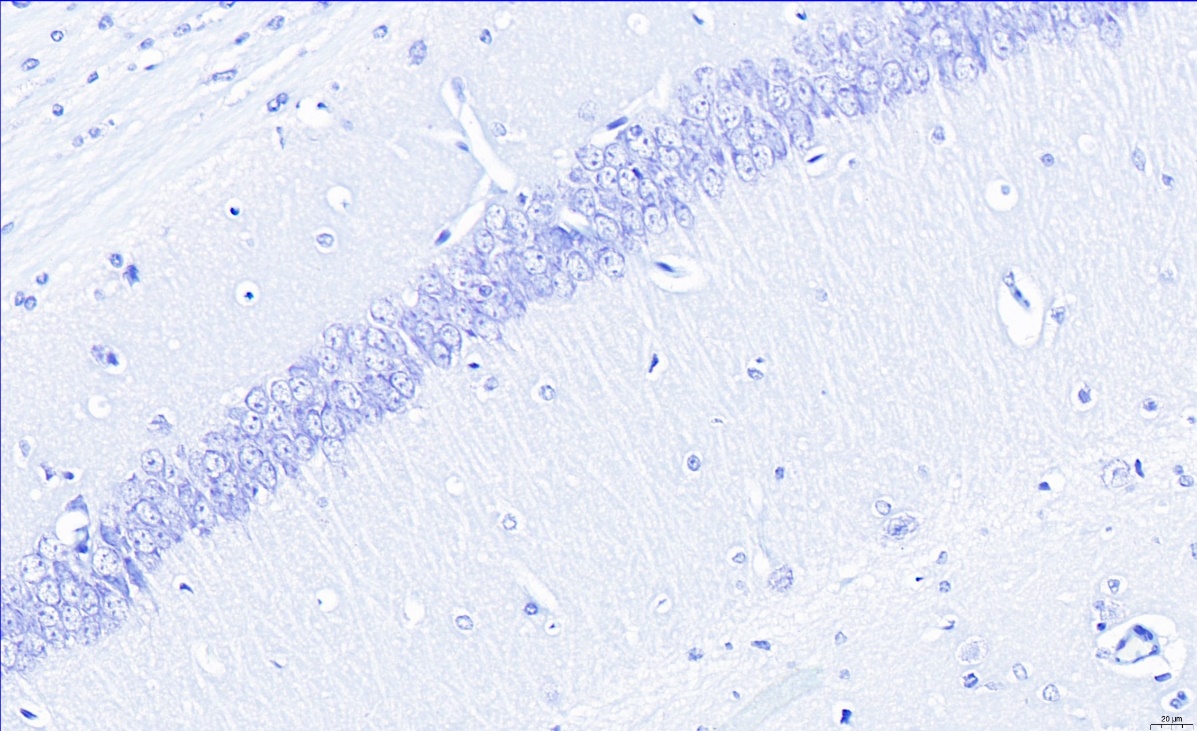


(3)
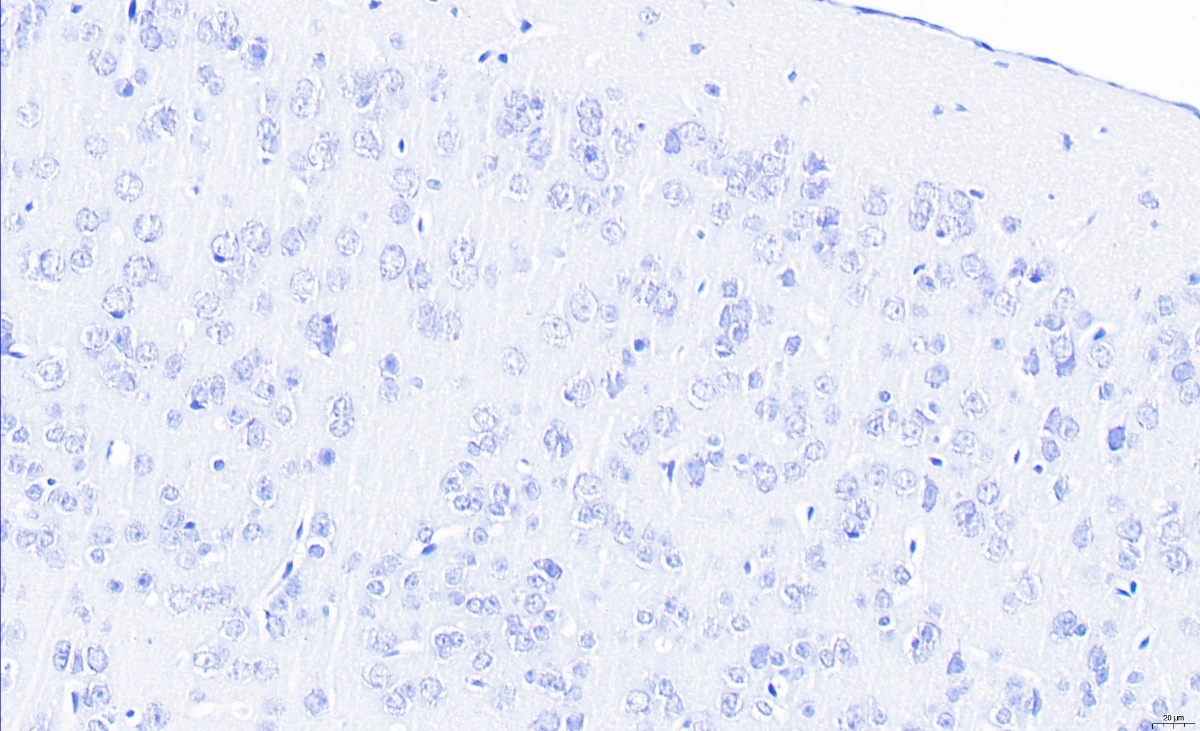


(4)
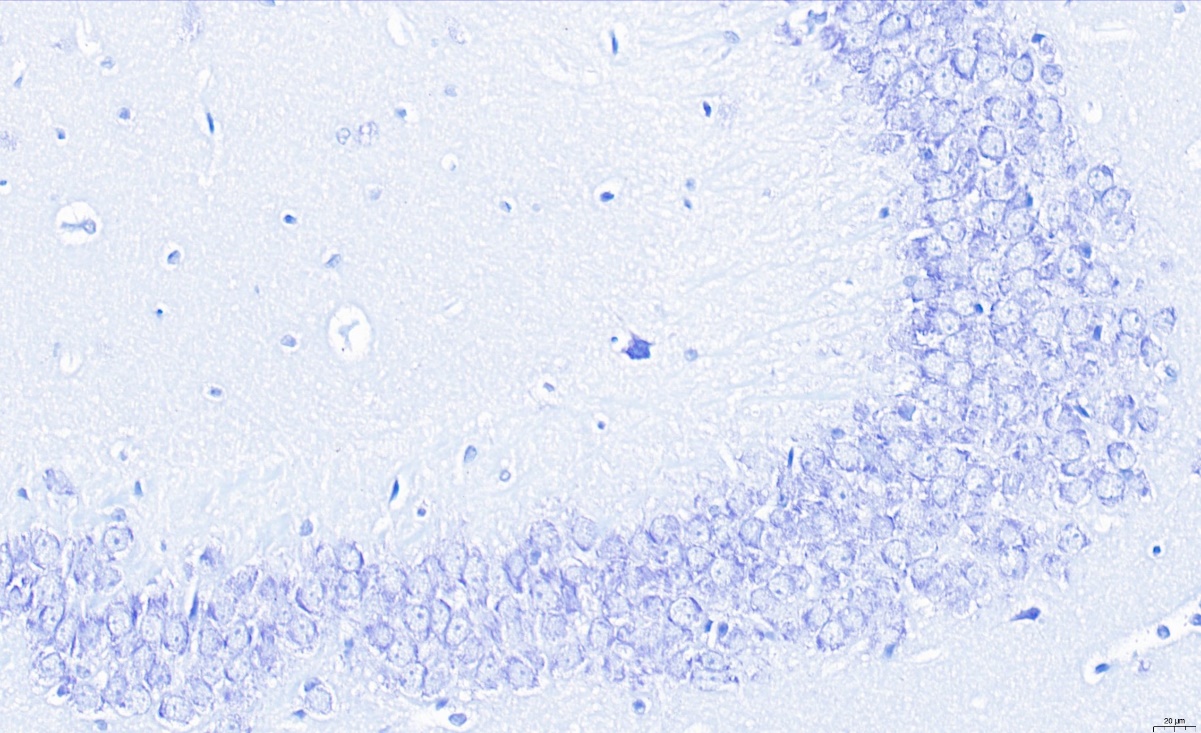


**The I/R group:**

(1)
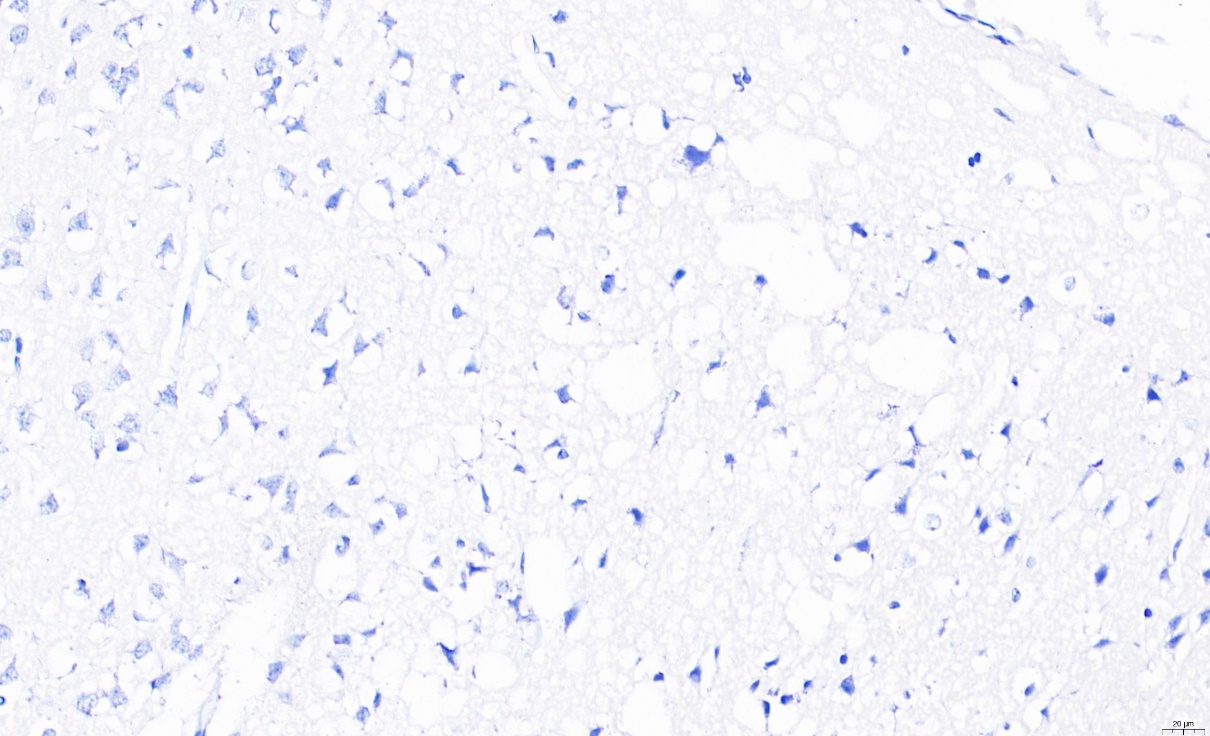
(2)
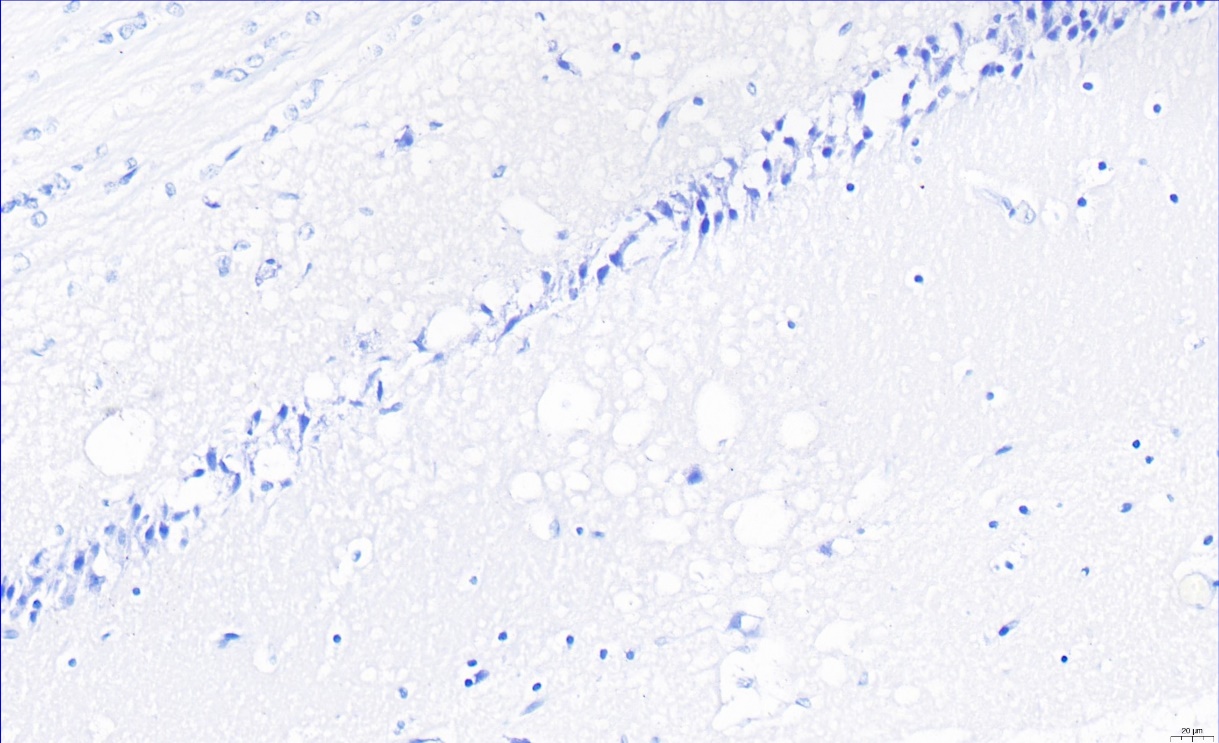


(3)
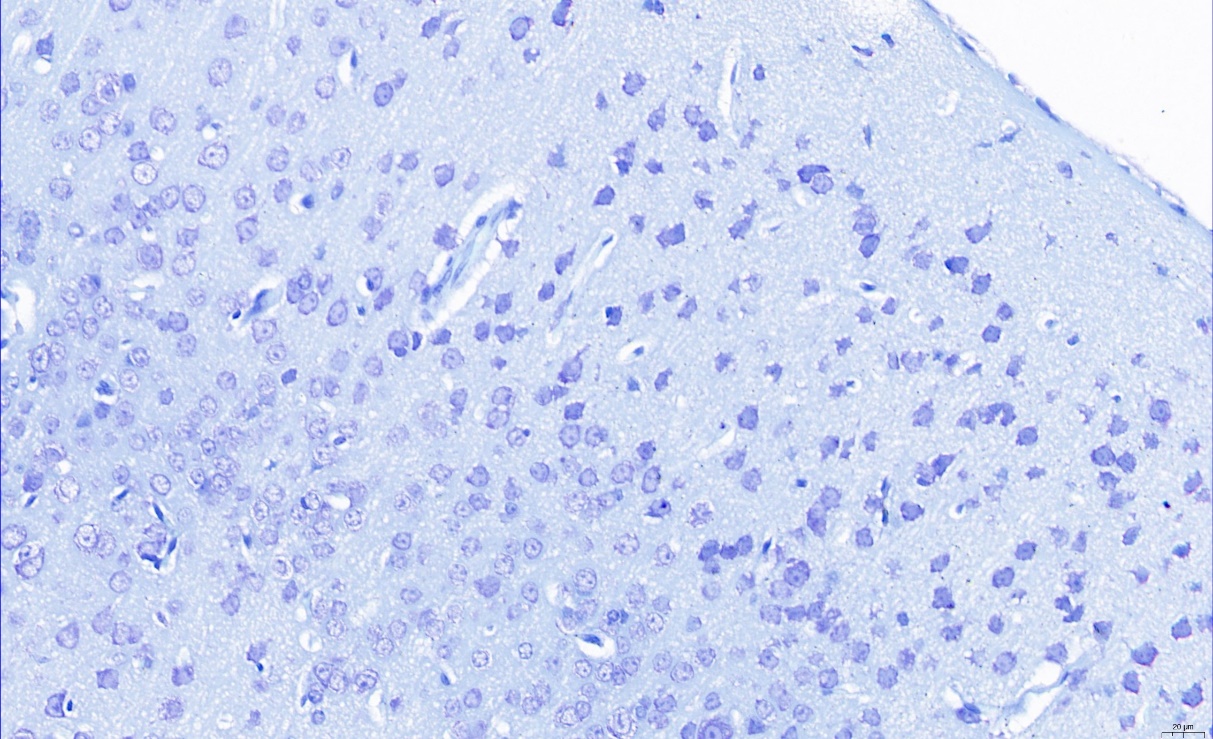


(4)
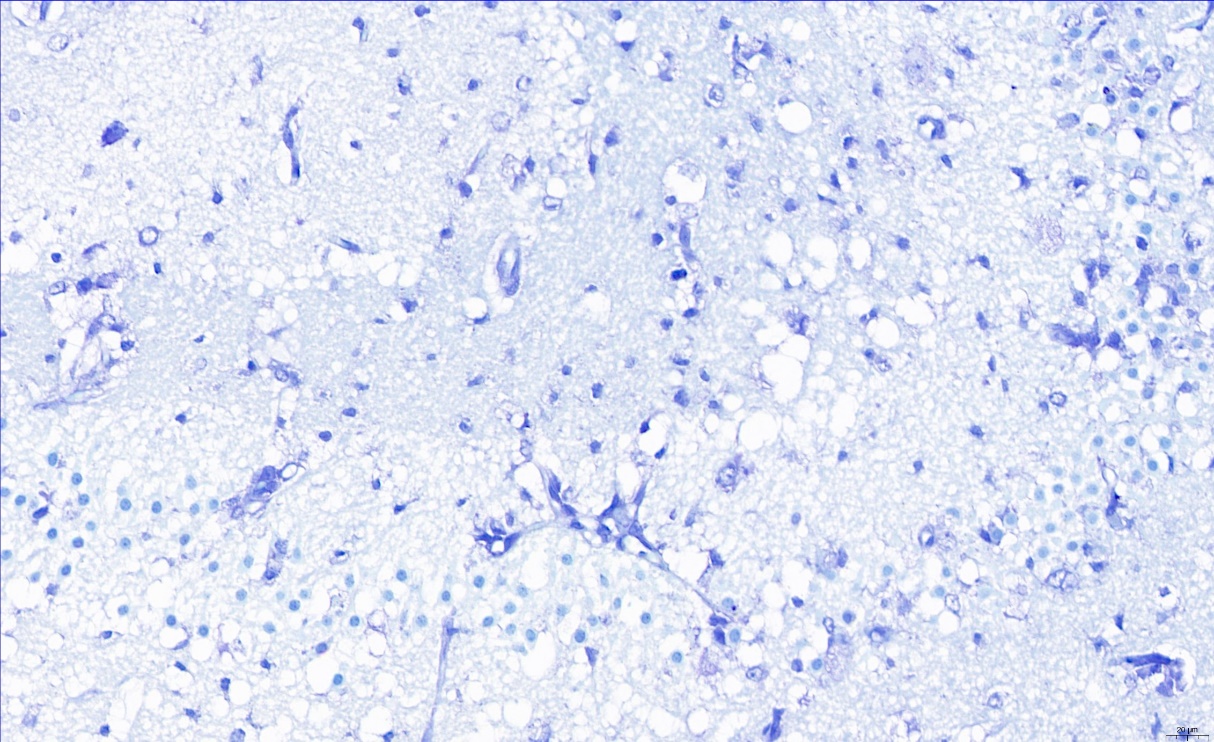


**The EA group:**

(1)
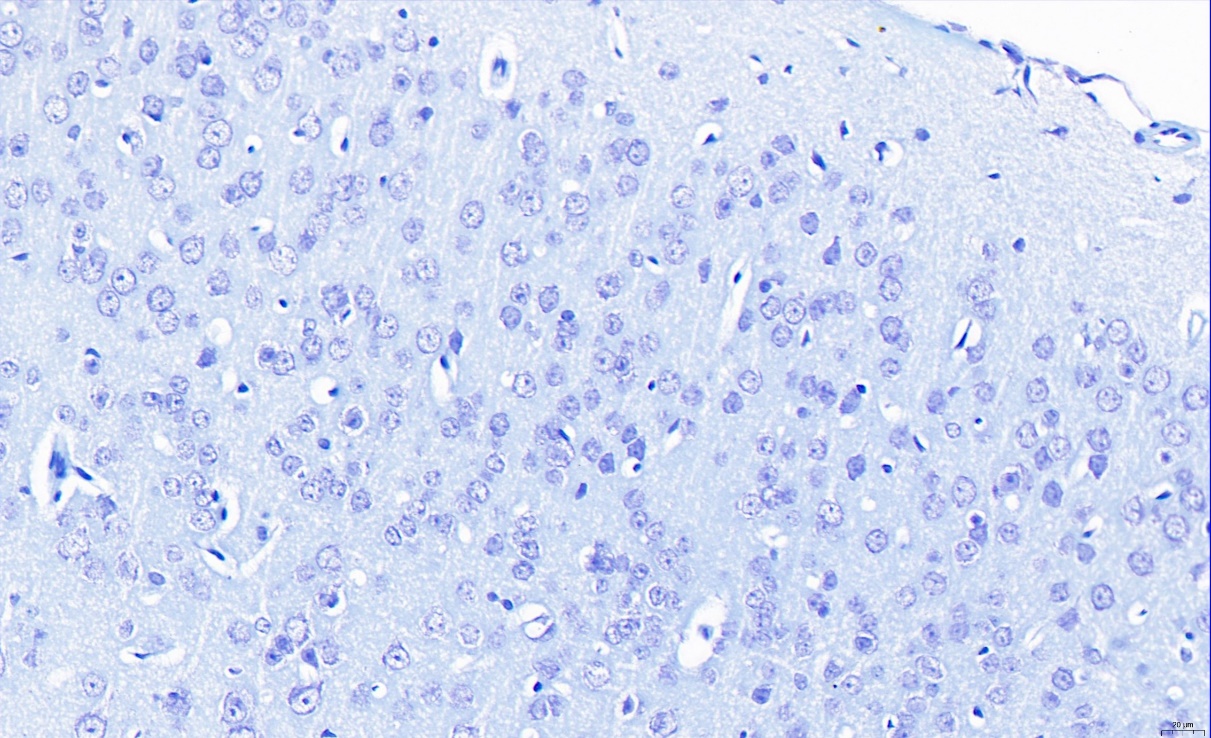


(2)
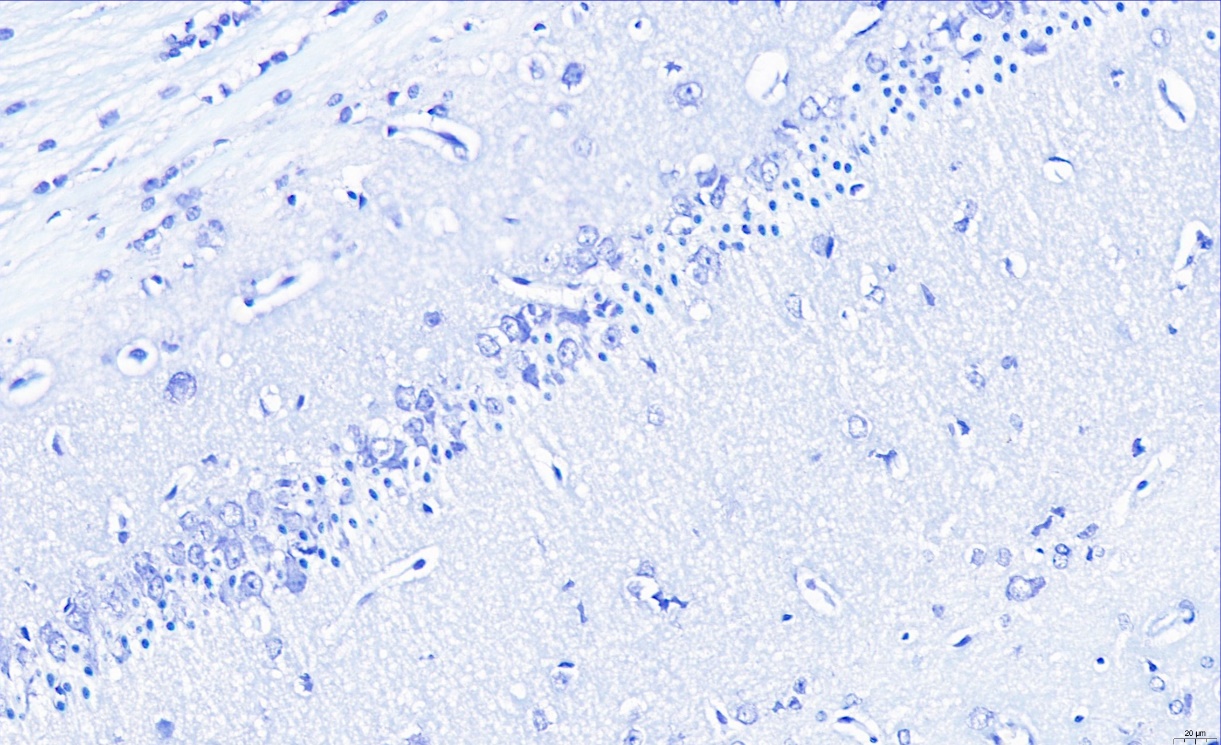


(3)
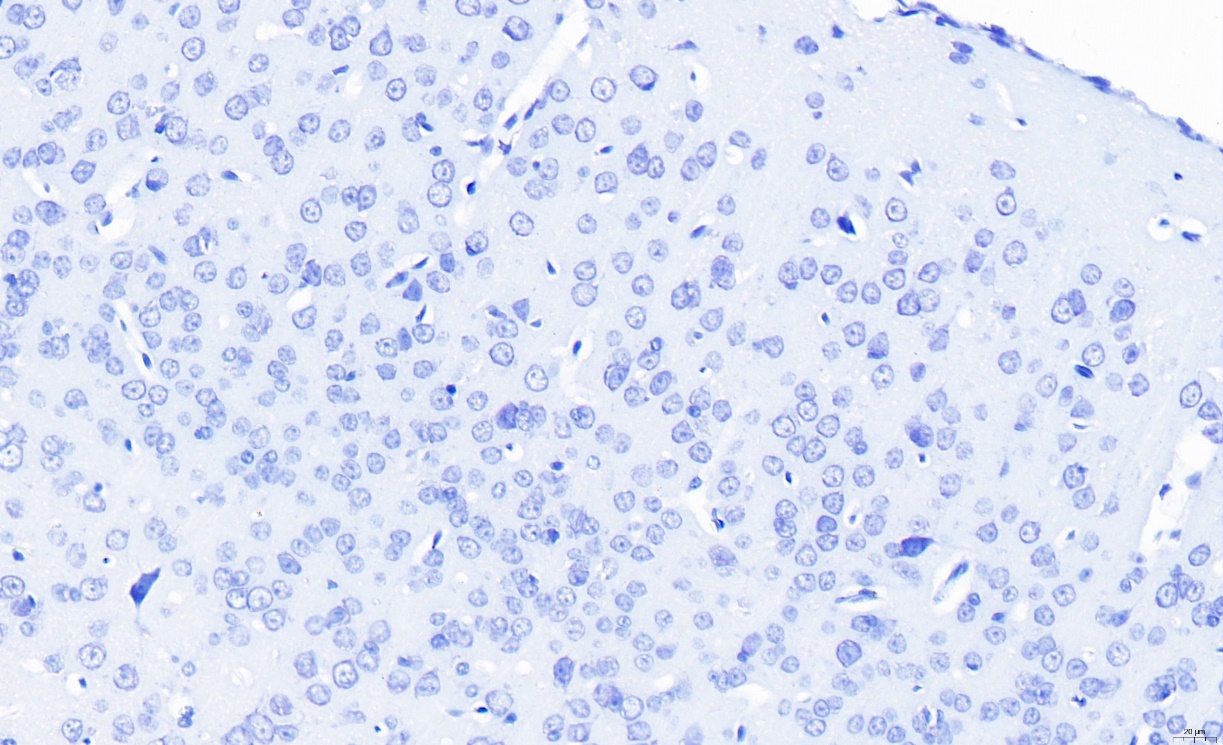


(4)
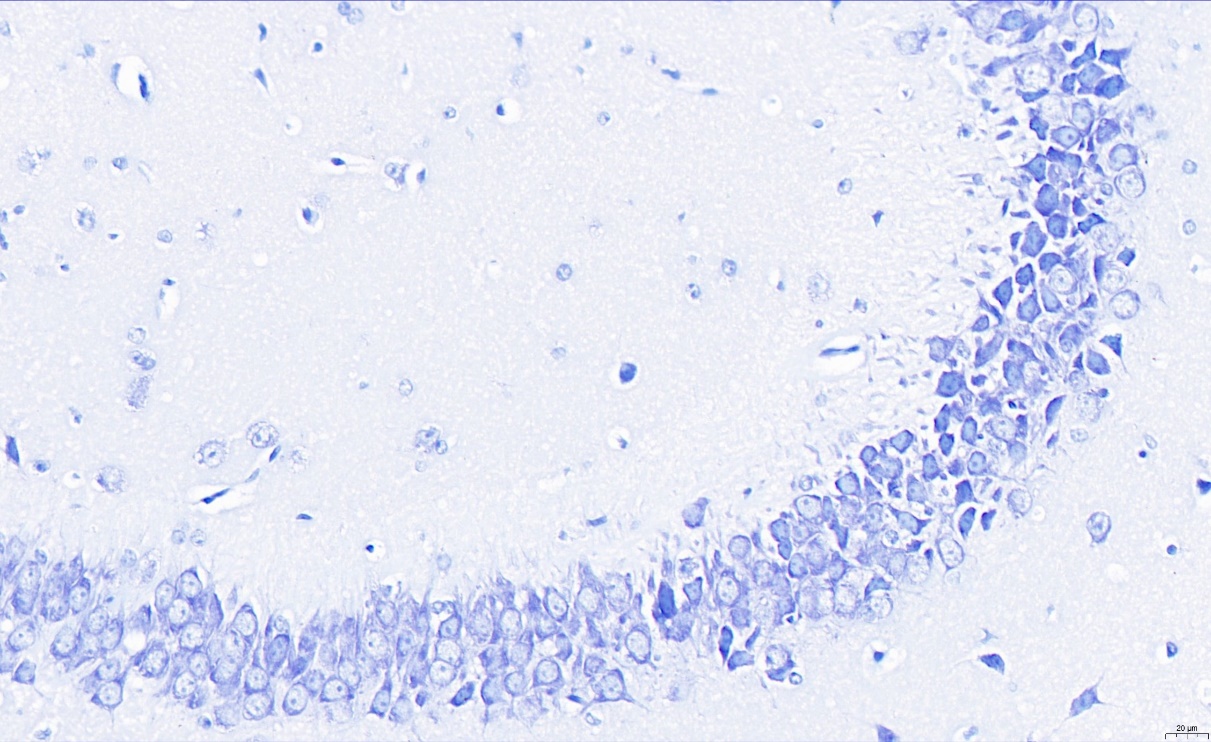

Supplement: Supplementary file 4 — Supplementary Material 4. [file 13041_2024_1135_MOESM4_ESM.docx]

**NEUN:**

(1)
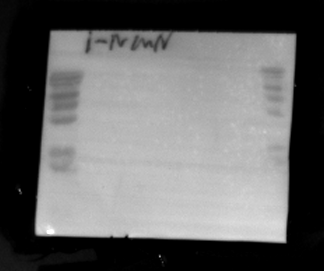

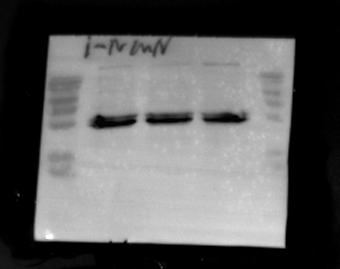

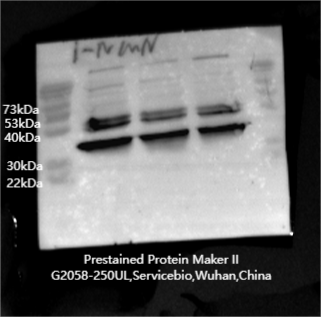


(2)
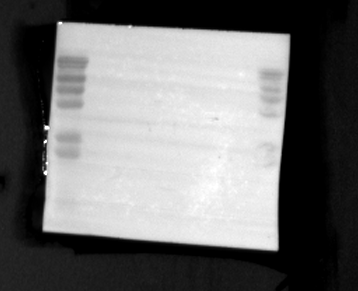

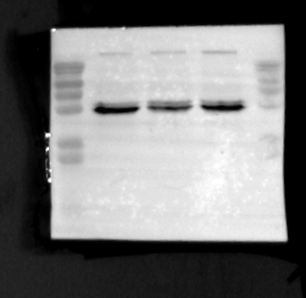

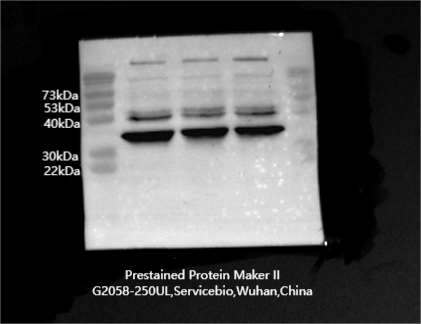


(3)
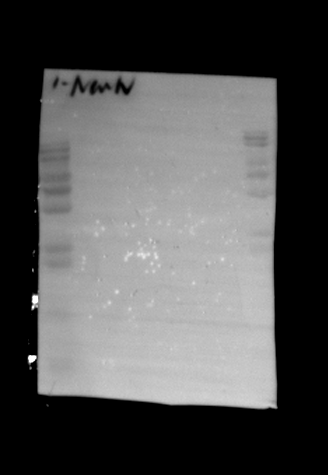

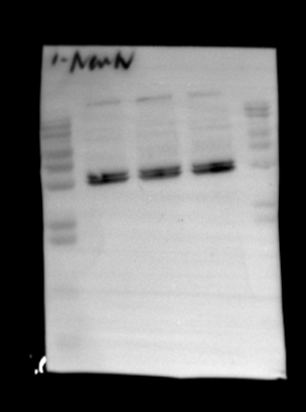

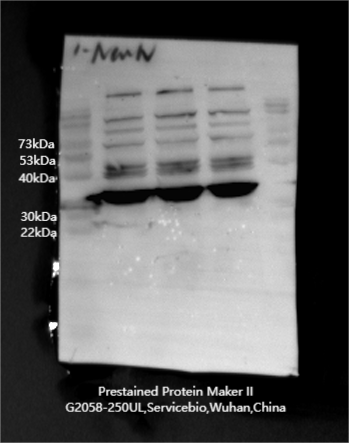


(4)
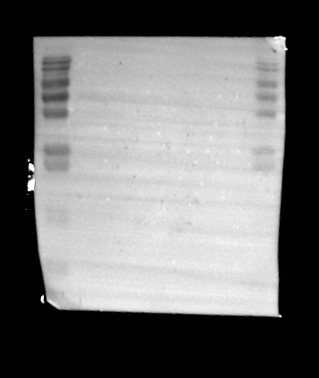

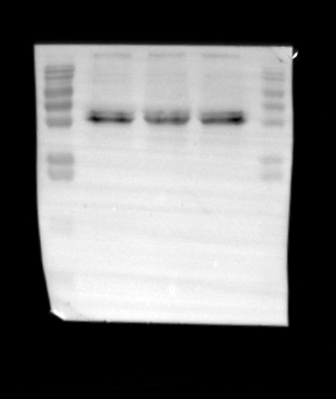

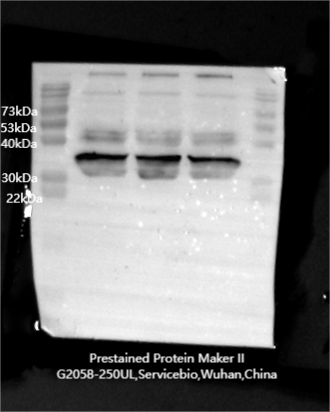


(5)
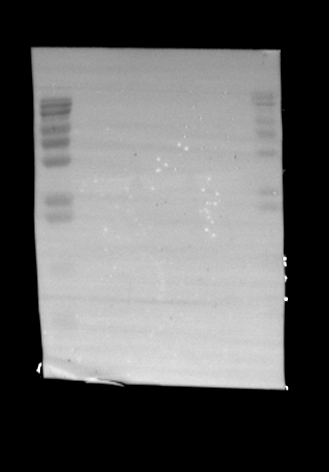

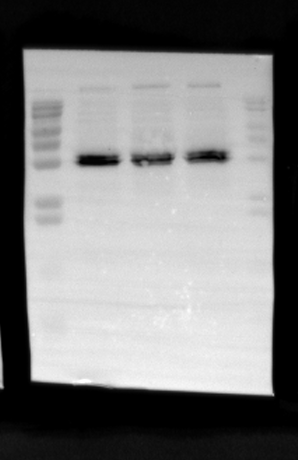

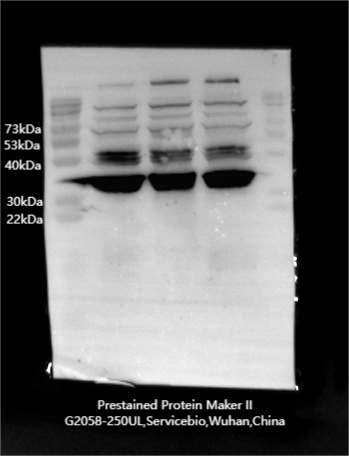


(6)
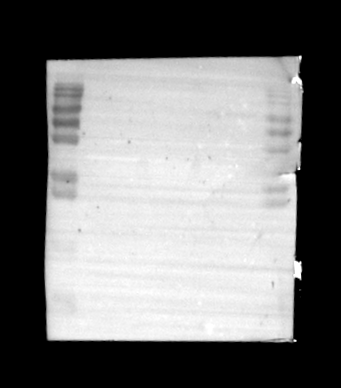

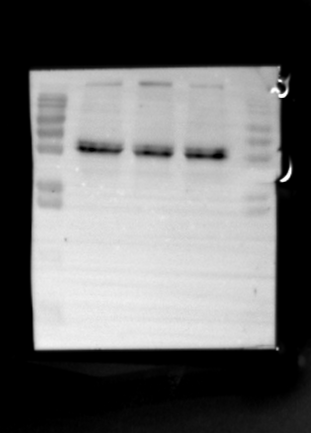

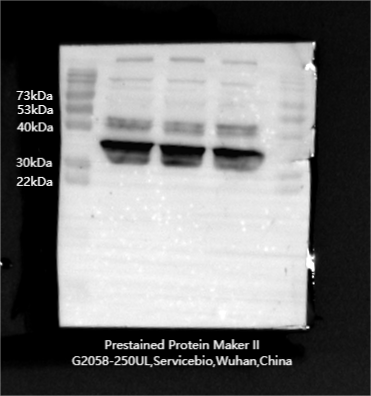


**IBA1:**

(1)
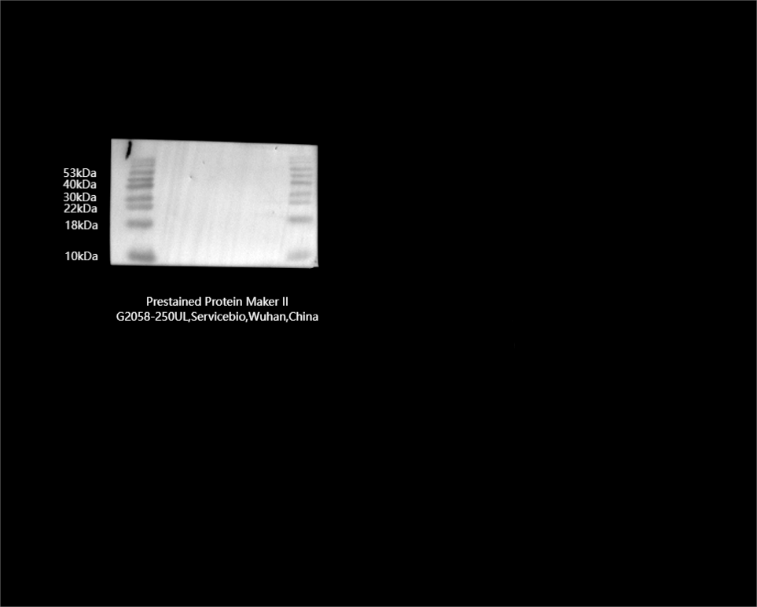

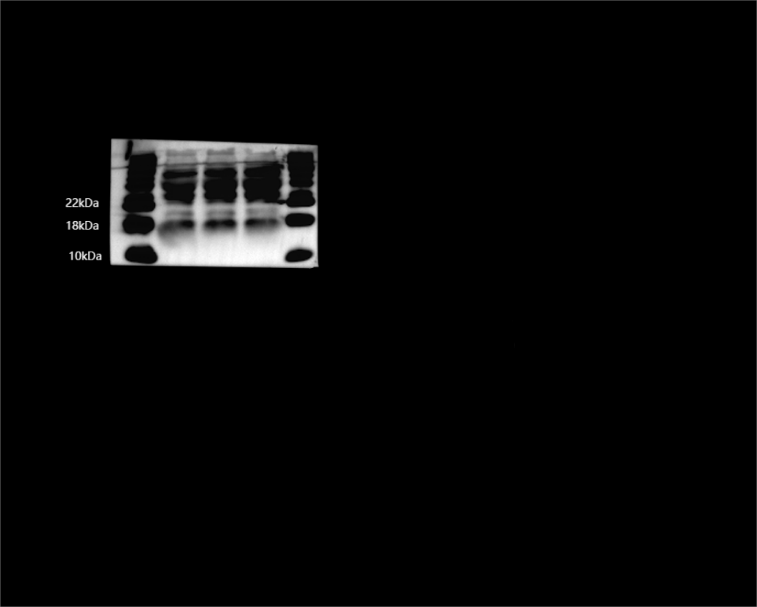

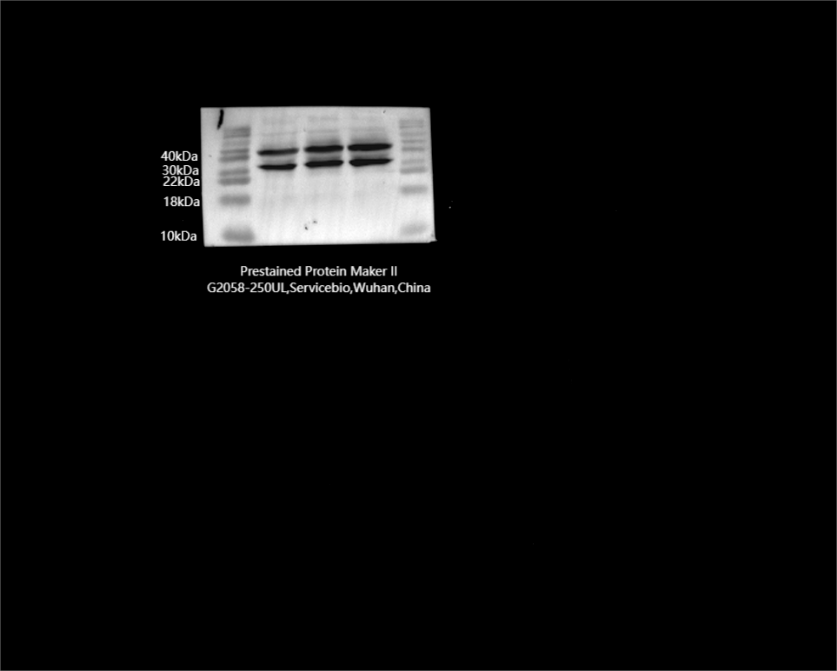


(2)
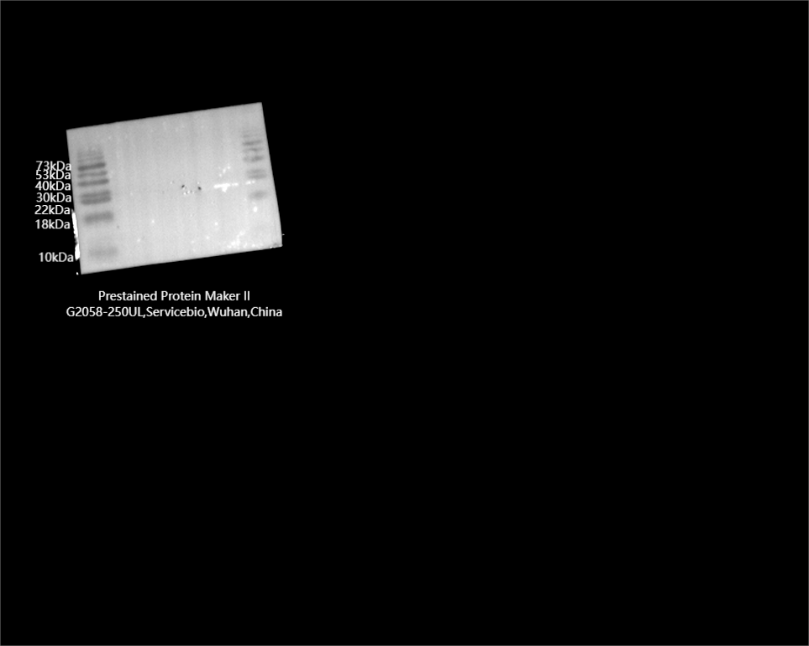

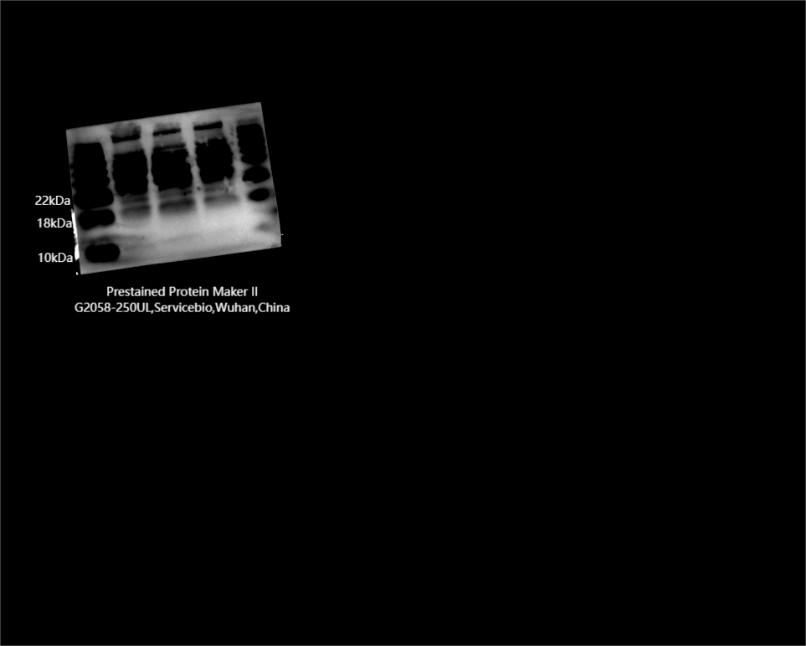

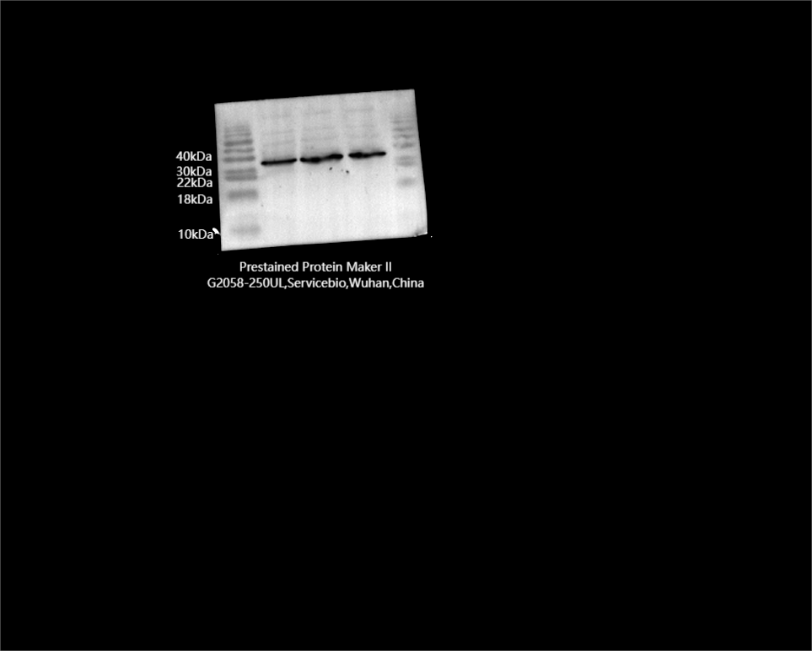


(3)
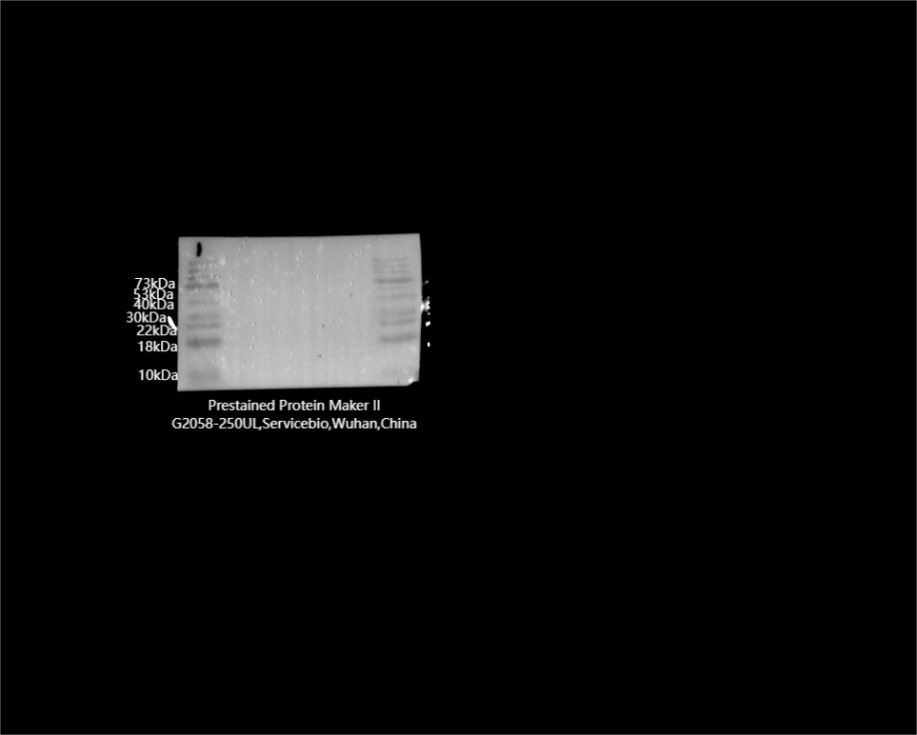

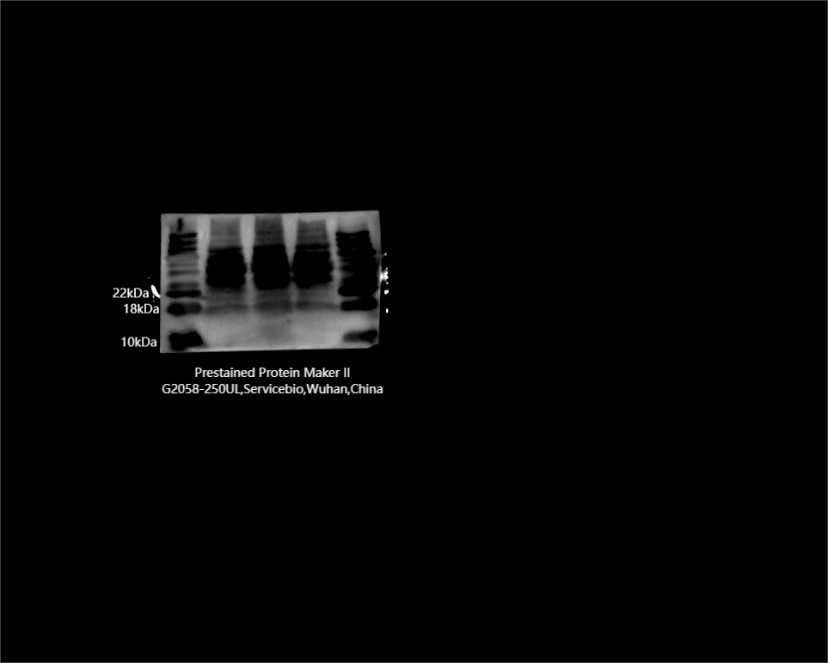

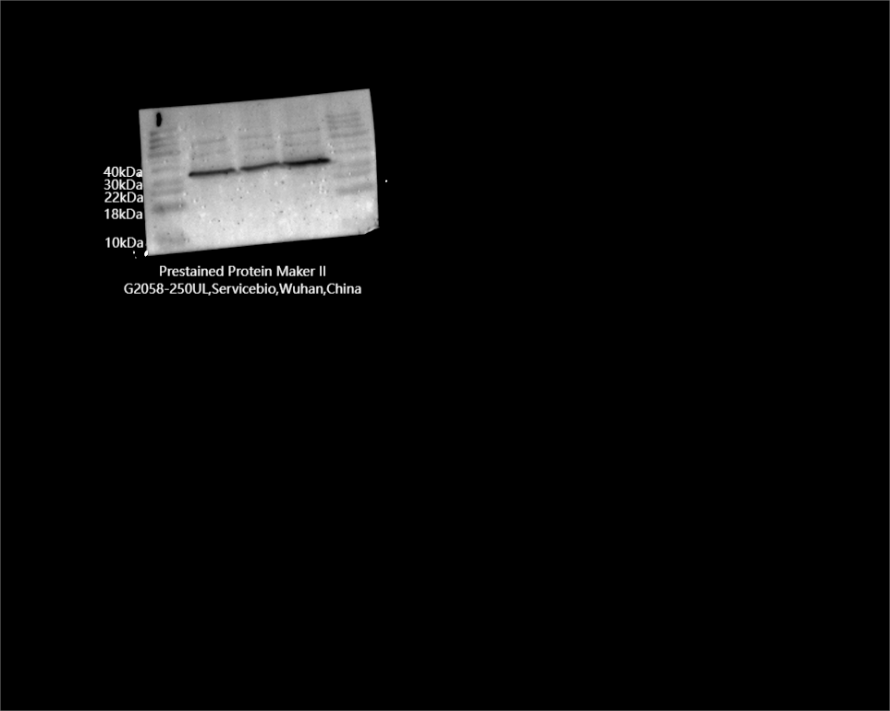


(4)
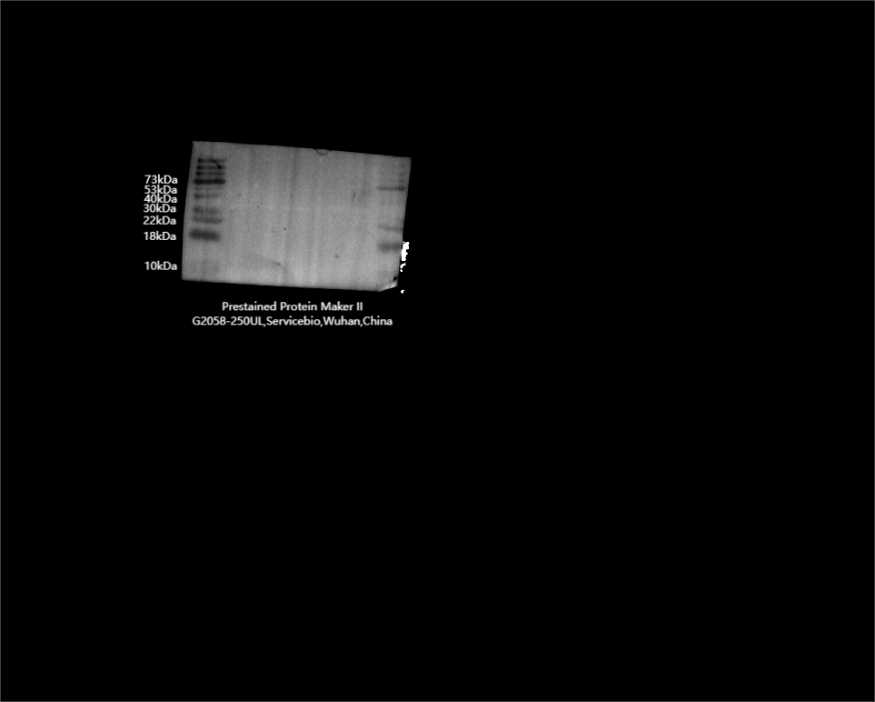

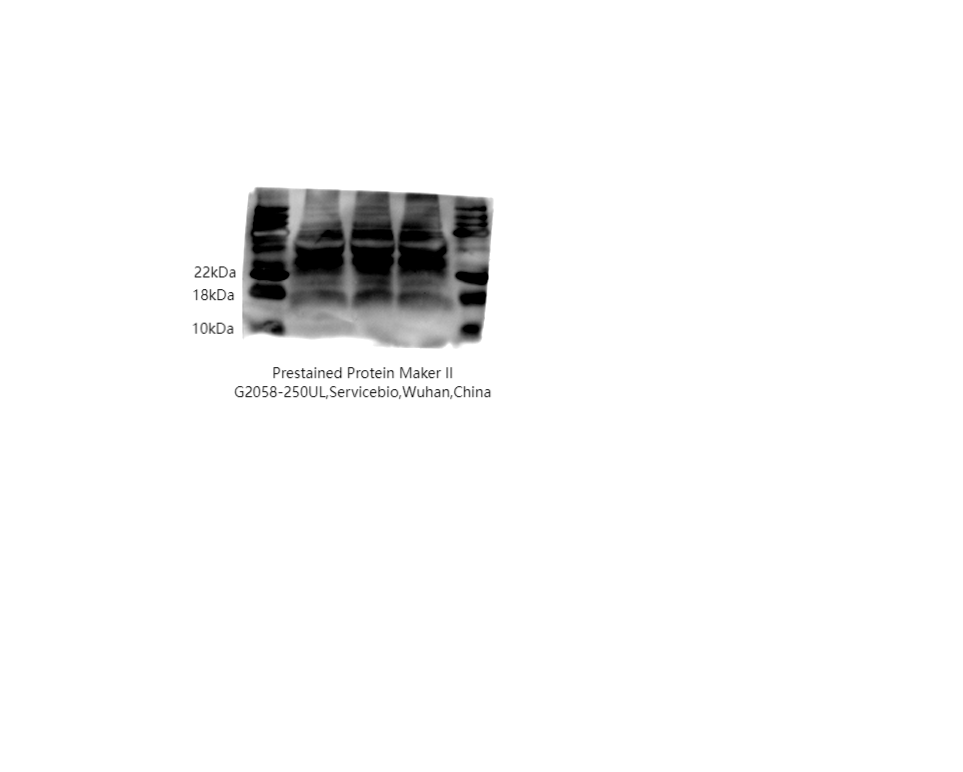

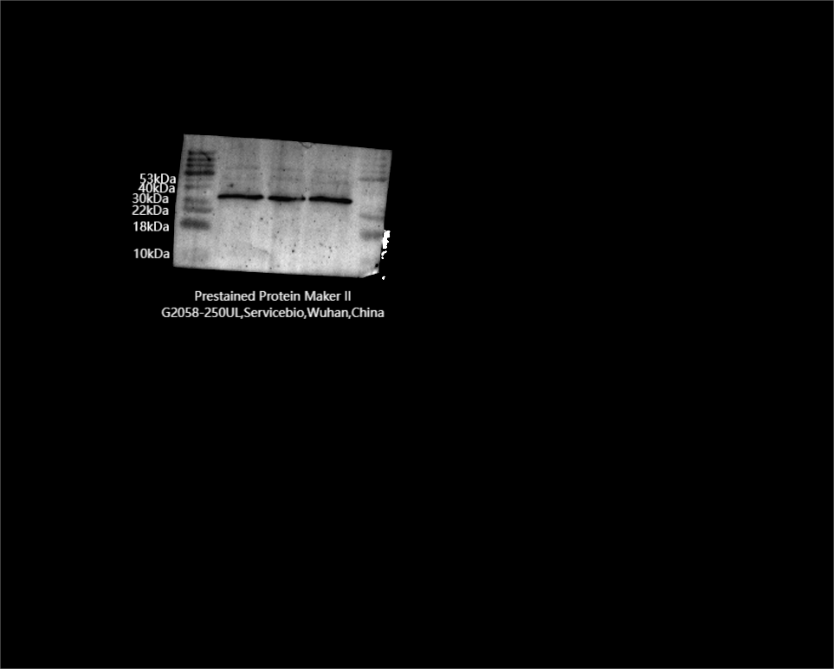


(5)
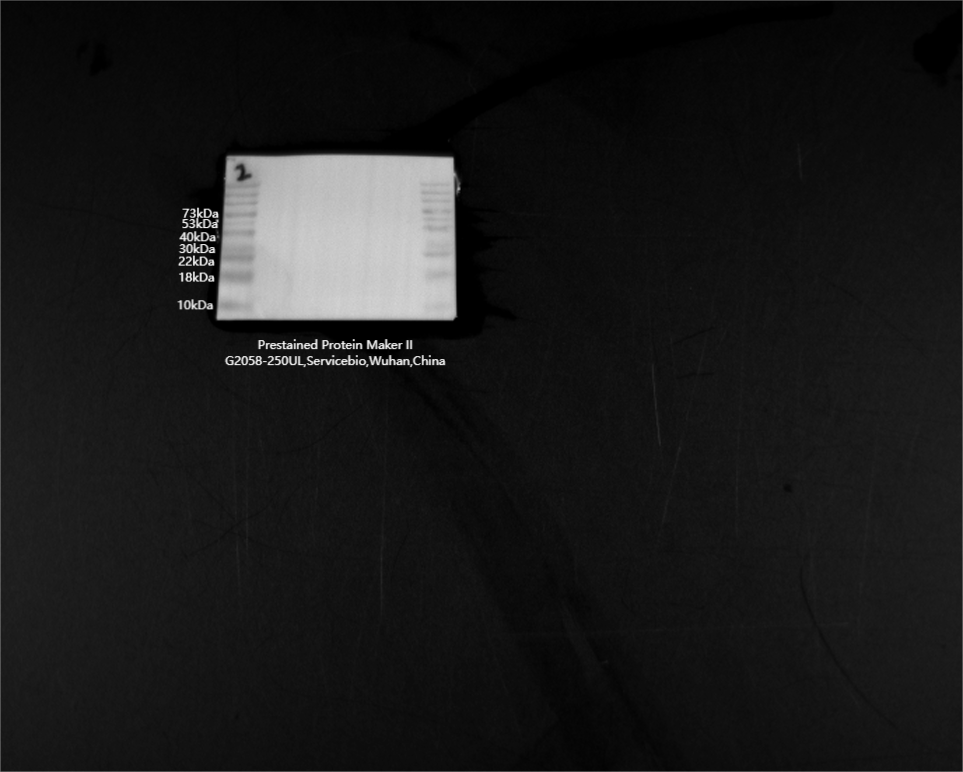

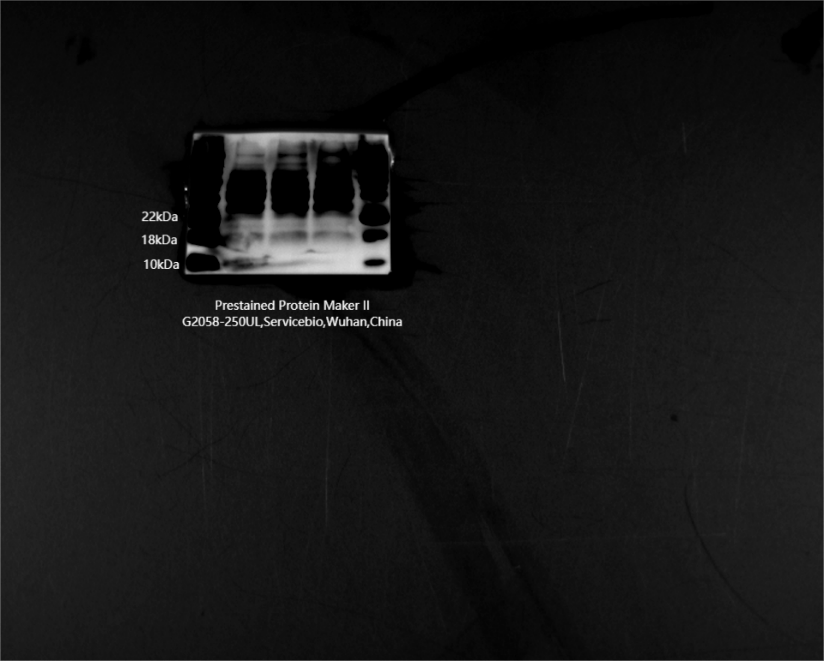

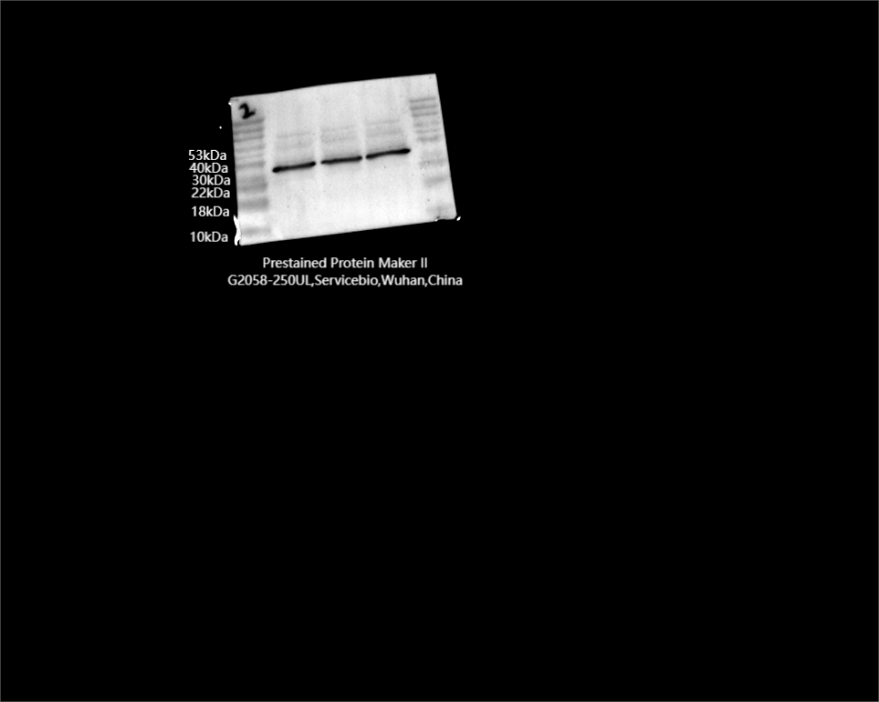


(6)
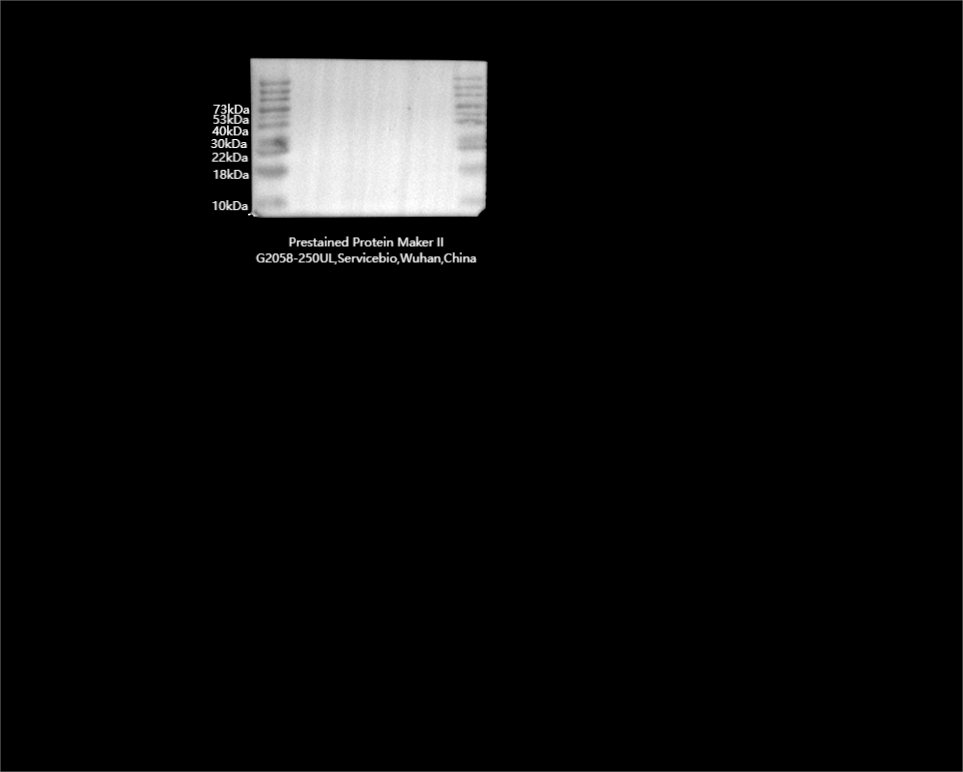

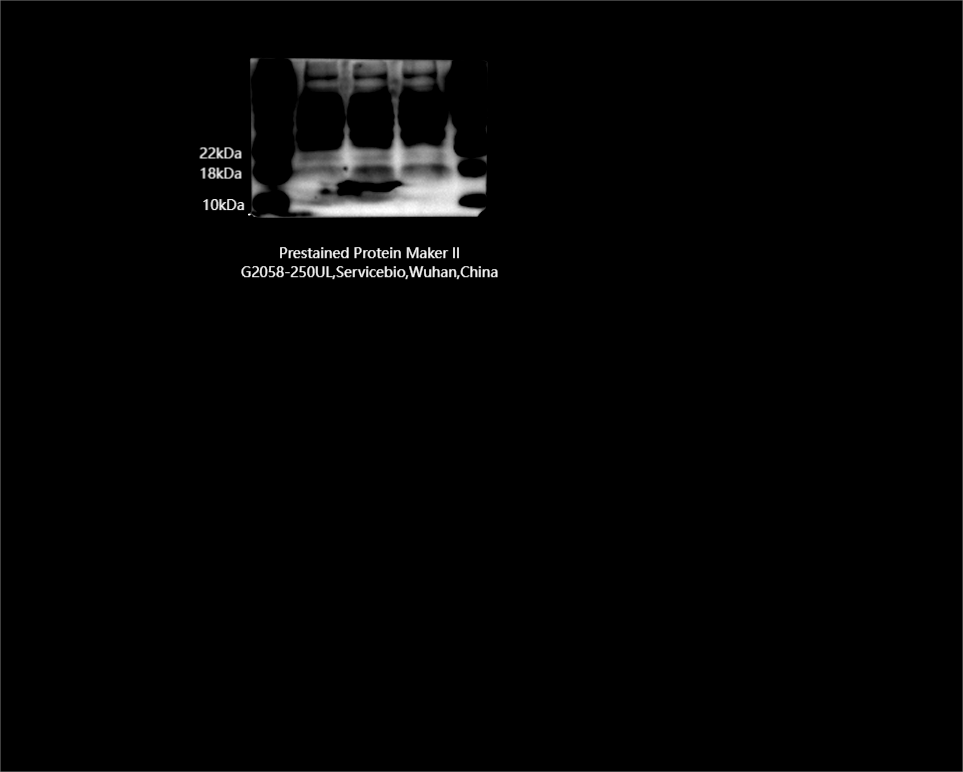

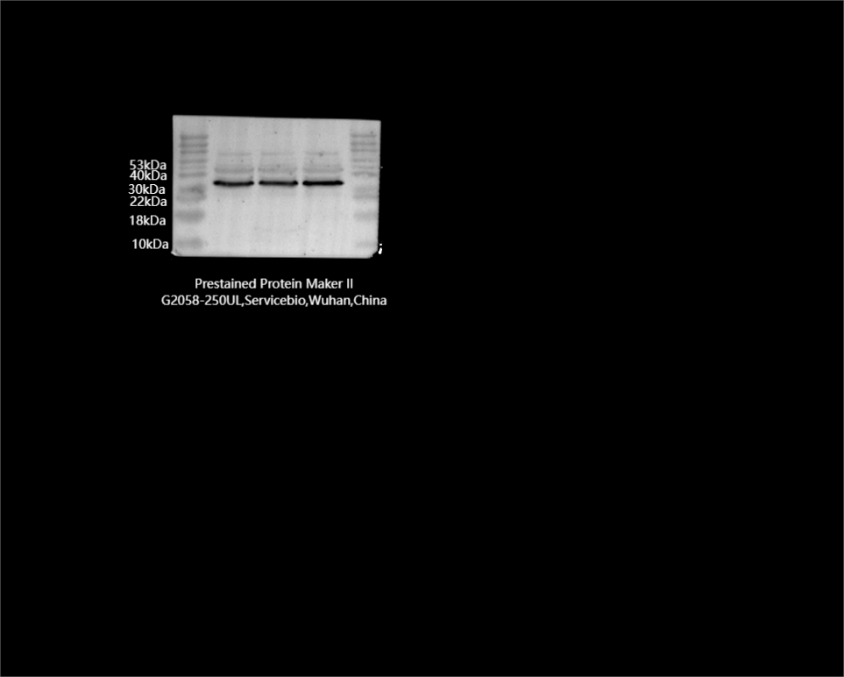


**ABCA1:**

(1)
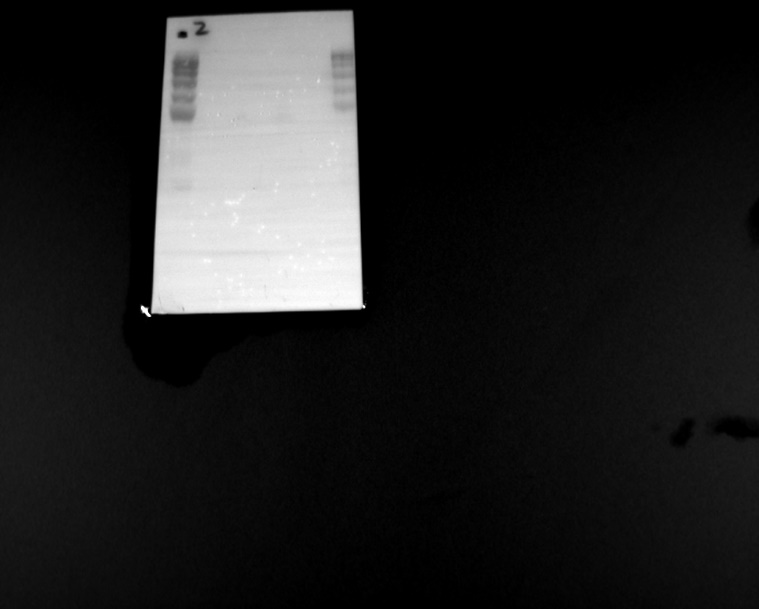

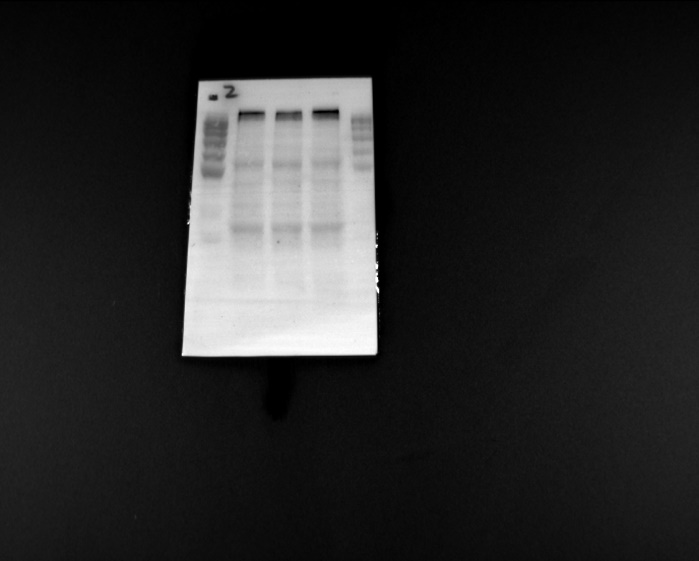

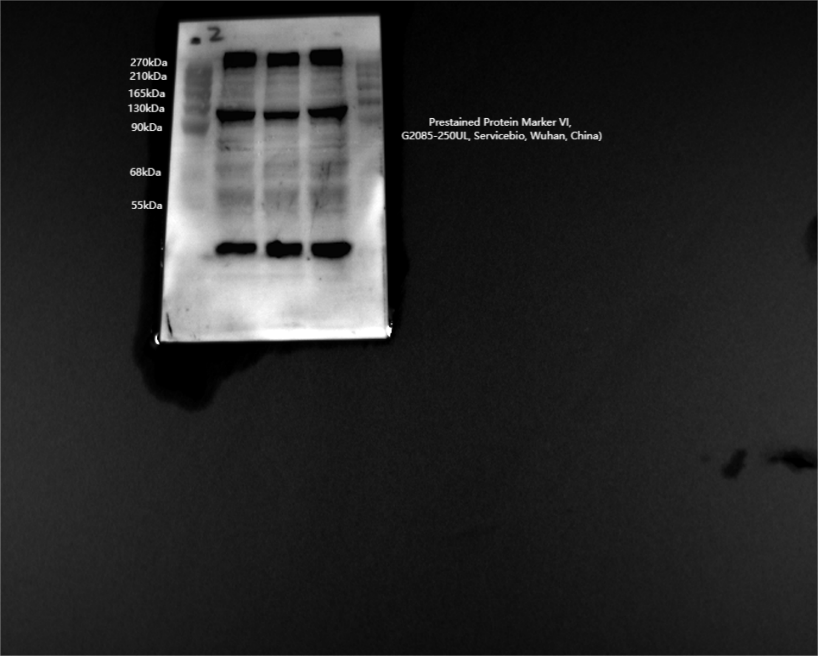


(2)
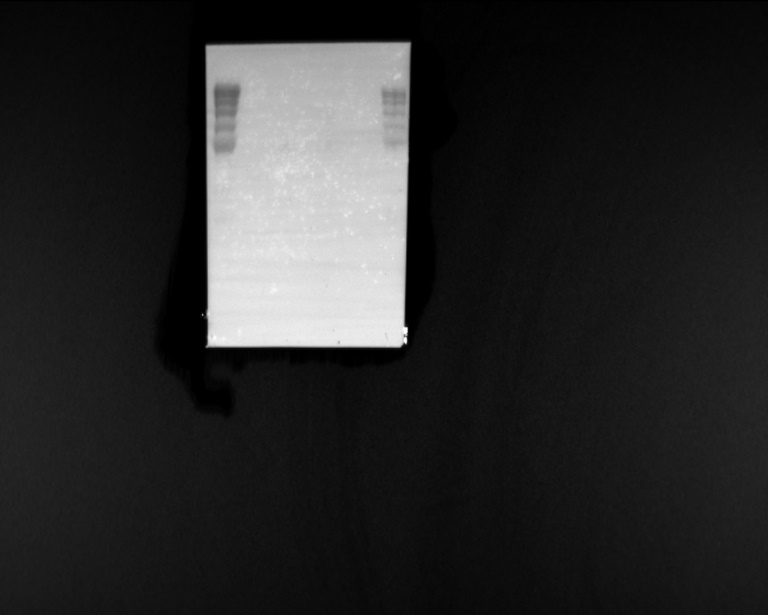

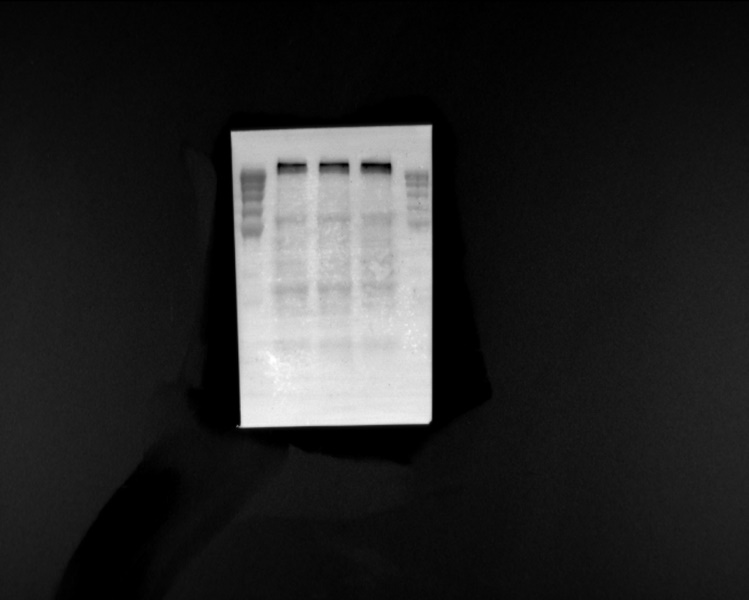

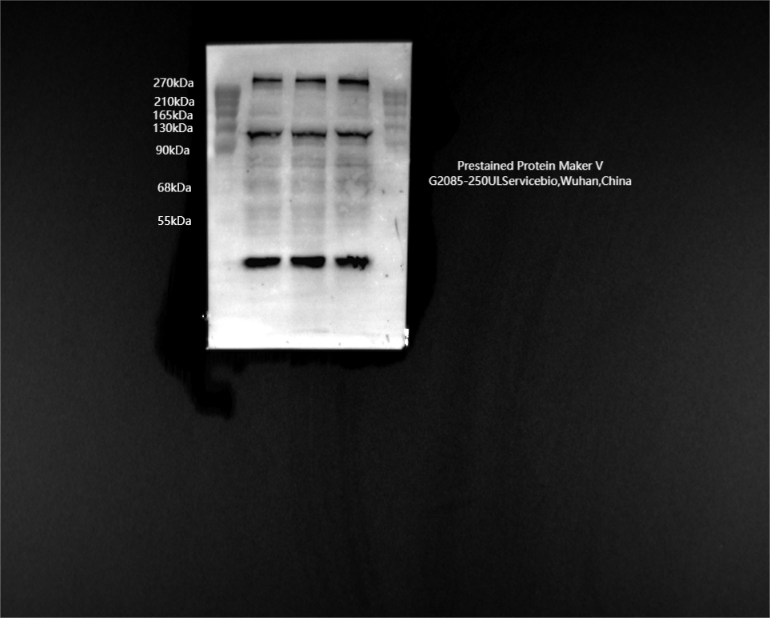


(3)
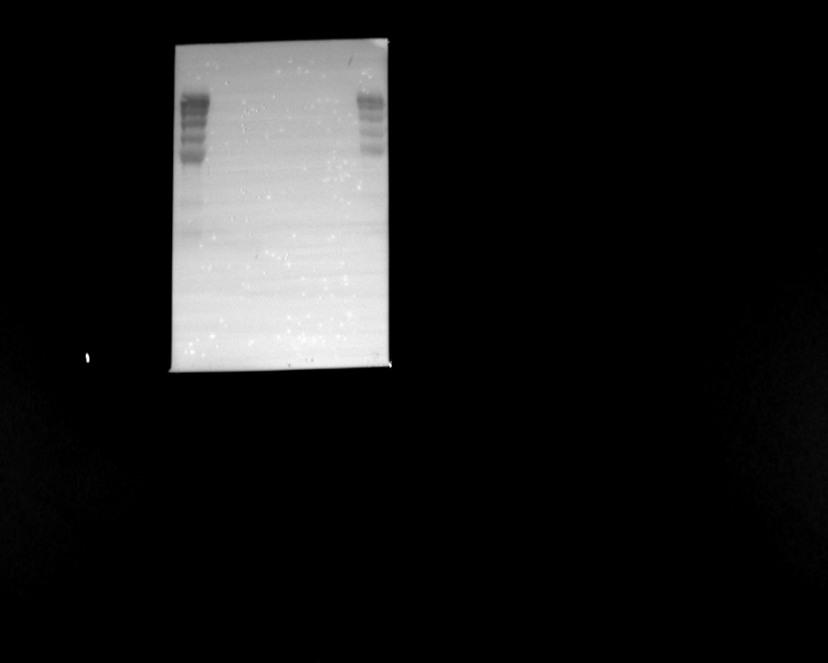

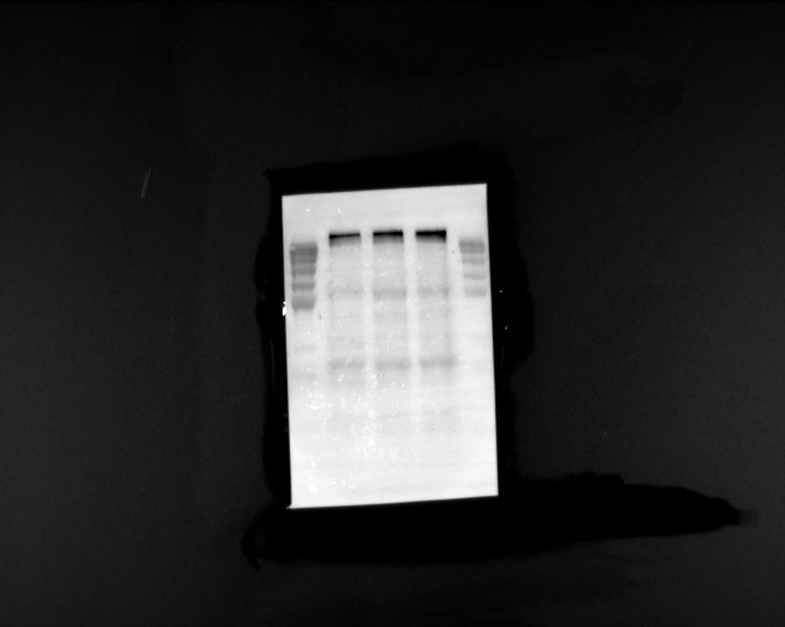

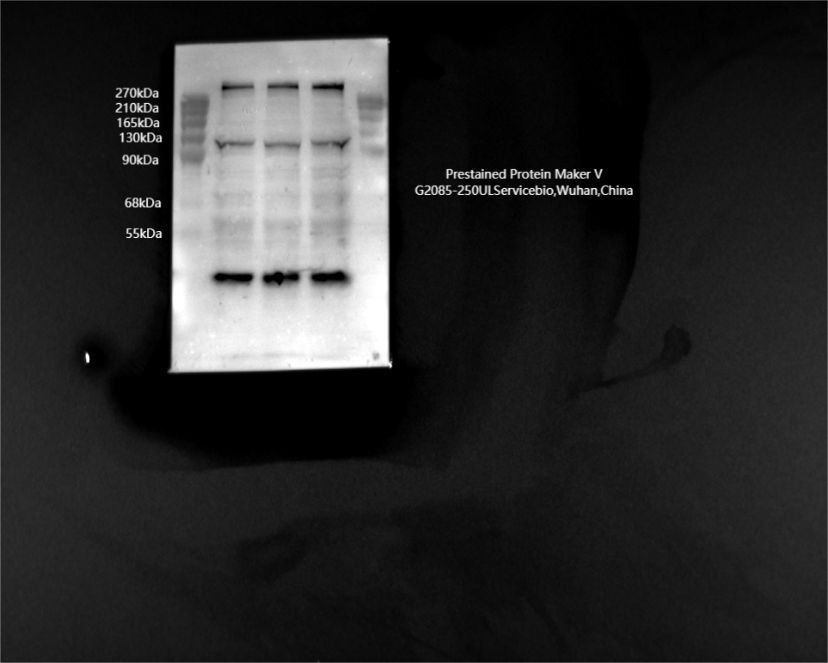


(4)
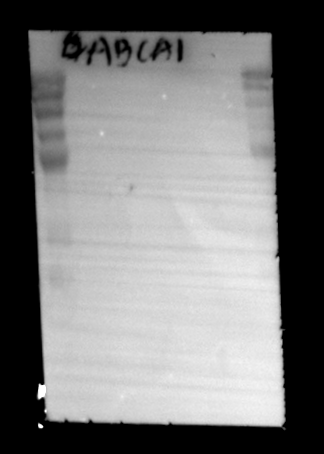

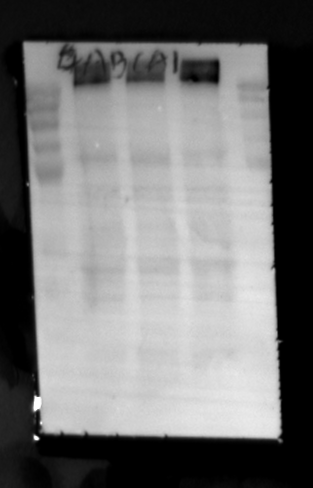

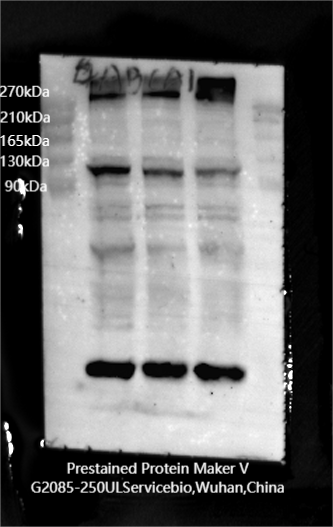


(5)
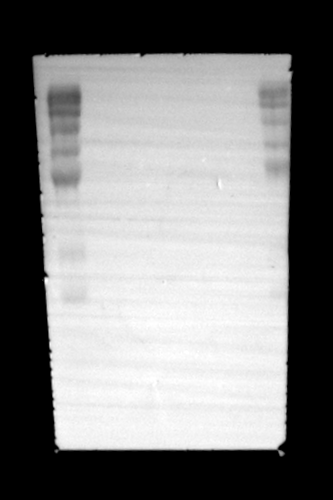

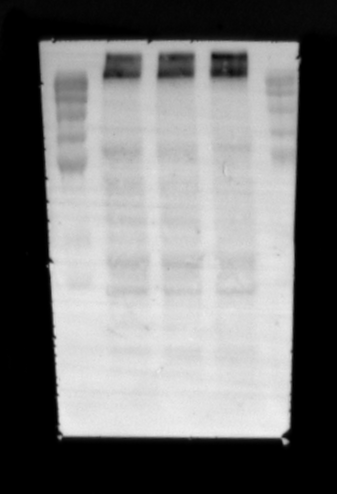

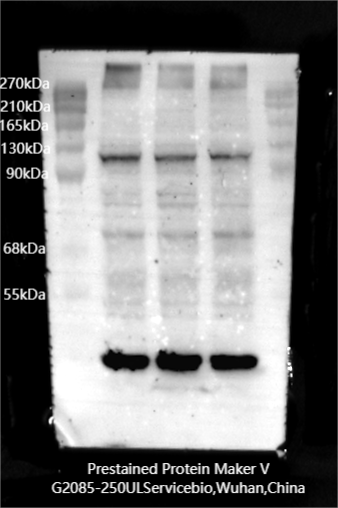


(6)
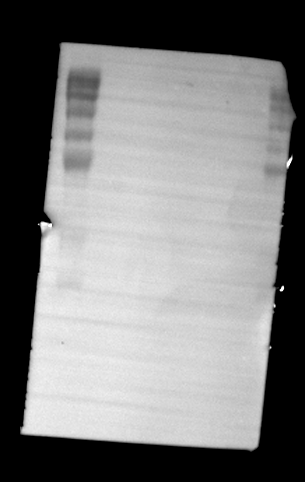

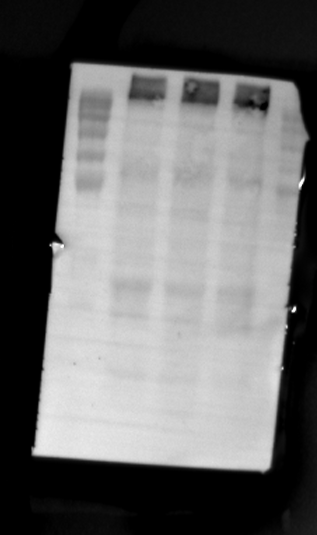

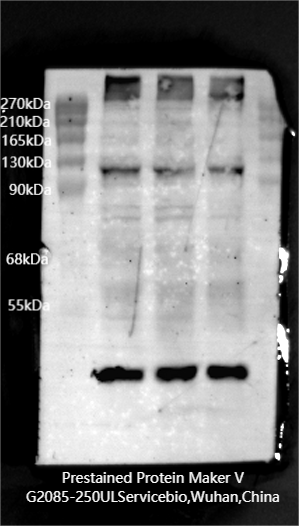


**CD206:**

(1)
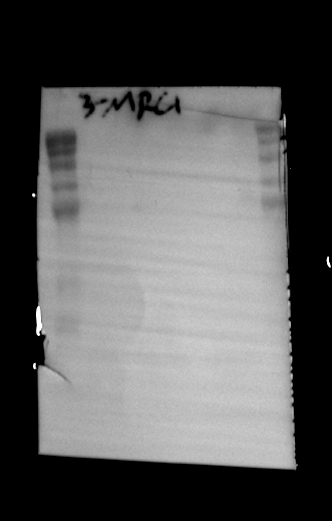

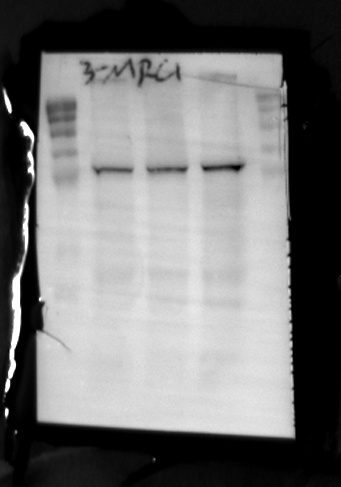

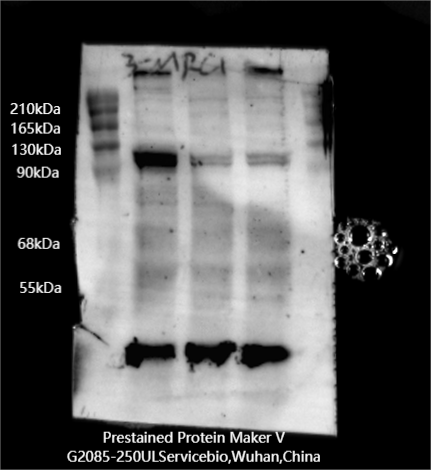


(2)
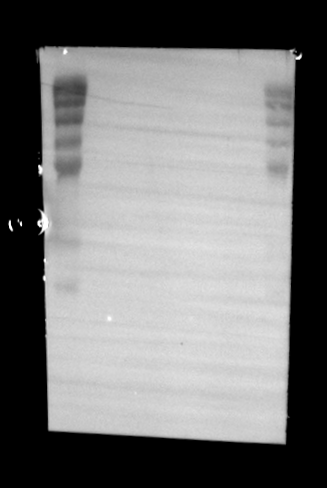

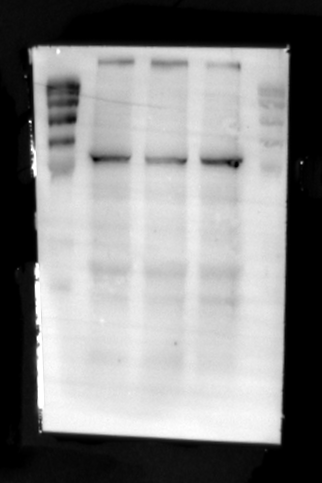

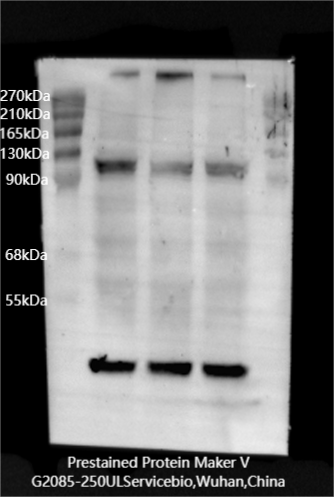


(3)
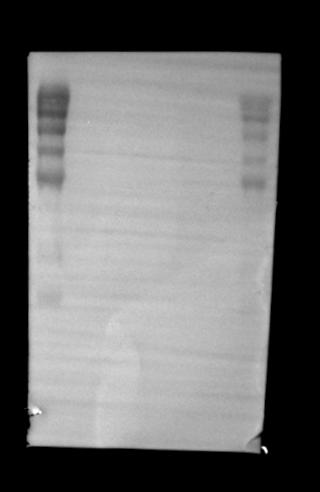

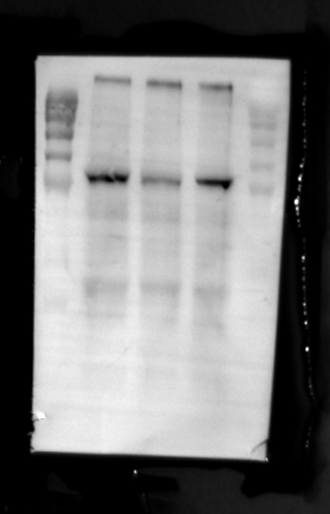

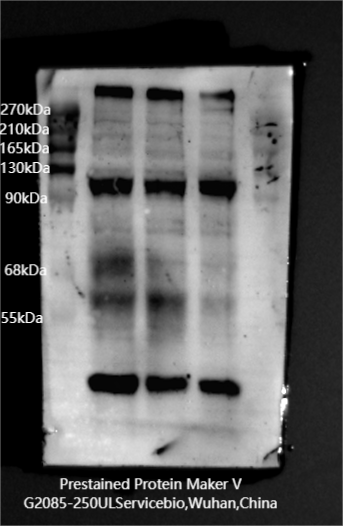


(4)
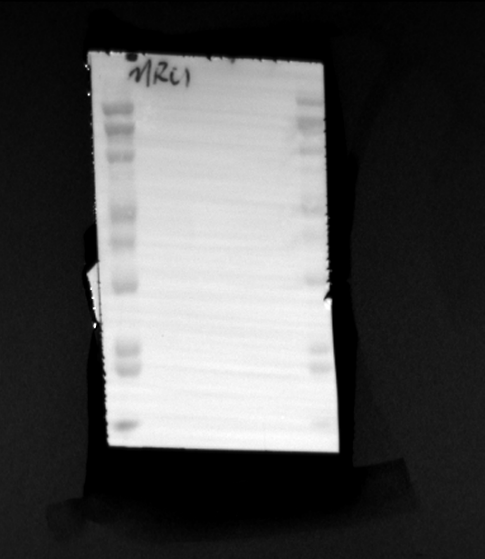

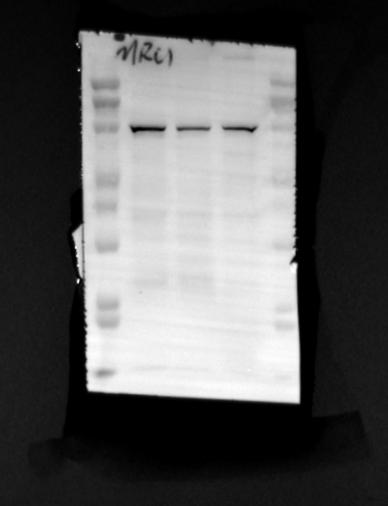

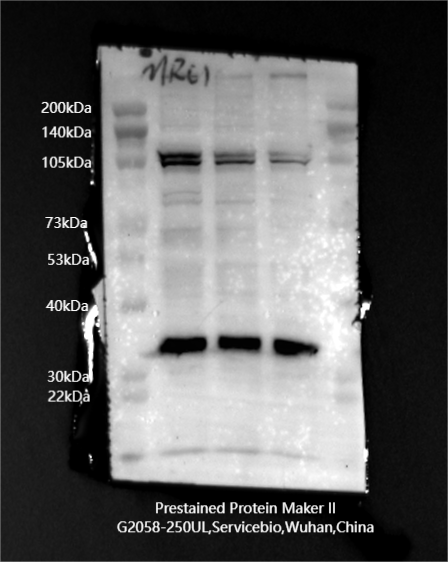


(5)
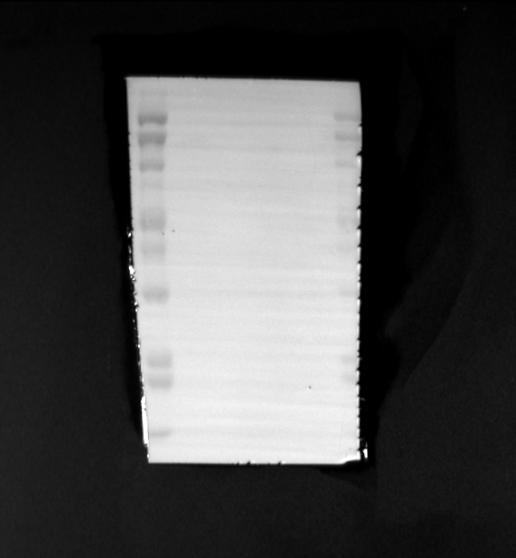

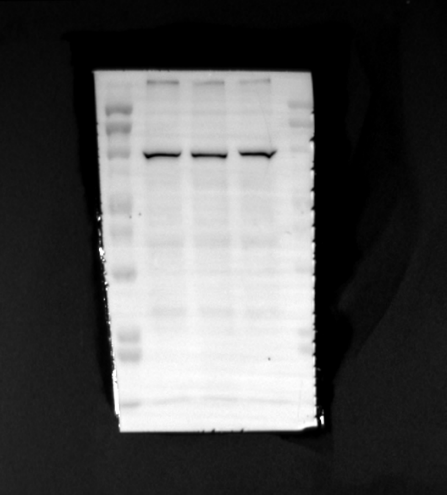

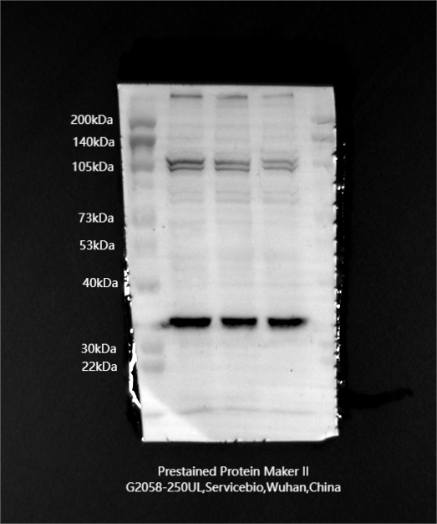


(6)
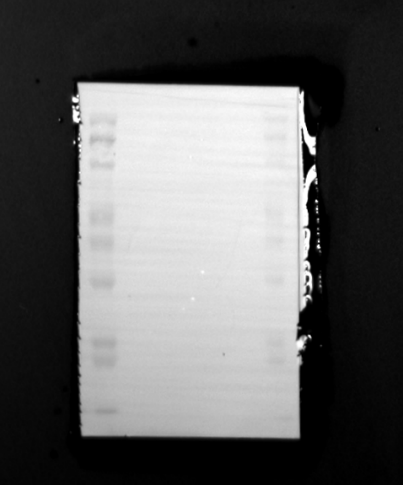

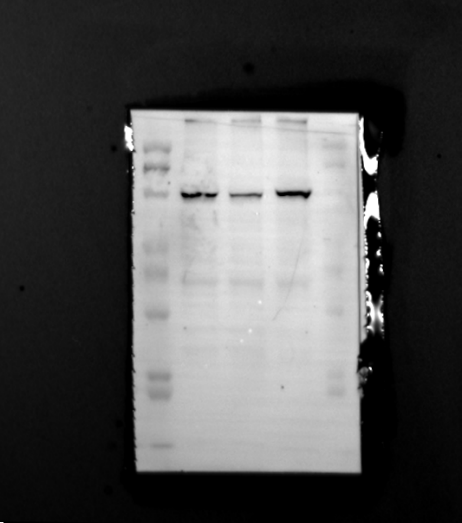

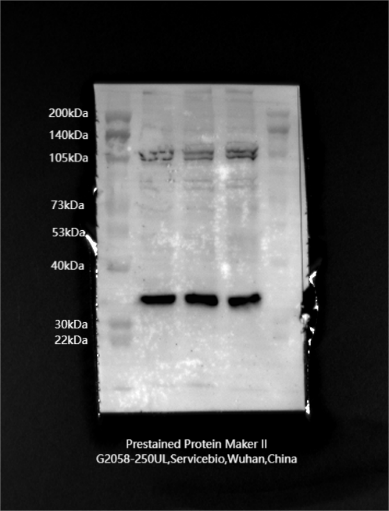

Supplement: Supplementary file 5 — Supplementary Material 5. [file 13041_2024_1135_MOESM5_ESM.docx]
