# Supplementary material for: Electroacupuncture reduces inflammatory damage following cerebral ischemia–reperfusion by enhancing ABCA1-mediated efferocytosis in M2 microglia
Source: Mol Brain. 2024 Sep 2;17:61. doi: 10.1186/s13041-024-01135-0 (PMC11367741; doi:10.1186/s13041-024-01135-0)
Supplement: Supplementary file 10 — Supplementary Material 10. [file 13041_2024_1135_MOESM10_ESM.docx]

**NeuN:**

The sham group

(1)
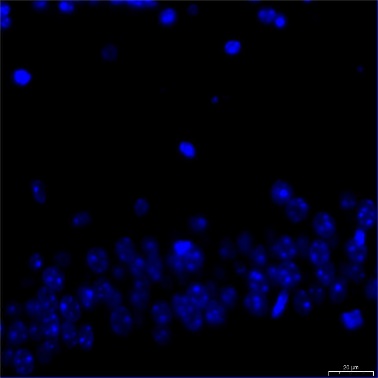

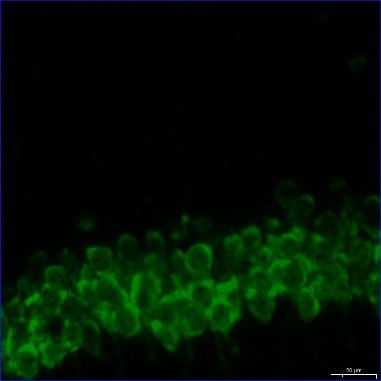

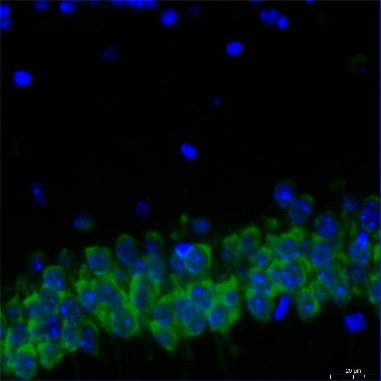


(2)
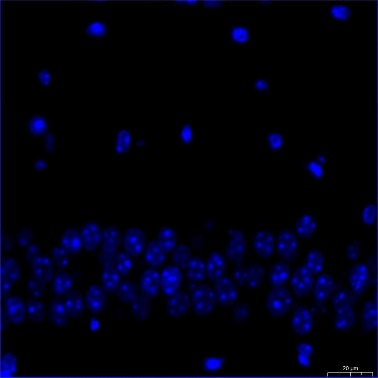

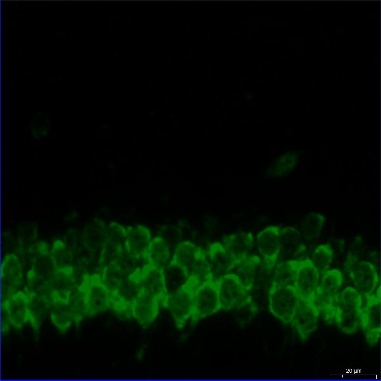

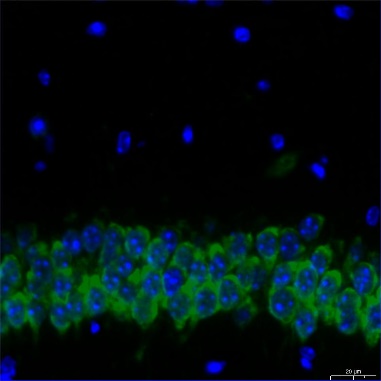


(3)
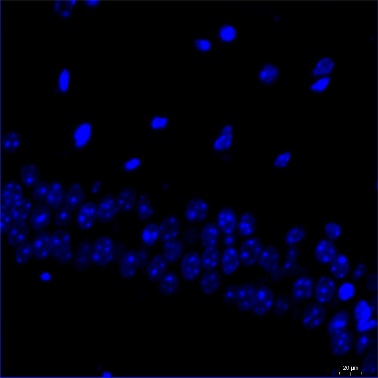

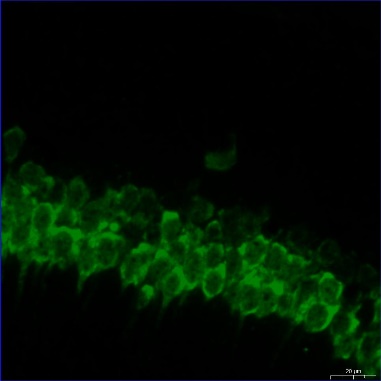

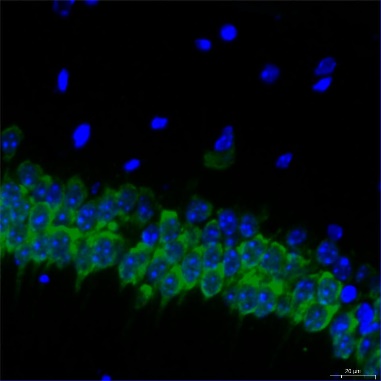


The I/R group

(1)
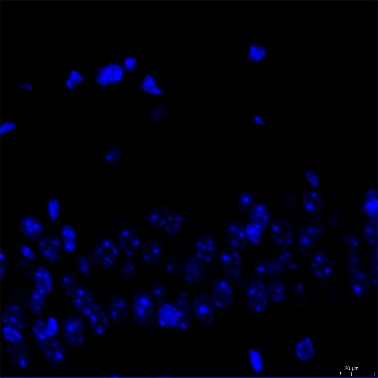

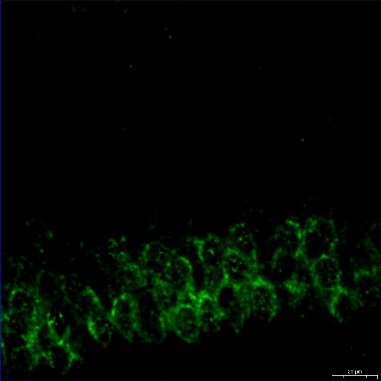

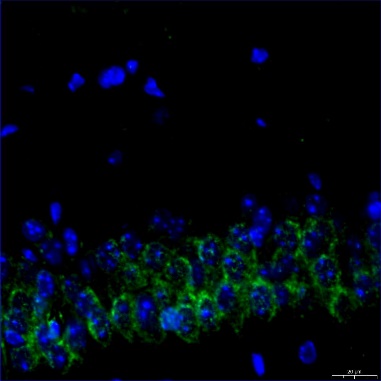


(2)
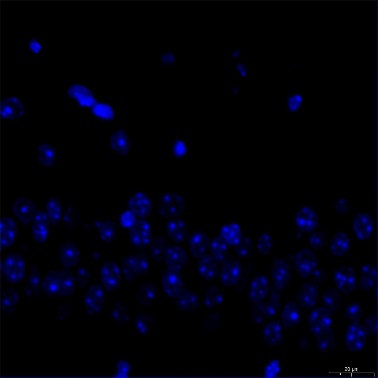

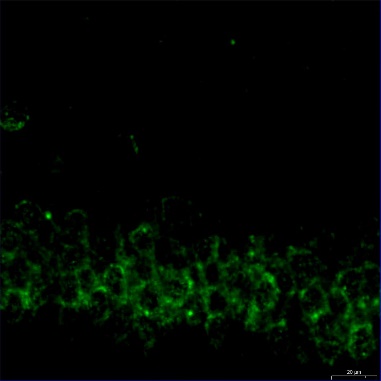

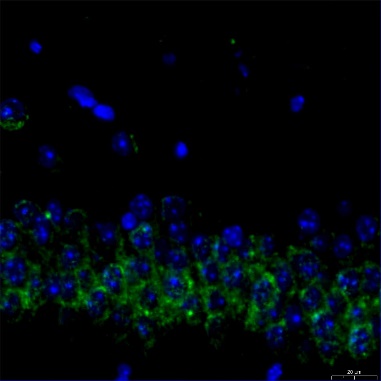


(3)
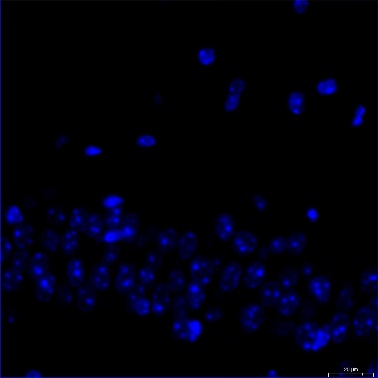

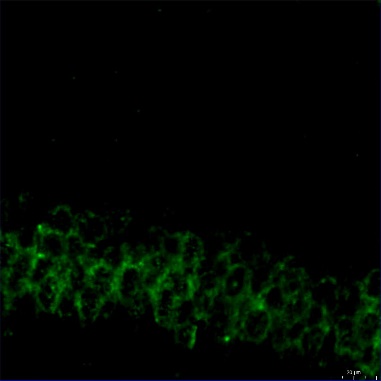

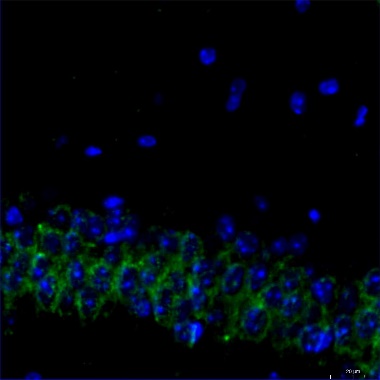


The EA group

(1)
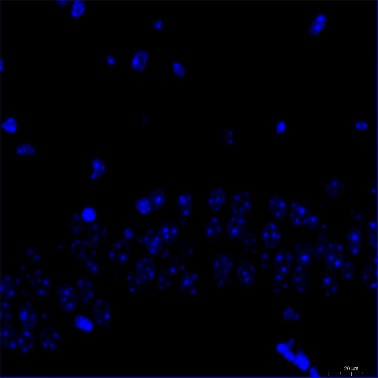

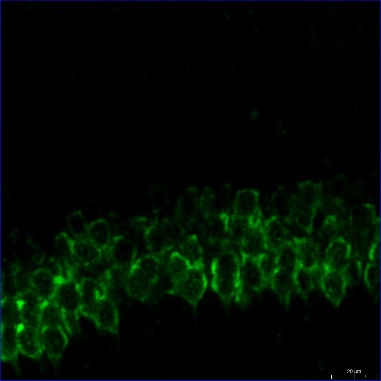

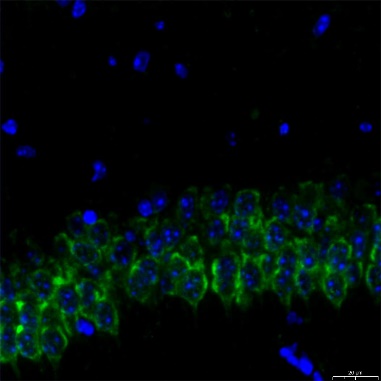


(2)
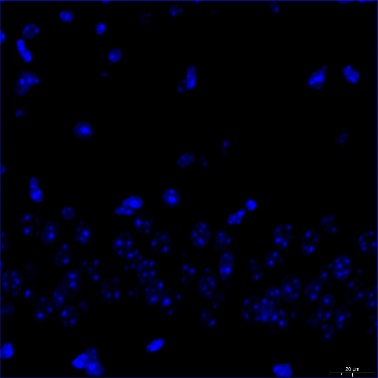

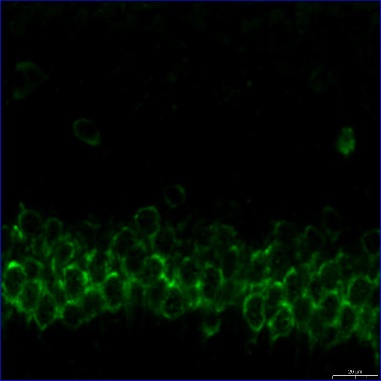

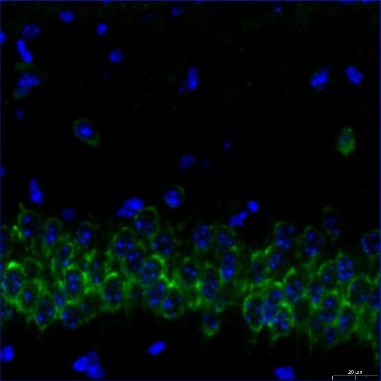


(3)
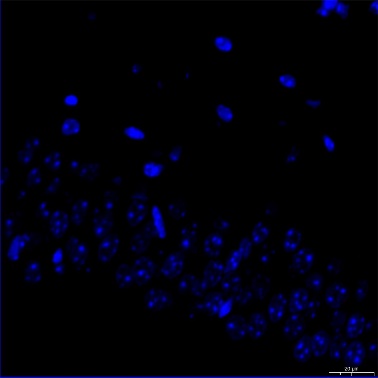

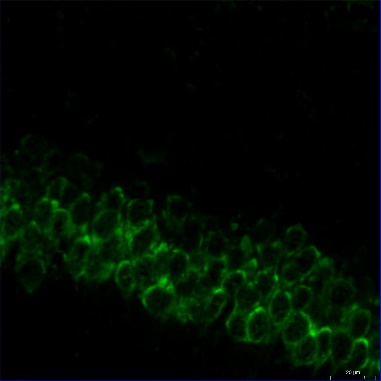

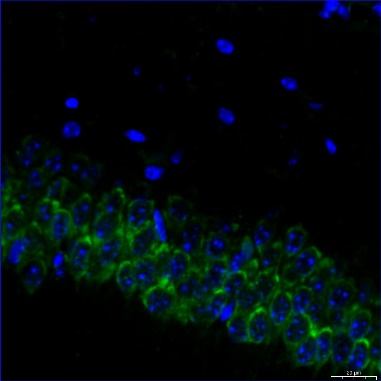


**Iba1:**

The sham group

(1)
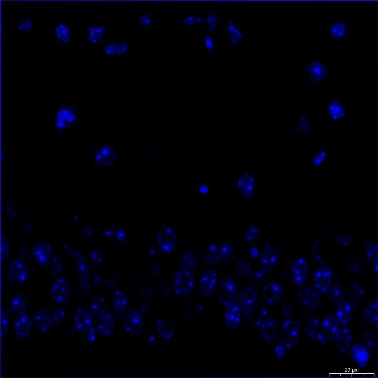

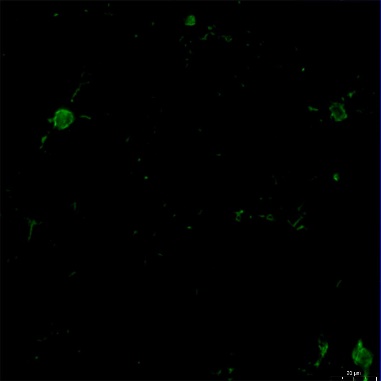

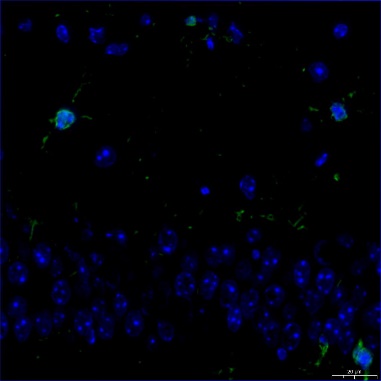


(2)
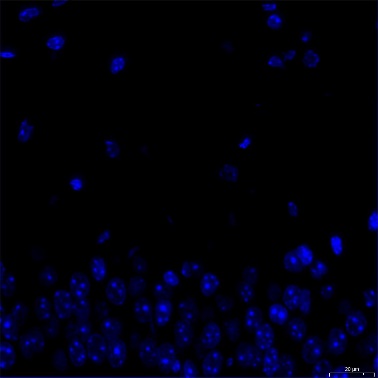

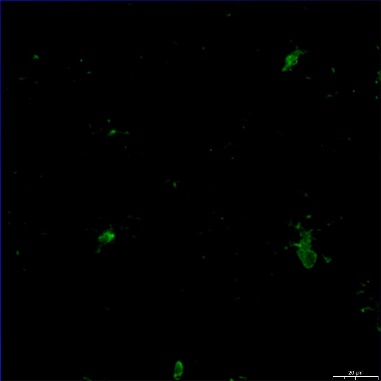

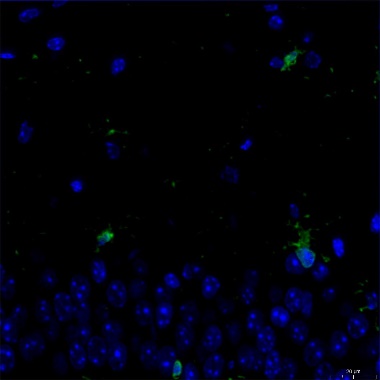


(3)
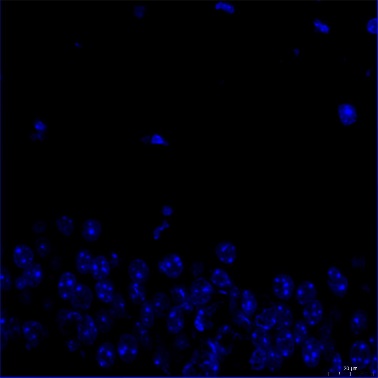

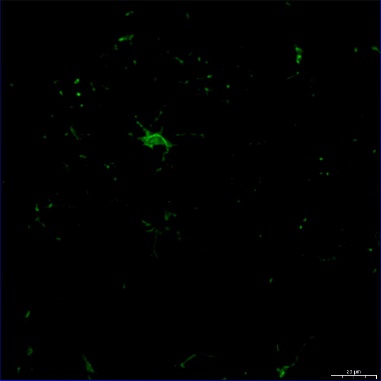

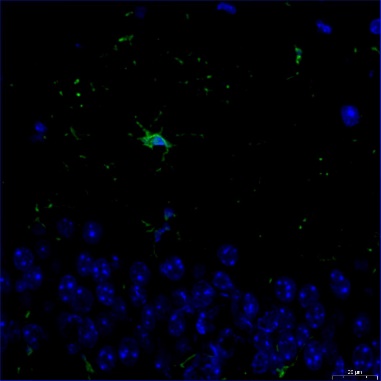


The I/R group

(1)
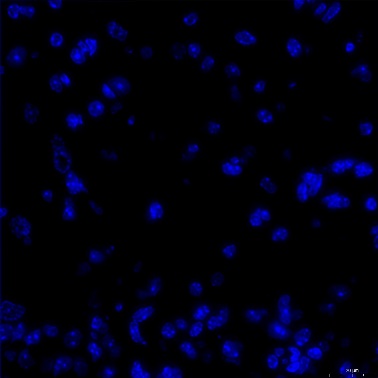

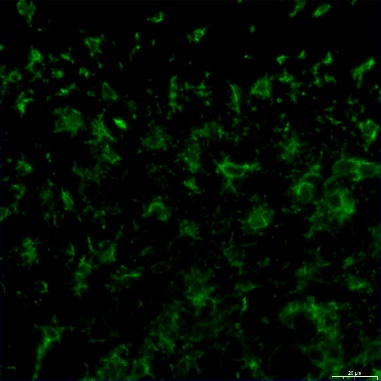

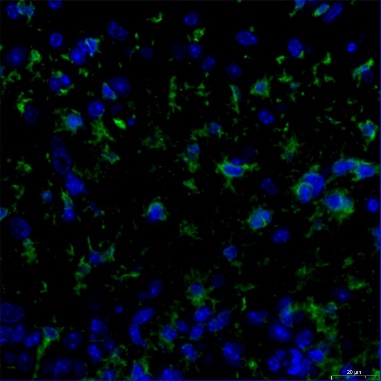


(2)
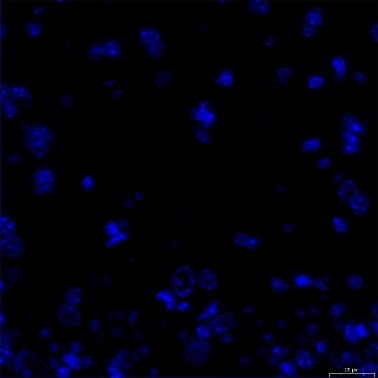

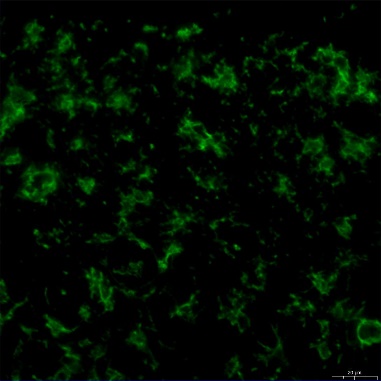

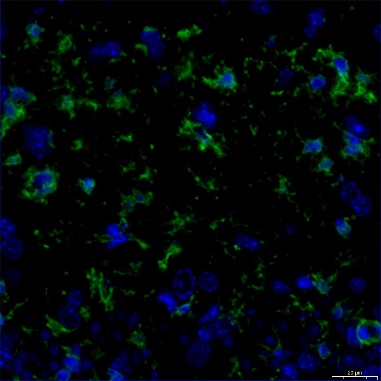


(3)
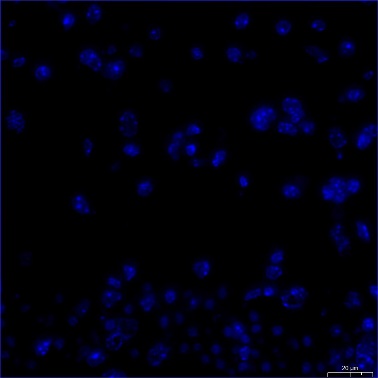

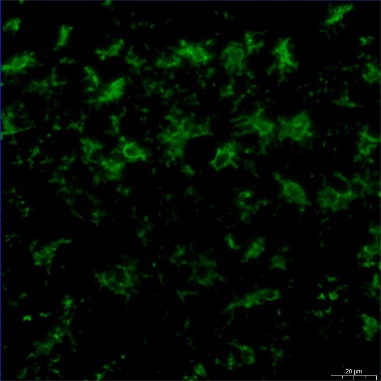

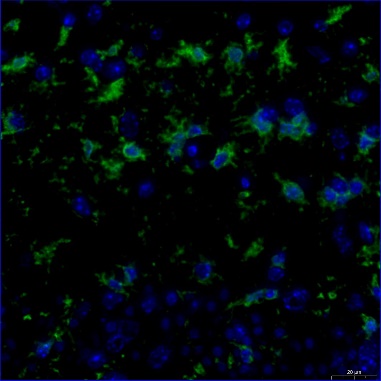


The EA group

(1)
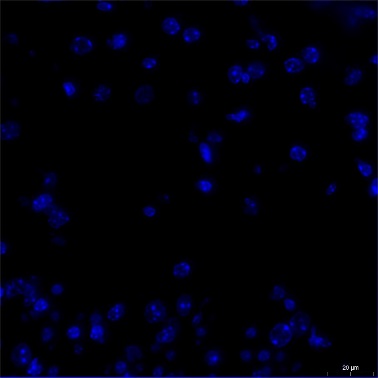

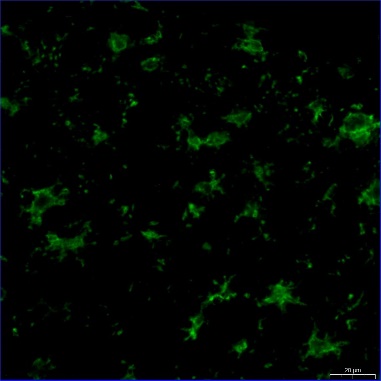

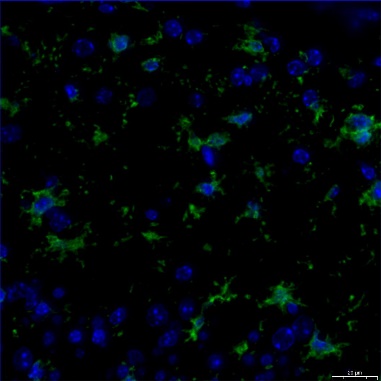


(2)
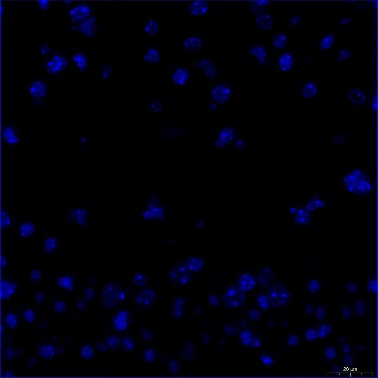

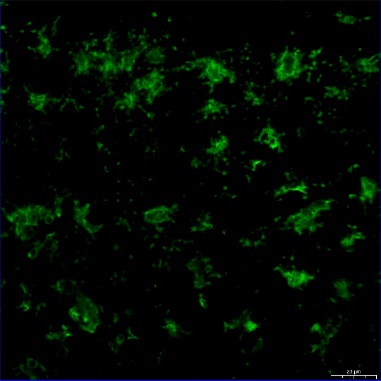

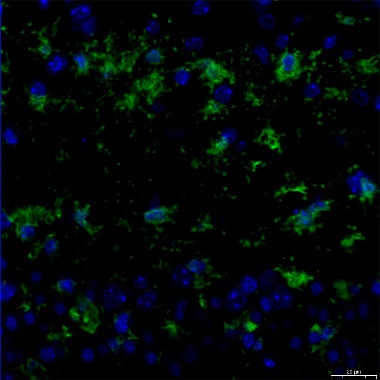


(3)
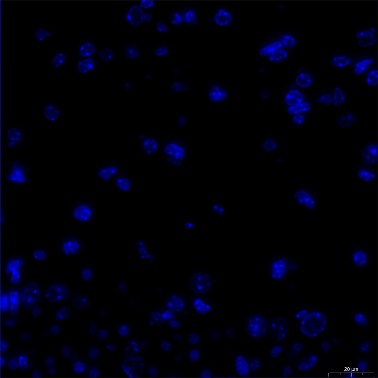

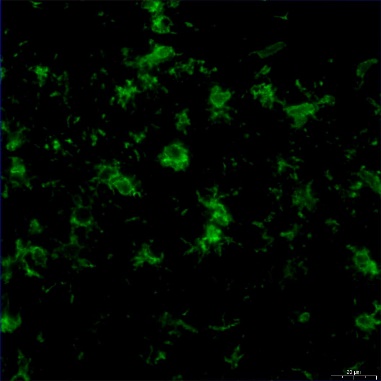

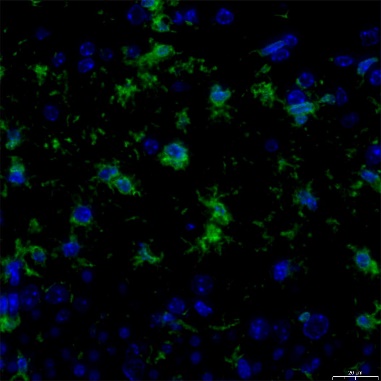


**Iba1+Cd206:**

The sham group

(1)
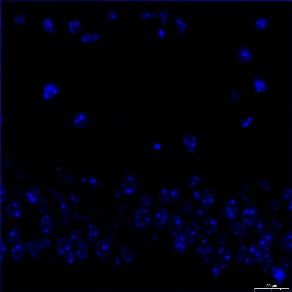

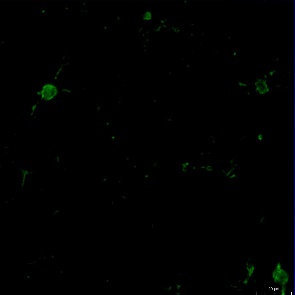

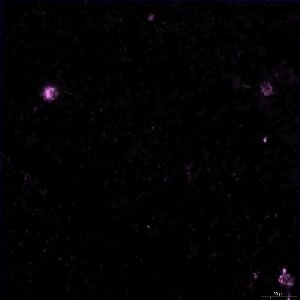

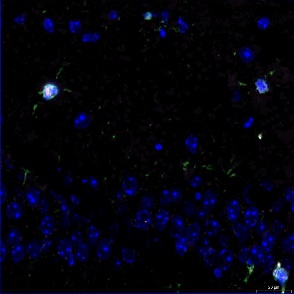


(2)
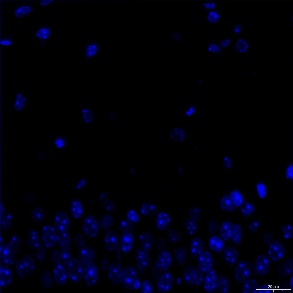

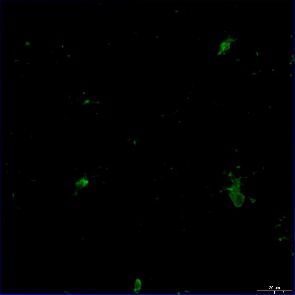

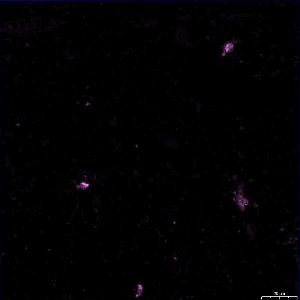

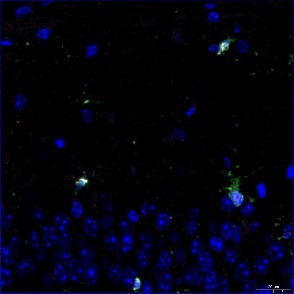


(3)
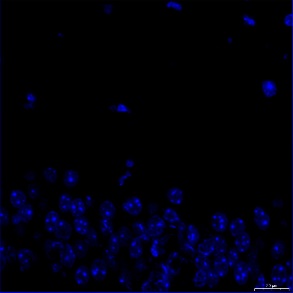

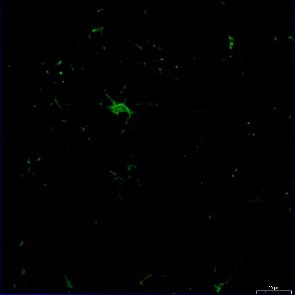

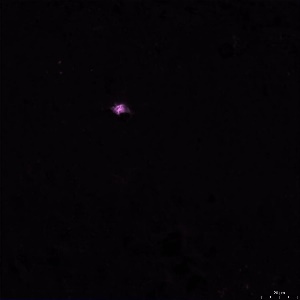

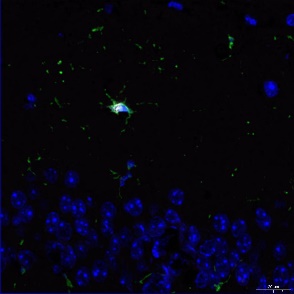


The I/R group

(1)
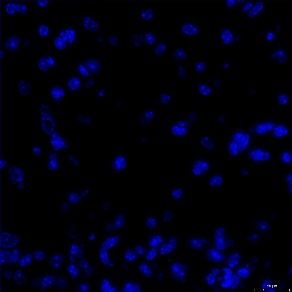

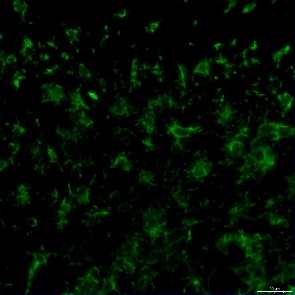

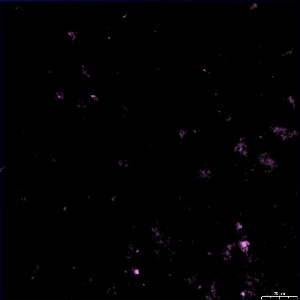

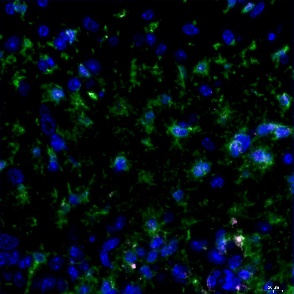


(2)
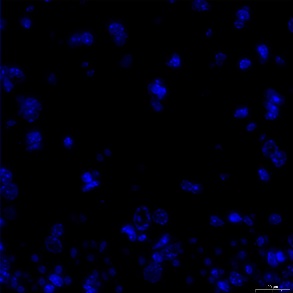

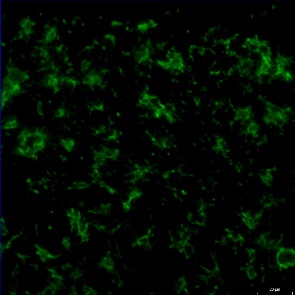

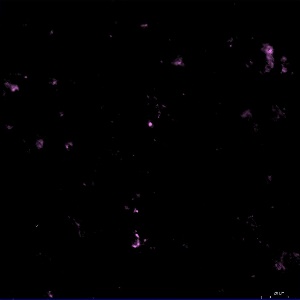

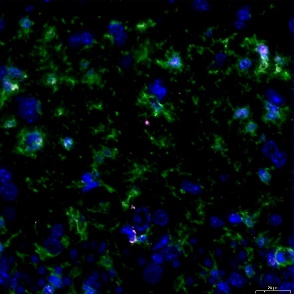


(3)
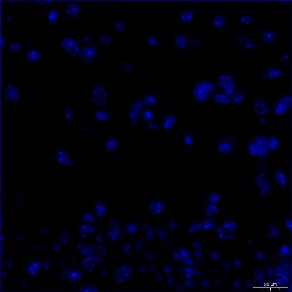

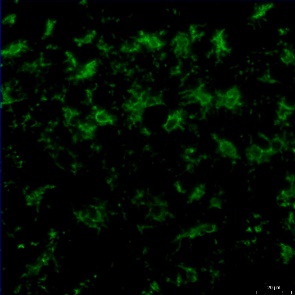

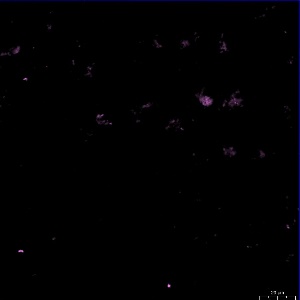

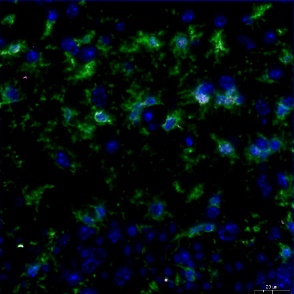


The EA group

(1)
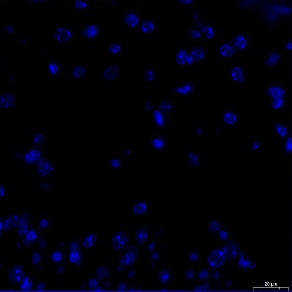

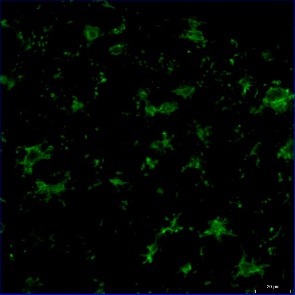

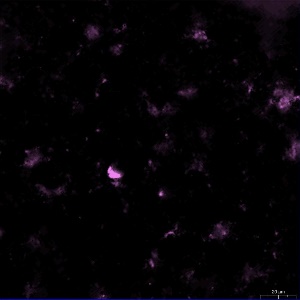

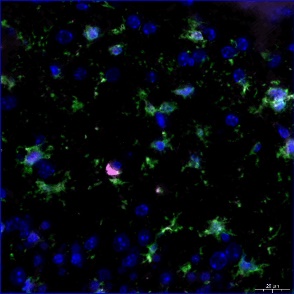


(2)
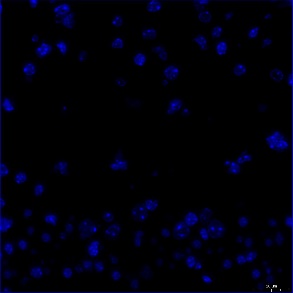

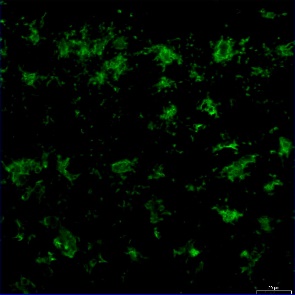

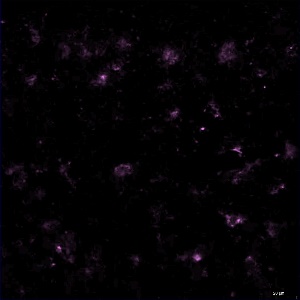

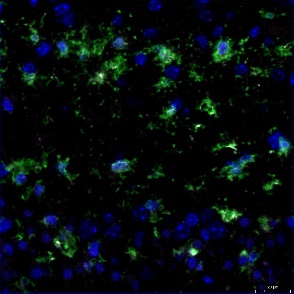


(3)
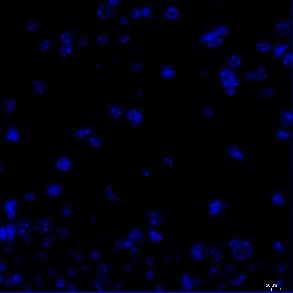

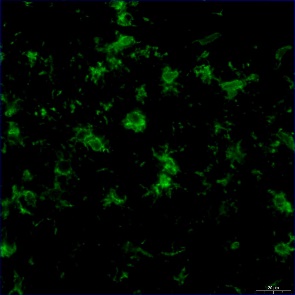

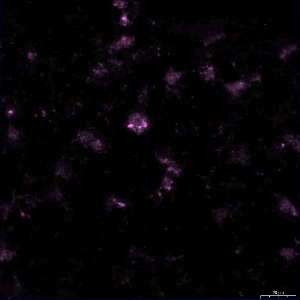

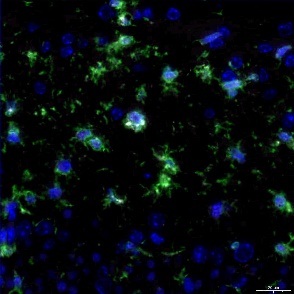


**Abca1+NeuN+Cd206:**

The sham group

(1)
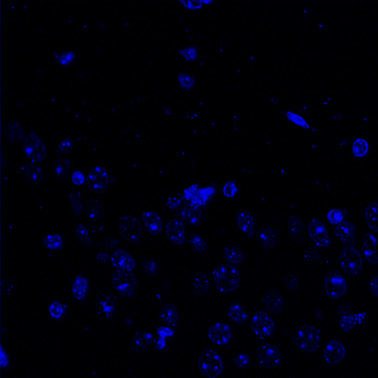

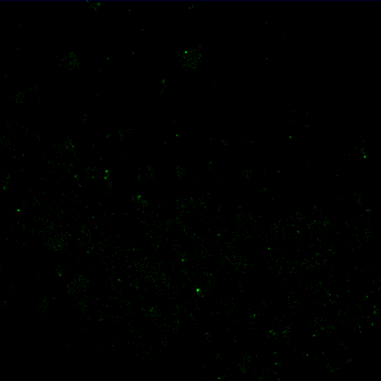

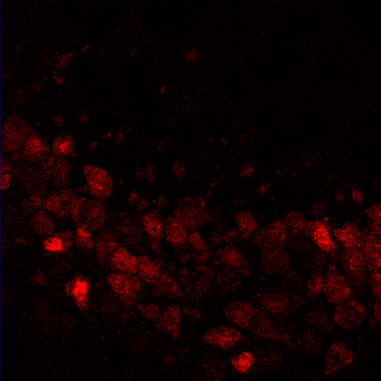

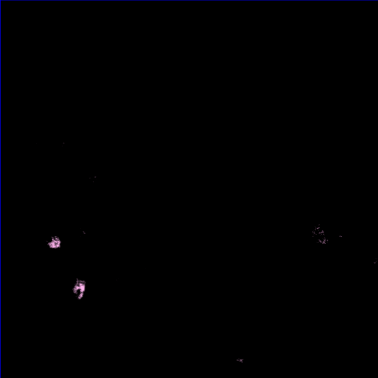

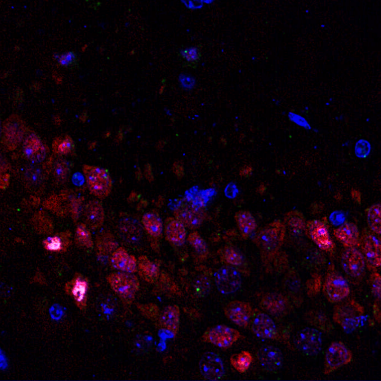


(2)
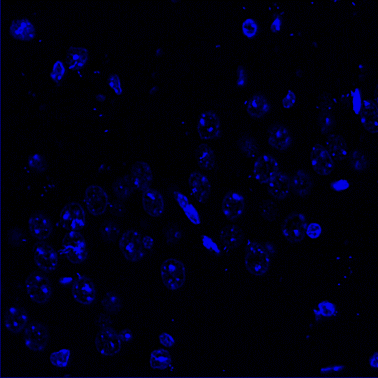

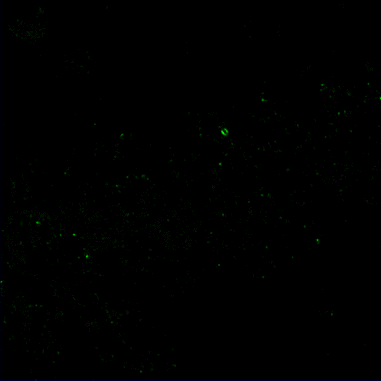

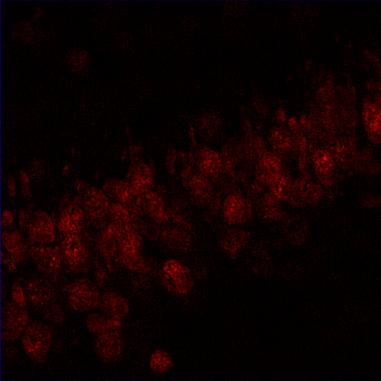

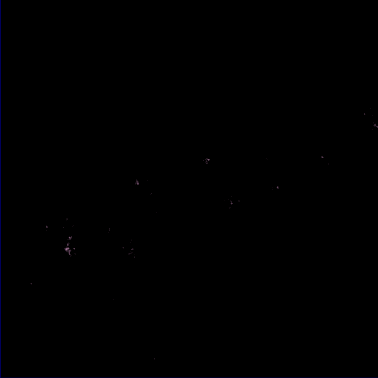

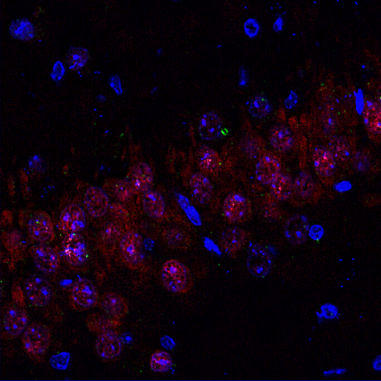


(3)

The I/R group

(1)

(2)

(3)

The EA group

(1)

(2)

(3)
